# Population-based analysis of *POT1* variants in a cutaneous melanoma case–control cohort

**DOI:** 10.1136/jmg-2022-108776

**Published:** 2022-12-20

**Authors:** Irving Simonin-Wilmer, Raul Ossio, Emmett M Leddin, Mark Harland, Karen A Pooley, Mauricio Gerardo Martil de la Garza, Sofia Obolenski, James Hewinson, Chi C Wong, Vivek Iyer, John C Taylor, Julia A Newton-Bishop, D Timothy Bishop, Gerardo Andrés Cisneros, Mark M Iles, David J Adams, Carla Daniela Robles-Espinoza

**Affiliations:** 1 Laboratorio Internacional de Investigación sobre el Genoma Humano, Universidad Nacional Autónoma de México, Campus Juriquilla, Querétaro, Qro, Mexico; 2 Department of Chemistry, University of North Texas, Denton, Texas, USA; 3 Section of Epidemiolgy and Biostatistics, Leeds Institute of Molecular Medicine, University of Leeds, Leeds, UK; 4 Centre for Cancer Genetic Epidemiology, Cambridge University, Cambridge, UK; 5 Department of Chemistry and Biochemistry, The University of Texas at Dallas, Richardson, Texas, USA; 6 CASM, Wellcome Sanger Institute, Hinxton, UK; 7 CeGaT GmbH, Tübingen, Germany; 8 Leeds Institute of Medical Research, University of Leeds, Leeds, UK; 9 Leeds Institute for Data Analytics, University of Leeds, Leeds, UK; 10 Section of Epidemiology and Biostatistics, University of Leeds, Leeds, UK; 11 Department of Physics, The University of Texas at Dallas, Richardson, Texas, USA; 12 Leeds Institute of Cancer and Pathology, University of Leeds, Leeds, UK

**Keywords:** Genetic Predisposition to Disease, Genetics, Genetic Variation, Genomics

## Abstract

Pathogenic germline variants in the protection of telomeres 1 gene (*POT1*) have been associated with predisposition to a range of tumour types, including melanoma, glioma, leukaemia and cardiac angiosarcoma. We sequenced all coding exons of the *POT1* gene in 2928 European-descent melanoma cases and 3298 controls, identifying 43 protein-changing genetic variants. We performed POT1-telomere binding assays for all missense and stop-gained variants, finding nine variants that impair or disrupt protein–telomere complex formation, and we further define the role of variants in the regulation of telomere length and complex formation through molecular dynamics simulations. We determine that *POT1* coding variants are a minor contributor to melanoma burden in the general population, with only about 0.5% of melanoma cases carrying germline pathogenic variants in this gene, but should be screened in individuals with a strong family history of melanoma and/or multiple malignancies.

Since the discovery of pathogenic alleles of *CDKN2A* 25 years ago,^1^ a number of other variants that increase melanoma risk have been uncovered by genome-wide association studies (GWAS)^2^ and the genomic analysis of melanoma-predisposed families. These variants affect biological pathways related to pigmentation (such as alleles of *MC1R*, the *‘red hair’ gene*), naevus count, including genetic variation adjacent to *PLA2G6*, cell cycle and senescence, comprising changes in *CDKN2A* and *CDK4*, and telomere regulation.^3^ Of note, pathogenic variants in the protection of telomeres 1 gene (*POT1*) have been associated with melanoma, as well as other types of cancer such as glioma,^4^ leukaemia^5^ and lymphoma.^6^ As such, pathogenic germline *POT1* variants have recently been recognised as defining a novel tumour predisposition syndrome.^7^ Genetic variation proximal to *POT1* has also been found to be associated with melanoma in recent large-scale GWAS studies.^8^

*POT1* encodes a single-stranded DNA (ssDNA)–binding protein that forms part of the shelterin complex, a group of proteins that have functions in telomere protection by allowing cells to distinguish the ends of chromosomes from sites of DNA damage and also function in regulating telomere length.^9^ In recent years, sequencing of melanoma-predisposed individuals has revealed a number of pathogenic alleles of *POT1* which affect the ability of POT1 to bind to ssDNA and therefore lead to longer and abnormal telomeres.^10–12^ This, in turn, may promote carcinogenesis through the accumulation of damage at chromosome ends and a delay in the onset of cell senescence. Further, a recent study has identified *POT1* variants that lead to shorter telomeres,^13^ emphasising the need to identify and catalogue the consequences of these genetic changes in carriers.

As estimates have suggested that *POT1* may be the second major high-penetrance melanoma susceptibility gene after *CDKN2A*, being causal of disease predisposition in 2%–4% of *CDKN2A/CDK4*-negative families,^10 14^ it has been included in multiple panels for genetic testing of melanoma families. As such, and to inform genetic counselling, there is a need to identify which genetic variants abrogate POT1 function leading to telomere dysregulation, as well as to determine their frequency in population-ascertained melanoma cases. In this study, we performed experimental and bioinformatic analyses to identify germline variants that disrupt the POT1–ssDNA complex and lead to telomere length alterations.

This study included 2928 melanoma cases and 3298 controls, making up a total of 6226 European-descent (British) individuals from two distinct melanoma cohorts plus a population cohort (online supplemental methods). We sequenced all *POT1* coding exons on the MiSeq platform (reference transcript: ENST00000357628). After alignment, variant calling and quality filtering, we identified 43 protein-altering variants in *POT1* by Fluidigm PCR-based amplicon sequencing and validated them by target capture with Agilent SureSelect probes and Illumina sequencing (online supplemental methods, online supplemental figure 1, online supplemental table 1, online supplemental file 6). Of these, 19 have not been reported in the gnomAD 2.1 dataset.^15^

10.1136/jmg-2022-108776.supp1 Supplementary data

10.1136/jmg-2022-108776.supp2 Supplementary data

10.1136/jmg-2022-108776.supp6 Supplementary data

To assess whether the detected variants impair telomere regulation, we analysed the ability of in vitro-translated POT1 proteins containing all missense and stop-gained variants (38/43 variants in total (online supplemental table 1) to bind to a telomere-like oligo via electrophoretic mobility shift assay (EMSA) experiments (online supplemental methods)). Our results indicate that four variants completely disrupted POT1–ssDNA complex formation (p.Cys59Tyr, p.Arg137His, p.Leu259Ter and p.Arg273Leu), whereas a further five appear to reduce the affinity of the interaction (p.Lys39Asn, p.Lys85Thr, p.Ser99Pro, p.Arg117His and p.Asp224Asn) (figure 1A; online supplemental figure 2). Of these, six had not been reported in the gnomAD 2.1 dataset, and, of note, as expected, all variants that altered POT1-ssDNA binding fall within the *N*-terminal OB domains.

**Figure 1:**
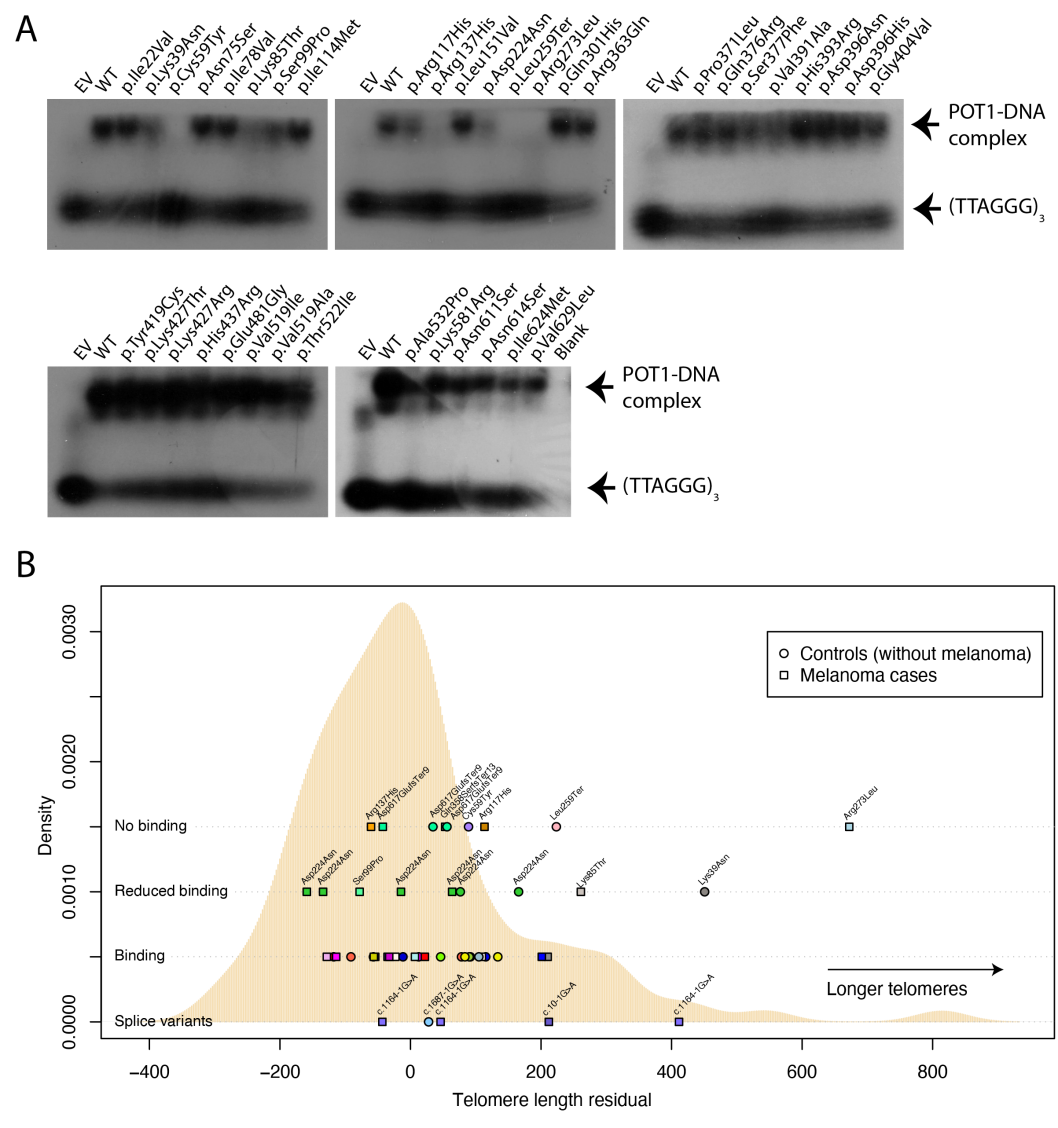
Biological consequences of protection of telomeres 1 gene (*POT1)* variants. (A) Electrophoretic mobility shift assays (EMSAs) are shown testing the ability of in vitro-translated mutant POT1 proteins to bind a telomere-like oligo (TTAGGGTTAGGGTTAGGG). EV, empty vector; WT, wild-type protein. (B) Telomere length of carriers of pathogenic *POT1* variants is depicted over a telomere length distribution of melanoma cases and controls with no pathogenic *POT1* variants. The distribution of the means of residuals from the linear model distribution of telomere lengths for individuals with no *POT1* variants is depicted in beige. The mean of the adjusted telomere lengths for individuals with *POT1* variants is shown on top according to the variant type (no binding, reduced binding or binding according to EMSA and splice variants). Melanoma cases are shown in squares and controls in circles. Each variant is shown in a different colour. For the ‘Binding’ row, the variants from left to right are p.Pro371Leu, p.Ile624Met, p.Asn611Ser, p.Lys427Thr, p.Asp396His, p.Val629Leu, p.His393Arg, p.Leu151Val, p.Asn75Ser, p.His393Arg, p.Lys581Arg, p.Glu481Gly, p.Ser377Phe, p.Asn614Ser, p.Tyr419Cys/p.Gly404Val, p.Asp396Asn, p.Ile78Val, p.Val519Ala, p.Thr522Ile, p.Ile114Met, p.Arg363Gln/p.Val391Ala, p.Lys427Arg, p.His437Arg, p.Val519Ile, p.His393Arg and p.Ala532Pro.

Variants were classified in three groups according to their pathogenicity: Group 1 variants were confirmed by EMSA to disrupt the POT1–ssDNA complex or were those strongly suspected as pathogenic (frameshift and splice acceptor variants). We included variants with reduced binding in this category due to their high conservation across species (online supplemental figure 3) and prior evidence that they may be pathogenic (p.Arg117His^16^ and p.Asp224Asn^11^). In total, 14/43 variants were classified in this group, with 10 of these falling in the OB domains (figure 2; online supplemental tables 1 and 2). Group 2 variants were those predicted deleterious and probably damaging by both the SIFT and PolyPhen algorithms and did not disrupt POT1–ssDNA binding (4/43 variants). These variants may impair the function of the protein in other ways. The remaining variants (25) were classified into Group 3.

10.1136/jmg-2022-108776.supp3 Supplementary data

**Figure 2:**
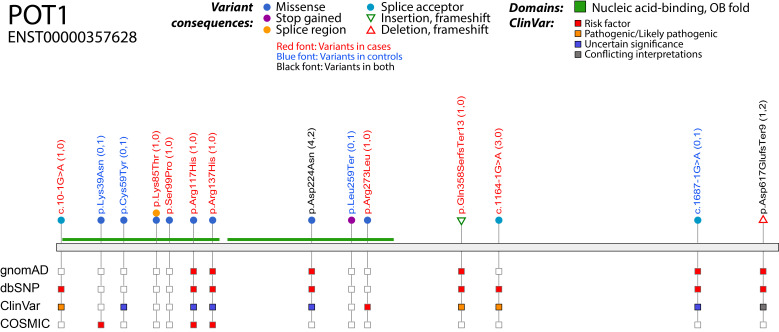
Schematic diagram of Group 1 POT1 variants. Variants are shown on the primary protein structure with their consequence (in a coloured circle or triangle) and their presence (red square) or absence (empty square) in publicly available datasets (gnomAD exomes v2.1, dbSNP build 151 and COSMIC v86). The ClinVar track indicates the pathogenicity prediction in ClinVar release 20220804. The OB domains are shown in green. Variants in red font colour are found in cases, those in blue font colour are found in controls and those in black are found in both cases and controls. For details on numbers of cases and controls, see online supplemental table 1. Figure created with VCF/Plotein.^22^

The majority of cases and controls in this study did not carry a *POT1* variant (94.1% cases, 95.1% controls), and the majority of those with a variant had only one variant. No person had more than two variants. In total, three persons had a Group 1 variant and a Group 3 variant (two cases, one control) while five persons had two Group 3 variants (three cases, two controls). Given the limited number of persons with two variants, each case and control is classified by their most severe mutation. For Group 1, 15 cases (0.51%) carried a variant, while 8 (0.24%) controls did (p value=0.08, OR for carrying a Group 1 variant compared with no variant (OR)=2.11, 95% CI (0.89 to 5.00)). For Groups 1+2 combined, 22 cases (0.75%) carried a variant, while 14 controls (0.42%) did (p value 0.096, OR=1.78). Finally, for Group 3, 126 cases (4.3%) carried a variant as did 149 controls (4.6%) (two-tailed Fisher’s exact test, p value 0.66) indicating no evidence of increased risk associated with this variant class. Overall, then while about twice as many cases as controls carried predicted pathogenic variants in *POT1*, this difference was not conventionally statistically significant likely because of limited power even with a study this size. There were also no differences in age of onset, sex, family history or site of presentation by pathogenicity group when compared with those without one of the classified mutations (online supplemental tables 3−6).

10.1136/jmg-2022-108776.supp4 Supplementary data

We next sought to determine whether the variants we detected had any effect on telomere regulation. For this, we measured telomere length in *POT1* variant carriers and non-carriers from the same populations (online supplemental methods). After standardising lengths by plate and adjusting them for cohort via a linear model (online supplemental table 7, online supplemental figure 4), we observed that only the individuals carrying the p.Lys39Asn (percentile 98 when compared with controls) and the p.Arg273Leu (percentile 99 when compared with controls) variants had telomeres that were substantially longer than the mean (figure 1B). We also observed that some individuals with splice variants or variants that showed reduced DNA binding also had telomeres on the longer side of the distribution (eg, Lys85Thr, percentile 91, p.Leu259Ter, percentile 90, one individual carrying c.1164–1G>A, percentile 97) but others did not (eg, p.Ser99Pro, percentile 31, most individuals with variants in splice sites). Individuals with the p.Asp224Asn variant had telomere lengths scattered throughout the whole distribution in contrast to previous reports suggesting that these variants increase telomere length^11^ (figure 1B).

10.1136/jmg-2022-108776.supp5 Supplementary data

Because the p.Lys39Asn, p.Cys59Tyr and p.Asp224Asn variants are found in controls and show POT1–ssDNA complex disruption, we further investigated those using molecular dynamics simulations (online supplemental methods). Our results suggest that all three variants affect the dynamics of the system when compared with the wild-type (WT) structure, as evidenced by the first and second normal modes (online supplemental figure 5A–H, online supplemental movie). Existing protein structures for POT1 also imply that there are conformational differences between the POT1–ssDNA and POT1–ACD structures.^17 18^ As a result, the structural differences noted within the POT1 mutant proteins investigated here may affect shelterin complex formation, but further investigation is necessary. Additional analyses of root mean square deviation, root mean square fluctuation, residue-wise correlations, secondary structure, energy decomposition analysis and hydrogen bond interactions are all consistent with the computational results reported herein (online supplemental figure 5I-L, 6–18, online supplemental tables 8-12). MM-GBSA was used to assess the protein:DNA-binding affinities. We calculated a ΔΔH of −0.6 to –1.3, and 21.6 kcal/mol for p.Lys39Asn, p.Asp224Asn and p.Cys59Tyr, respectively. These enthalpies are in agreement with the experimental binding pattern discussed above.

Even though *POT1* seems to be the second major melanoma susceptibility gene, with 2%–4% of *CDKN2A/CDK4*-WT families carrying a pathogenic coding variant in this gene, its contribution to melanoma risk burden in the general population is minor, with ~0.5% of cases carrying pathogenic variants. Telomere length calculations confirm known associations of variants with longer telomeres (p.Arg273Leu,^10^ p.Arg117His^11 16^) and found associations with other pathogenic variants (p.Lys39Asn, p.Lys85Thr and confirmation of longer telomere length for p.Ala532Pro, percentile 93, a variant originally reported in Ref. 11), but for other variants the association with length was not clear (eg, all three carriers of c.1164–1G>A and six of p.Asp224Asn had telomere lengths scattered throughout the distribution). Although a prior study had shown slightly longer telomeres for carriers of p.Arg117His,^11^ the carrier melanoma case in this cohort had normal-length telomeres. This may reflect the many mechanisms, including other genetic variants and lifestyle, by which telomere length can be affected or the assays used for telomere analysis. Telomere length for some control individuals (without reported melanoma) with pathogenic variants (eg, p.Lys39Asn and both controls carrying p.Asp224Asn) also showed an increase in telomere length, which may portend an increased risk of tumourigenesis in these individuals or indicate that other factors are necessary for melanoma genesis.

Although in this study we have attempted to identify pathogenic *POT1* variants through DNA-binding assays, the function of POT1 proteins with variants outside the OB domains may be compromised by other mechanisms. For example, another study concluded that the *POT1* p.Ala532Pro variant shows impaired ACD binding, which may also lead to telomere dysregulation.^19^ Therefore, further systematic experiments are needed to address other POT1 functions, such as telomere fragility, to provide a more complete catalogue of variants that alter protein function and therefore that lead to cancer predisposition.

While the number of *POT1* variant carriers in this study is too limited to draw strong conclusions, the lack of any statistically significant difference in age of onset between variant carriers (54.7 years) and non-carriers (54.4 years) in the general population needs some consideration. By comparison and looking at another melanoma high-penetrance gene, in the Leeds Melanoma Cohort, *CDKN2A* variant carriers have an average age of onset of 50 years (based on data included in Ref. 20). The literature contains many examples of families with particularly early ages of onset for melanoma; these extreme families likely represent the product of interactions of high penetrance variants (in genes such as *CDKN2A* and *POT1*) with contributing lower penetrance variants and risk-associated lifestyle behaviours. Therefore, the analysis of population-based samples provides a more complete description of the impact of high penetrance variants in the general population. A comparable scenario applies to breast cancer; recent analysis of the UK SEARCH study containing about 12 700 breast cancer diagnosed under the age of 70 years showed an average age of onset of 54.5 years for women without a known variant in a high penetrance gene. Only *BRCA1* and *BRCA2* variant carriers had notably earlier ages of onset (46.7 and 50.6 years, respectively), while carriers of variants in rarer predisposing genes (*CHEK2*, *PALB2*, *ATM*, *RAD51C*) had average age of onset of between 51.1 years and 58.2 years (A Antoniou, University of Cambridge, personal communication based on data in Ref. 21).
