# Supplementary material for: Population-based analysis of POT1 variants in a cutaneous melanoma case–control cohort
Source: J Med Genet. 2022 Dec 20;60(7):692–6. doi: 10.1136/jmg-2022-108776 (PMC10279804; doi:10.1136/jmg-2022-108776)
Supplement: Supplementary data [file jmg-2022-108776supp001.pdf]

Supplementary Materials

Materials and methods

Participants and sample collection. Samples used in this study came from three different cohorts.

(i) The Leeds Melanoma Case Control Study (LMC) recruited population-ascertained melanoma cases and controls. Cases came from Yorkshire and the North East of England, with recruitment starting in the year 2000 (NRES Committee North East - Northern and Yorkshire, MREC/01/3/057) [1] (**Supplementary Table 13**). Controls were recruited from the GPs of cases participating in the study and were recruited so that their age and sex distribution was similar to the distribution among cases. The controls and about half the cases were screened once at recruitment for skin lesions and were asked about family history. The remaining cases completed self-report questionnaires about family history.

(ii) Additionally, samples were included from the Study of Epidemiology and Risk Factors in Cancer Heredity (SEARCH) series of population-based studies initially in Eastern England but extended to Scotland (Cambridgeshire South Research Ethics Committee, 05/MRE05/1) [2] (**Supplementary Table 13**). Healthy control participants, *i.e*. people with no history of cancer to be used as comparisons with the cancer patients, were also recruited from 2003 to 2009 in East Anglia.

(iii) Finally, controls were supplemented with samples from the Wellcome Trust Case Control Consortium [3] (South East Multicentre Research Ethics Committee, 05/Q0106/74) (**Supplementary Table 13**). For the WTCCC controls, only basic information provided by the Wellcome Trust was available under the terms of the ethics agreement.

Sequencing and variant calling. DNA from whole blood was extracted for exon capture and sequencing. We used Fluidigm PCR-based amplicon sequencing to amplify all of the coding exons and splice junctions of the *POT1* gene in 7,024 samples and sequenced these products on the MiSeq platform by single-read sequencing. The reference sequence used was ENST00000357628. After aligning with BWA and filtering to keep only highly covered samples (Those with >94% of coding *POT1* exon bases covered >= 10 reads with MQ >= 50 and base quality >= 20), keeping only one sample out of each pair of relatives, removing samples with non-European ancestry and samples from patients that withdrew from the study, and removing related

controls, 6,226 samples remained. These samples included 2,928 cases (1,574 from the Leeds Case Control Consortium and 1,354 from the SEARCH Consortium) and 3,298 controls (1,431 from the WTCCC collection, 459 from the Leeds Case Control Consortium and 1,408 from the SEARCH Consortium) (**Supplementary Table 13**). We took forward for analysis the union of calls made by HaplotypeCaller (command line below in this section) and Samtools mpileup (parameters -t DP,SP -C50 -m2 -F0.0005 -d 10000 -ug), and performed quality variant filtering (mpileup: QUAL>=20 && (DP4[2]>30 || DP4[3]>30); GATK: QUAL>=20 && AD[0:1]>30). We also removed indel calls between GRCh37 coordinates 7:124475296-124475328, as this highly repetitive region (at an intron-exon boundary) seems to be germline microsatellite unstable and may be prone to false positive calls. We called 141 different protein-altering variants (missense, stops, frameshifts and splice acceptor/donor sites) in 3,546 samples electing to use a low stringency approach so as to capture all possible variants for validation. Validation by re-sequencing with Sanger or Illumina technologies was performed for at least one sample for all detected variants, often re-sequencing all carrier samples (**Supplementary Table 14**). Illumina sequencing was performed by exome capture with Agilent Technologies probes, using the WTSI v4 Solid Tumor Panel which included other established melanoma predisposition genes such as *CDKN2A*, *BAP1* and *CDK4*. Sequencing captured all exons and exon/intron boundaries of *POT1*, and succeeded for 164 samples. Overall, 158/164 samples were covered to an average depth higher than 10x across all *POT1* coding exons (**Supplementary Table 15**). Variants were called from these data using Samtools mpileup in pooled mode. By applying this approach, we validated 40 unique variants originally identified using the Fluidigm PCR-based amplicon but importantly identified no new variant positions by this method. Capillary resequencing of variants found in the 19 samples that failed library preparation or were included in a later sequencing effort confirmed three additional variants, for a total of 43/141 protein-altering variants confirmed (**Supplementary Tables 1,2, 16**). A simplified flowchart with these analysis steps can be found in **Supplementary Figure 19**. No additional variants were called in the resequenced samples. Consequences were predicted with the Variant Effect Predictor (VEP), from Ensembl release 104, using the web tool with the GRCh37 human reference genome. Since our exon capture sequenced additional known melanoma driver genes (above) we screened all samples found to carry mutations in *POT1* to exclude the possibility that they also carried pathogenic variants in drivers *CDK4*, *CDKN2A* and *BAP1*. Three protein-coding variants were found in *CDKN2A* in *POT1* variant carriers, although these *POT1* variants were classified as benign according to our G-strand binding assays (**Supplementary Table 17**, reference transcripts *CDKN2A*: ENST00000304494, *CDK4*: ENST00000257904, *BAP1*: ENST00000460680). For the pathogenicity group classification,

whenever there were two *POT1* variants found in the same patient, we classified the patient in the higher pathogenicity group. No participant had more than one Group 1 or 2 variants.

***In vitro* translation and G strand binding assays**. pEX-POT1 plasmid vectors, harboring wild-type or mutant POT1 ORF sequences downstream of a T7 promoter, were used for in vitro translation reactions with TNT coupled reticulocyte lysate kit (Promega) according to the manufacturer's instructions. Protein expression was verified by immunoblotting an aliquot of each reaction with anti-POT1 antibodies (Abcam, ab124784). A telomeric oligonucleotide probe (GGTTAGGGTTAGGGTTAGGG) was end-labelled using [-32P] ATP (Perkin Elmer) with T4 polynucleotide kinase (New England Biolabs). Unincorporated nucleotides were removed using illustra MicroSpin G-25 columns (GE Healthcare) according to the manufacturer's instructions. DNA-binding assays were performed by mixing 5μl translation reaction in 20 μl final volume containing binding buffer (25 mM HEPES-NaOH [pH 7.5], 100 mM NaCl, 1 mM EDTA and 5% glycerol), 1 μg poly (dI-dC) (Thermo) and 10 nM [32P]-labelled telomeric oligonucleotide probe for 10 min at room temperature. Reactions were separated on 6% DNA retardation gels (Novex) in 0.5 TBE buffer at 80 V. Gels were dried and exposed to Hyperfilm MP film (Amersham), which was developed using a Compact X4 machine (Xograph).

Analysis of telomere length by telomere repeat PCR. We measured telomere length in all Illumina re-sequenced cases and controls from the Leeds, SEARCH and WTCC cohorts who carried a potential *POT1* variant according to the initial Fluidigm analysis, as well as age and sex-matched controls (A total of 174 samples (of which 66 belong to the Leeds cohort, 86 to SEARCH and 22 to WTCC; 105 are melanoma cases and 69 are non-melanoma controls, and 48 pathogenic POT1 variant carriers and 126 non-carriers)). Telomere length was quantified by real-time PCR using the 'Absolute Human Telomere Length Quantification qPCR Assay Kit (AHTLQ)' (ScienCell Research Laboratories, CA, USA) according to the manufacturer's instructions. Each DNA sample was amplified in two separate reactions: using the telomere (TEL) primer set; and the single copy reference (SCR) primer set. The telomere primer set recognises and amplifies telomere sequences. The SCR primer set amplifies a 100bp region on chromosome 17, and acts as a reference for normalisation.

Reactions were carried out in 20 μL volume: 1 μL DNA (5ng); 2 μL primer (TEL or SCR); 10 μL 2×GoldNStart TaqGreen qPCR master mix; and 7 μL nuclease-free water. A QuantStudio 5 Real Time PCR machine (Life Technologies, CA, USA) was used for qPCR, using a 96-well plate

format. The PCR conditions were: initial denaturation at 95°C for 10 minutes, then 32 cycles of 95°C for 20 seconds, 52°C for 20 seconds and 72°C for 45 seconds. All reactions were performed in triplicate, and the same reference genomic DNA sample was included in each run. Data were analysed using the QuantStudio Design and Analysis Software version 1.4.1. (Life Technologies, CA, USA), and absolute telomere length was calculated by reference to the DNA standard using comparative ddCq according to the AHTLQ kit instructions.

A linear model adjusting for age at diagnosis, sex and cohort was done with the individuals that did not have pathogenic variants (pathogenic variants were defined as all detected variants except for p.Ile22Val, p.Gln301His, p.Gln376Arg, and p.Gly404Val, which all have a gnomAD overall allele frequency higher than 1 x 10^{-4}), whether melanoma cases or controls. The linear model showed that neither age nor sex where significantly related to the telomere length in our data. There is probably too much noise introduced by cohort origin, so we opted for using only cohort to control our data. The residuals of this linear model were used to create a telomere length distribution for this cohort. Telomere length adjustment for pathogenic variant carrier individuals was done separately with the same parameters calculated from the population distribution.

Molecular dynamics simulations of WT and variant POT1-ssDNA. Molecular dynamics (MD) simulations were performed using AMBER20's pmemd.cuda with the ff19SB force field for protein and OL15 for DNA[4–7]. The model was constructed from PDB ID 1XJV,[8, 9] using *Coot* and MolProbity to alleviate bad clashes[10, 11]. We used the Modeller interface in UCSF Chimera to incorporate missing residues,[12–14] as well as Chimera's integrated Dunbrack rotamer library to create the p.Lys39Asn, p.Cys59Tys, and p.Asp224Asn variants[15]. The systems were solvated with TIP3P water in a cube that extended 12 Å from the complex surface, and potassium was added to neutralize the charge of the system[16]. All MD simulations were run in triplicate for 250 ns while holding the number of atoms, pressure, and temperature constant (NPT ensemble) with the Langevin thermostat and barostat. A 9 Å cutoff was used for long-range non-bonded interactions with the smooth particle mesh Ewald method for electrostatics[17]. AMBER’s cpptraj program was used for root mean square deviation (RMSD), root mean square fluctuation (RMSF), hydrogen bond interactions, and secondary structure analyses[18]. Both cpptraj and the ProDy module in VMD were used for normal mode analysis[19, 20]. A Fortran90 program developed by the Cisneros group was used to perform an energy decomposition analysis (EDA) on each simulation[21]. The data.table, tidyverse, and abind packages of R were used to analyze the EDA data and hydrogen bond interactions[22–25]. The MM-GBSA method, implemented through

MMPBSA.py in AmberTools, was used to calculate the binding enthalpies between the protein and ssDNA. [26–28] VMD, UCSF Chimera, gnuplot, matplotlib, and ggplot2 were used for data visualization and image creation[14, 20, 29–31].

Command lines used. GATK HaplotypeCaller command line:

analysis_type=HaplotypeCaller input_file=[example.bam] showFullBamList=false read_buffer_size=null phone_home=NO_ET gatk_key=gatk.key tag=NA read_filter=[] disable_read_filter=[] intervals=[pot1.bed, 7:1-159138663] excludeIntervals=null interval_set_rule=INTERSECTION interval_merging=ALL interval_padding=0 reference_
sequence=hs37d5.fa nonDeterministicRandomSeed=false disableDithering=false maxRunti
me=-1 maxRuntimeUnits=MINUTES downsampling_type=BY_SAMPLE downsample_to_fraction=null
downsample_to_coverage=500 baq=OFF baqGapOpenPena
lty=40.0 refactor_NDN_cigar_string=false fix_misencoded_quality_scores=false allow_potentially_misencoded_quality_scores=false useOrigi
nalQualities=false defaultBaseQualities=-1 performanceLog=null BQSR=null quantize_quals=0 disable_indel_quals=false emit_original_quals=false preserve_qscores_less_than=6 globalQScorePrior=-1.0 validation_strictness=SILENT remove_program_records=false keep_program_records=false sample_rename_mapping_file=null unsafe=null disable_auto_index_creation_and_locking_when_reading_rods=false no_cmdline_in_header=false sites_only=false never_trim_vcf_format_field=false bcf=false bam_compression=null simplifyBAM=false disable_bam_indexing=false generate_md5=false num_threads=1 num_cpu_threads_per_data_thread=1 num_io_threads=0 monitorThreadEfficiency=false num_bam_file_handles=null read_group_black_list=null pedigree=[] pedigreeString=[] pedigreeValidationType=STRICT allow_intervals_with_unindexed_bam=false generateShadowBCF=false variant_index_type=DYNAMIC_SEEK variant_index_parameter=-1 logging_level=ERROR log_to_file=null help=false version=false out=/example_path/1_gatk_haplotype_caller_with_genome_chunking/7_1-159138663.gatk_haplotype.vcf.gz likelihoodCalculationEngine=PairHMM heterogeneousKmerSizeResolution=COMBO_MIN dbsnp=(RodBinding name= source=UNBOUND) dontTrimActiveRegions=false maxDiscARExtension=25 maxGGAARExtension=300 paddingAroundIndels=150 paddingAroundSNPs=20 comp=[] annotation=[ClippingRankSumTest, DepthPerSampleHC] excludeAnnotation=[] debug=false useFilteredReadsForAnnotations=false emitRefConfidence=NONE bamOutput=null bamWriterType=CALLED_HAPLOTYPES disableOptimizations=false annotateNDA=false heterozygosity=0.001 indel_heterozygosity=1.25E-4 standard_min_confidence_threshold_for_calling=4.0 standard_min_confidence_threshold_for_emitting=4.0 max_alternate_alleles=6 input_prior=[] sample_ploidy=2 genotyping_mode=DISCOVERY alleles=(RodBinding name= source=UNBOUND) contamination_fraction_to_filter=0.0 contamination_fraction_per_sample_file=null p_nonref_model=null exactcallslog=null output_mode=EMIT_VARIANTS_ONLY allSitePLs=false gcpHMM=10 pair_hmm_implementation=VECTOR_LOGLESS_CACHING pair_hmm_sub_implementation=ENABLE_ALL always_load_vector_logless_PairHMM_lib=false phredScaledGlobalReadMismappingRate=45 noFpga=false sample_name=null kmerSize=[10, 25] dontIncreaseKmerSizesForCycles=false allowNonUniqueKmersInRef=false numPruningSamples=1 recoverDanglingHeads=false doNotRecoverDanglingBranches=false minDanglingBranchLength=4 consensus=false maxNumHaplotypesInPopulation=128 errorCorrectKmers=false minPruning=2 debugGraphTransformations=false allowCyclesInKmerGraphToGeneratePaths=false graphOutput=null kmerLengthForReadErrorCorrection=25 minObservationsForKmerToBeSolid=20 GVCFGQBands=[1, 2, 3, 4, 5, 6, 7, 8, 9, 10, 11, 12, 13, 14, 15, 16, 17, 18, 19, 20, 21, 22, 23, 24, 25, 26, 27, 28, 29, 30, 31, 32, 33, 34, 35, 36, 37, 38, 39, 40, 41, 42, 43, 44, 45, 46, 47, 48, 49, 50, 51, 52, 53, 54, 55, 56, 57, 58, 59, 60, 70, 80, 90, 99] indelSizeToEliminateInRefModel=10 min_base_quality_score=10 includeUmappedReads=false useAllelesTrigger=false doNotRunPhysicalPhasing=true keepRG=null justDetermineActiveRegions=false dontGenotype=false dontUseSoftClippedBases=false captureAssemblyFailureBAM=false errorCorrectReads=false pcr_indel_model=CONSERVATIVE maxReadsInRegionPerSample=10000 minReadsPerAlignmentStart=10 mergeVariantsViaLD=false activityProfileOut=null activeRegionOut=null activeRegionIn=null activeRegionExtension=null forceActive=false activeRegionMaxSize=null bandPassSigma=null maxProbPropagationDistance=50 activeProbabilityThreshold=0.002 min_mapping_quality_score=20 filter_reads_with_N_cigar=false filter_mismatching_base_and_quals=false filter_bases_not_stored=false

VEP command:

./vep --af --af_
 --appris --biotype --buffer_size 500 --check_existing --distance 5000 --mane --polyphen b --pubmed --regulatory --sift b --species homo_sapiens --symbol --transcript_version --tsl --cache --input_file [input_data] --output_file [output_file] --port 3337

Supplementary Figures and legends

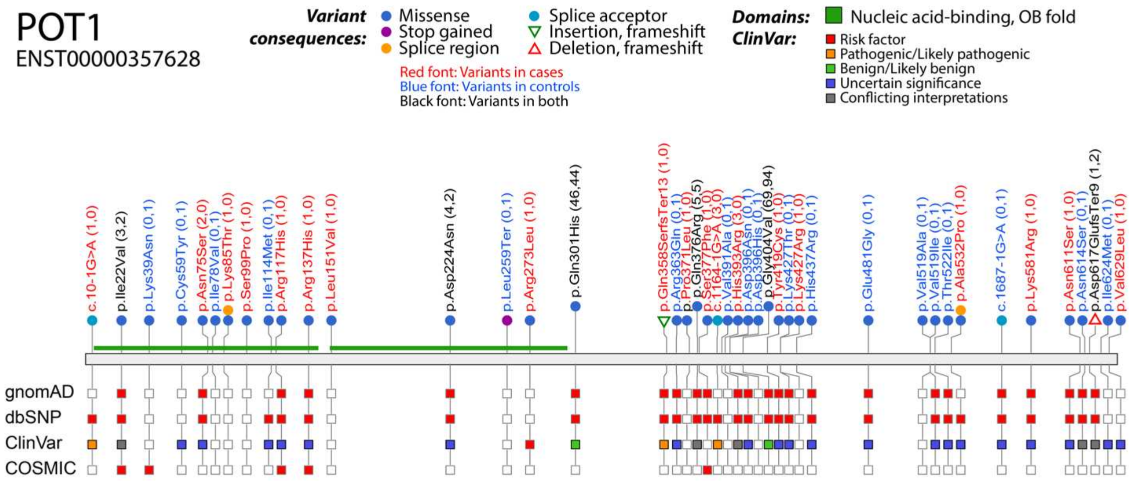

Supplementary Figure 1. Schematic diagram of POT1 variants identified in this case-control cohort study. Variants are shown on the primary protein structure with their consequence (in a colored circle or triangle) and their presence (red square) or absence (empty square) in publicly available datasets (gnomAD exomes v2.1, dbSNP build 151 and COSMIC v86). The ClinVar track indicates the pathogenicity prediction in ClinVar release 20220804. The OB domains are shown in green. Variants in red font colour are found in cases, those in blue font colour are found in controls and those in black are found in both cases and controls. For details on numbers of cases and controls see **Supplementary Table 1**. Figure created with VCF/Plotein [12].

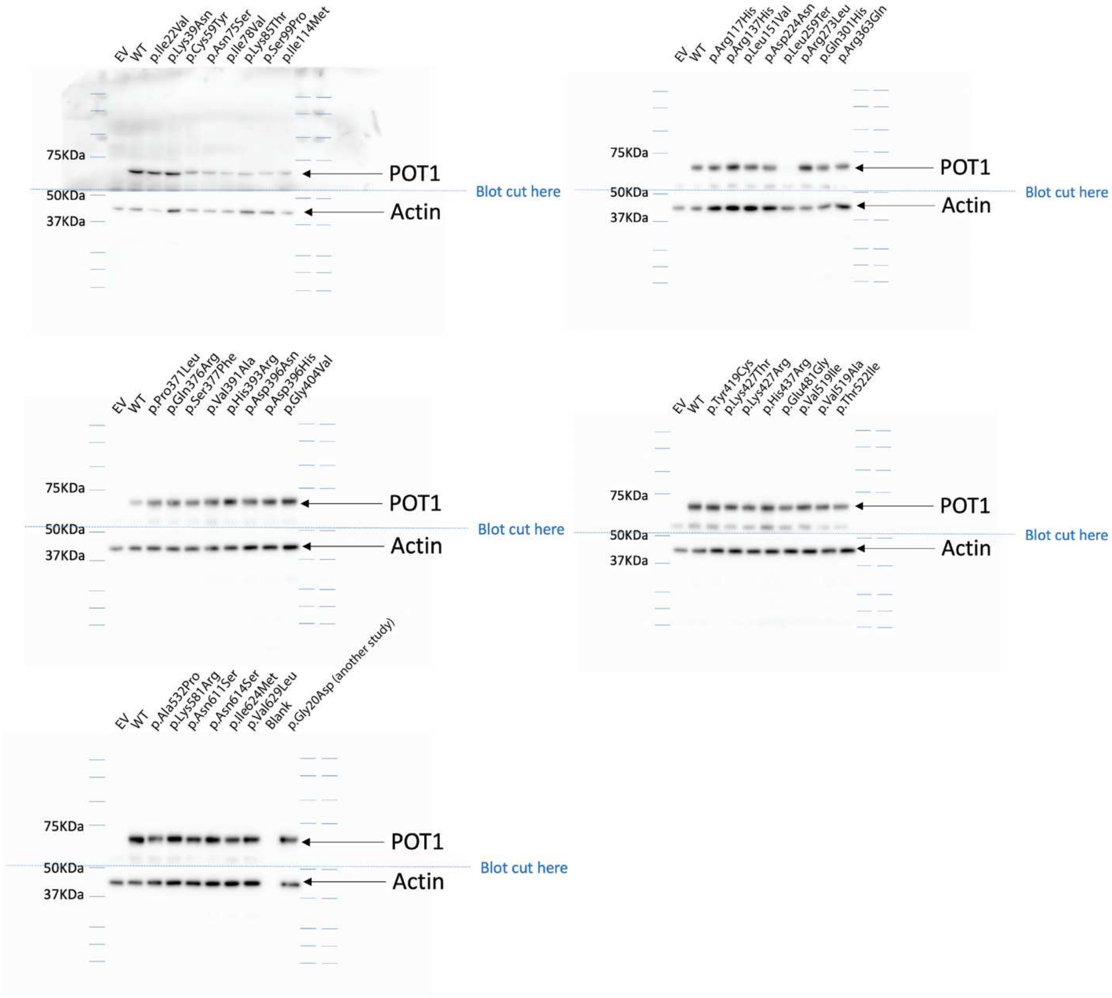

Supplementary Figure 2. Expression control for the POT1-ssDNA binding assays. The POT1 protein is shown alongside the expression of actin in each reticulocyte lysate reaction. The gels are shown in the same order as those in Figure 2A.

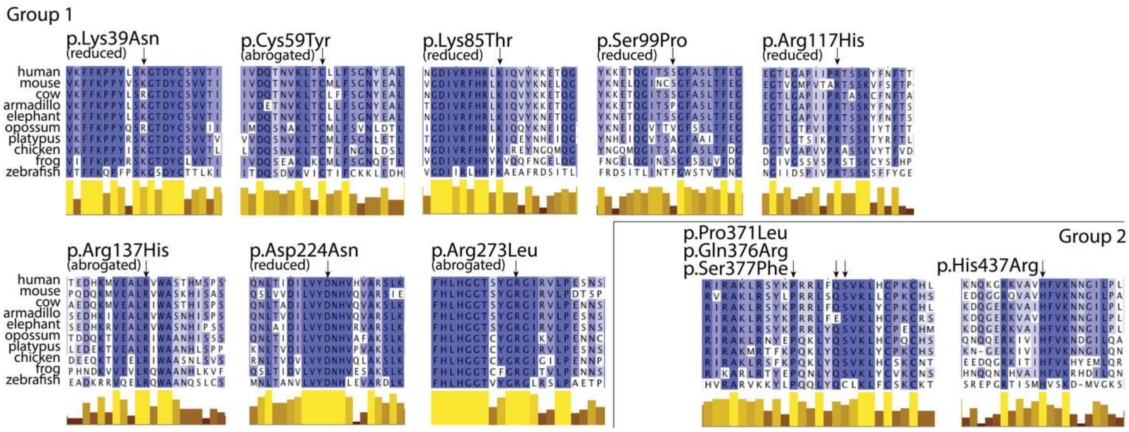

Supplementary Figure 3. Multi-species alignment of the POT1 protein sequence for missense variants in Pathogenicity Groups 1 and 2. A darker shade in an amino acid indicates higher sequence conservation across species. At the bottom, the height and color of the bars indicate sequence conservation level (taller and lighter bars indicate higher conserved residues). Protein sequences were downloaded from NCBI and are: NP_056265.2 (human), NP_598692.1 (mouse), DAA30462.1 (cow), XP_004478310.1 (armadillo), XP_003407293.1 (elephant), XP_007504312.1 (opossum), XP_001508179.2 (platypus), NP_996875.1 (chicken), AAI71328.1 (frog) and ADY16707.1 (zebrafish). Alignments were done with CLUSTAL O v 1.2.4 and rendered with Jalview.

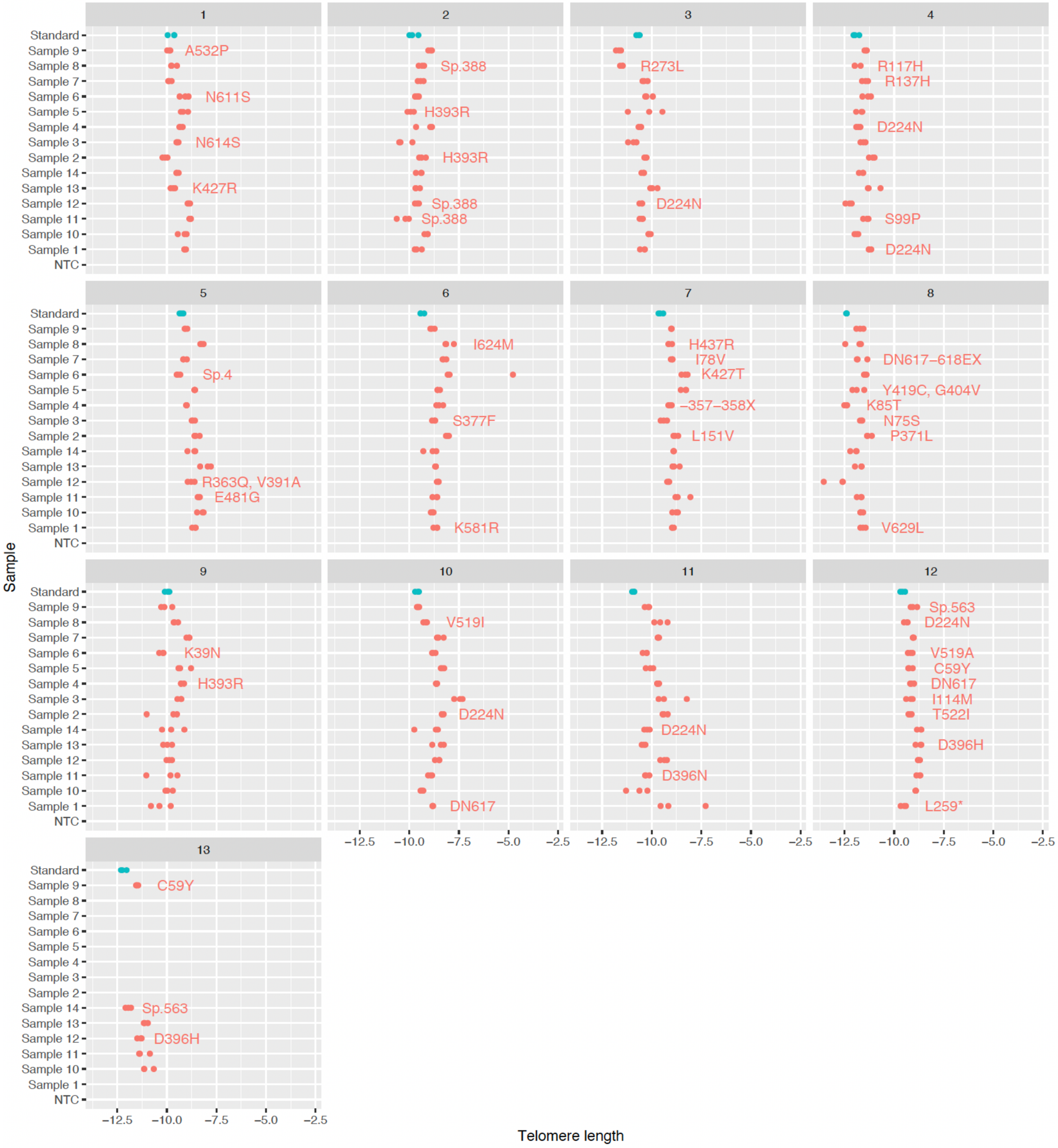

Supplementary Figure 4. Raw telomere length measurements. Each plate is shown, with the standard of known telomere length shown in the top row. Three replicates per sample were measured and are shown, the mean of measurements was used for the linear model and the distribution seen in Figure 2B. Variants carried by each sample are depicted.

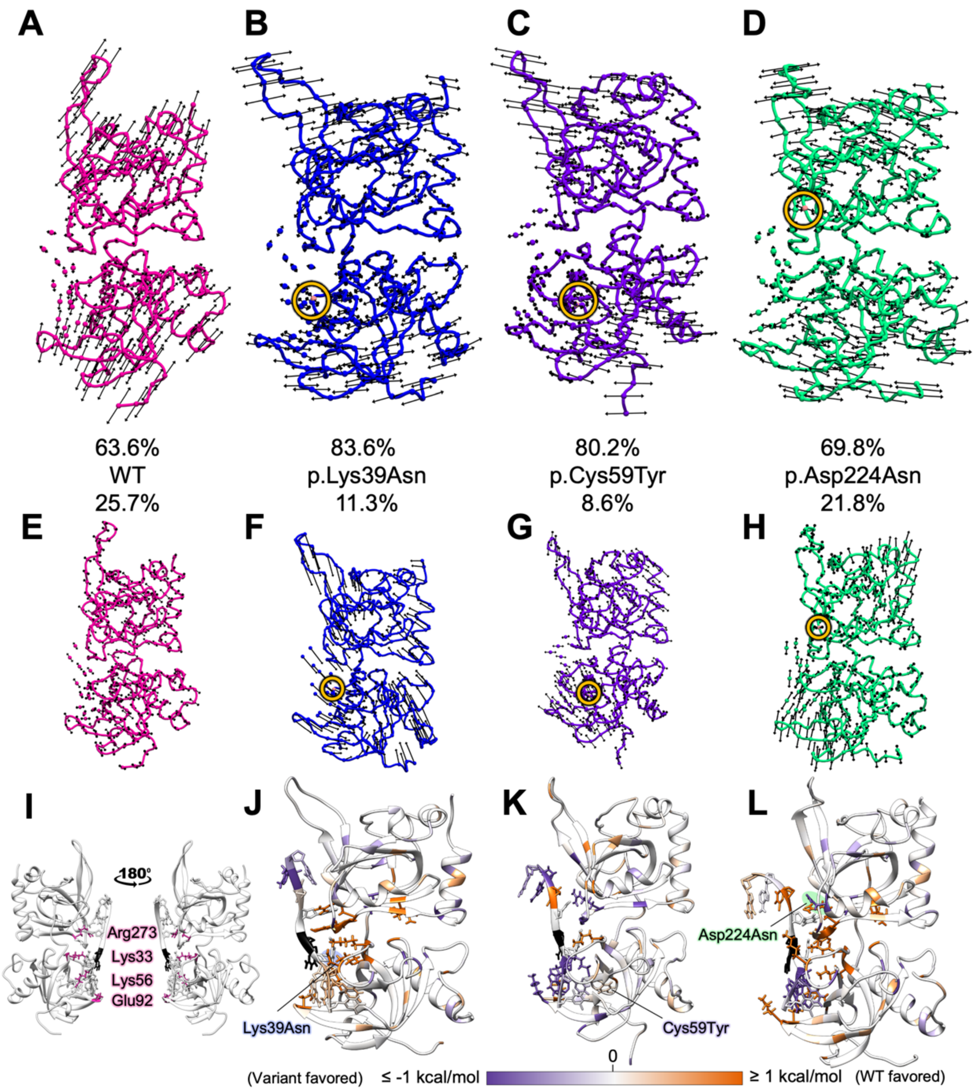

Supplementary Figure 5. **Variant differences in dynamics and non-bonded interactions.** (A–D) First and (E–H) second normal modes for (A,E) WT (B, F) K39N (C, G) C59Y and (D, H) D224N. The mutation position is colored pink and circled in yellow. (I) EDA results show that K33, K56, E92, and R73 (pink) have different non-bonded interactions with dG6 (black) across all variants when compared to WT. (J–L) Differences in the total interaction energy (Coulomb and van der Waals) with respect to the dG6 position for (J) K39N – WT, (K) C59Y – WT, and (L) D224N – WT. Interactions favored in WT are colored orange, those favored in the variant are colored, purple, and the dG6 position is colored black. All data are mapped onto the respective variant structure.

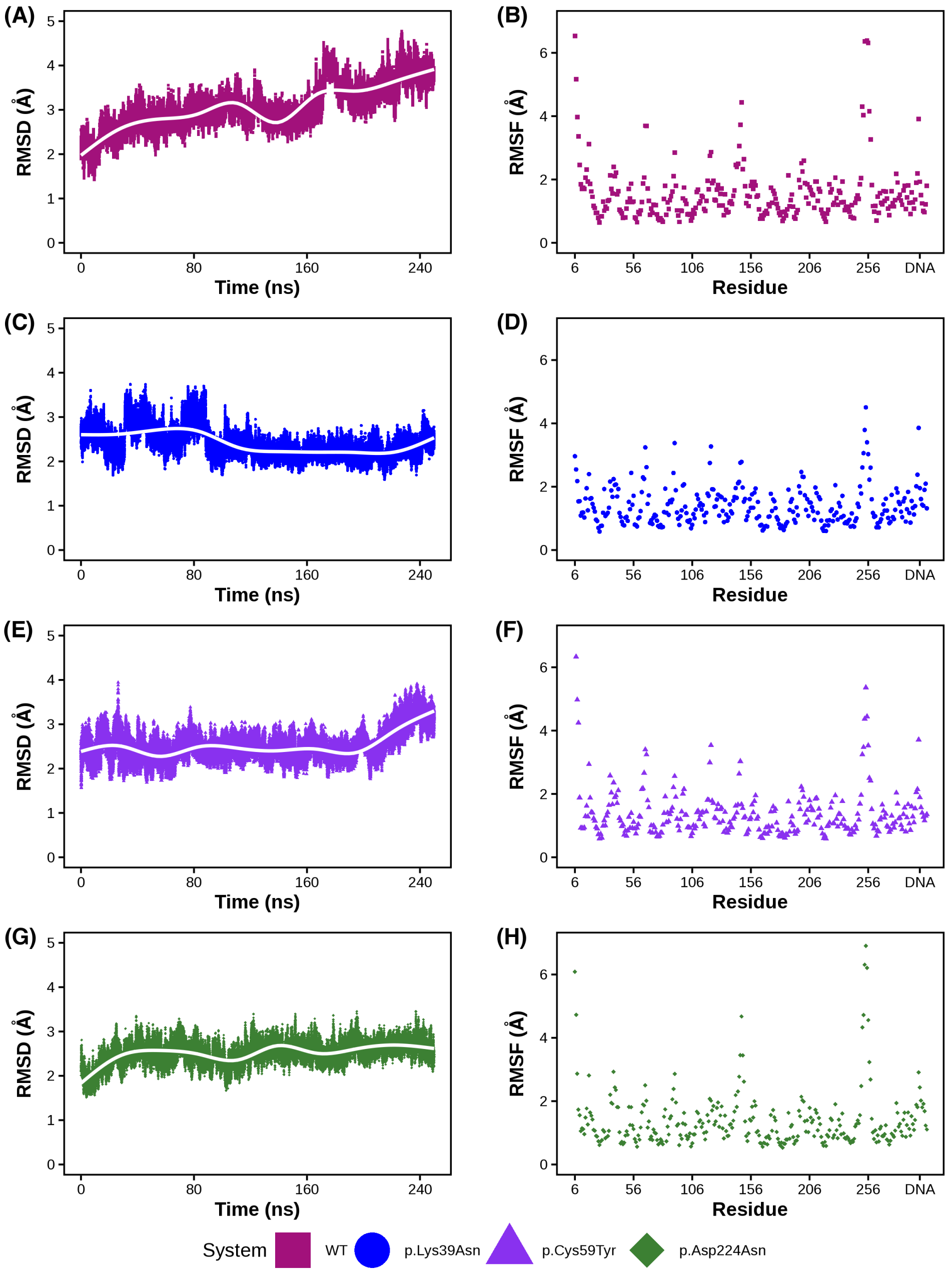

Supplementary Figure 6. RMSD and RMSF. RMSD (left) and RMSF (right) of the first MD replicate.

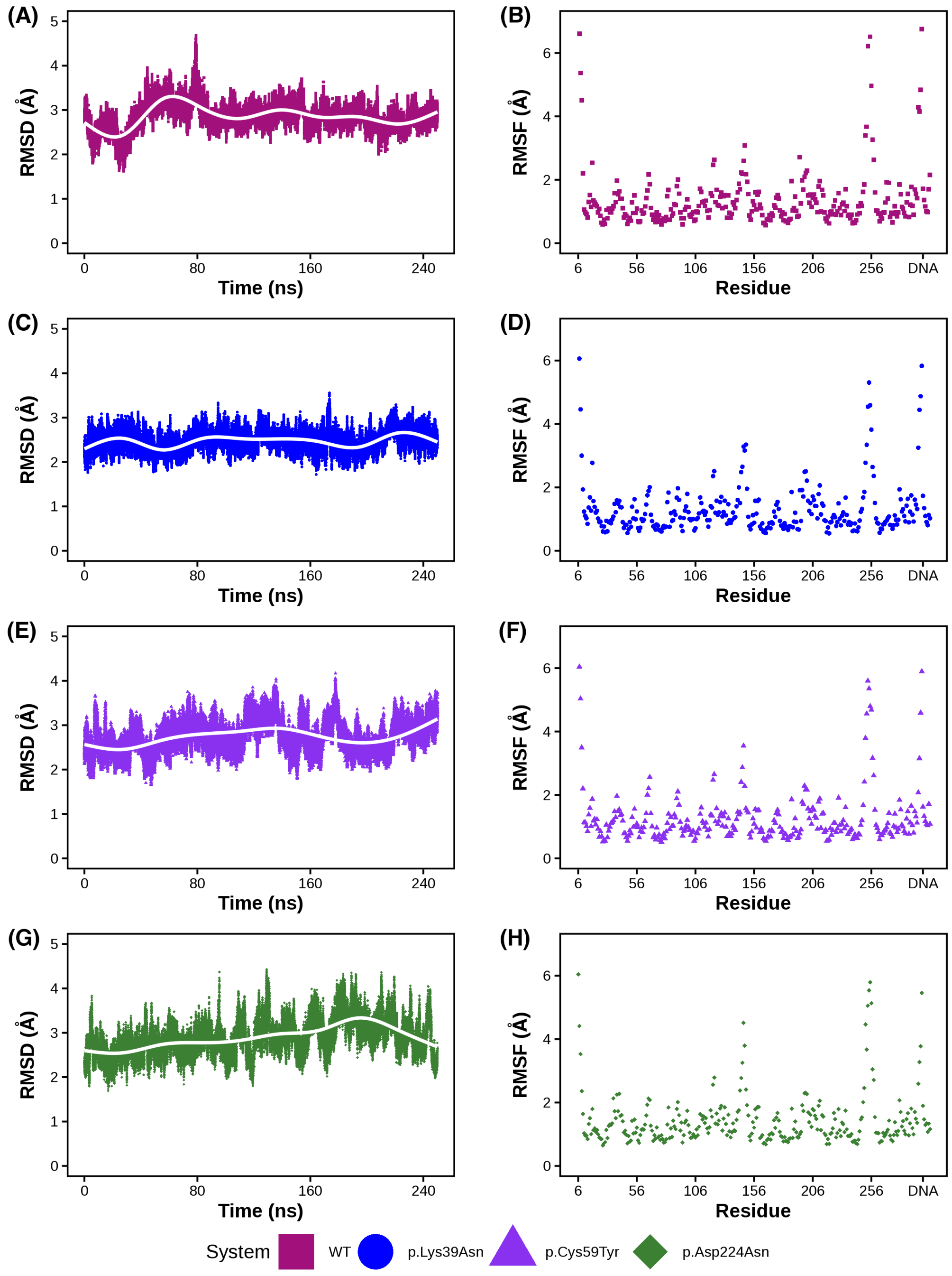

Supplementary Figure 7. RMSD and RMSF. RMSD (left) and RMSF (right) of the second MD replicate.

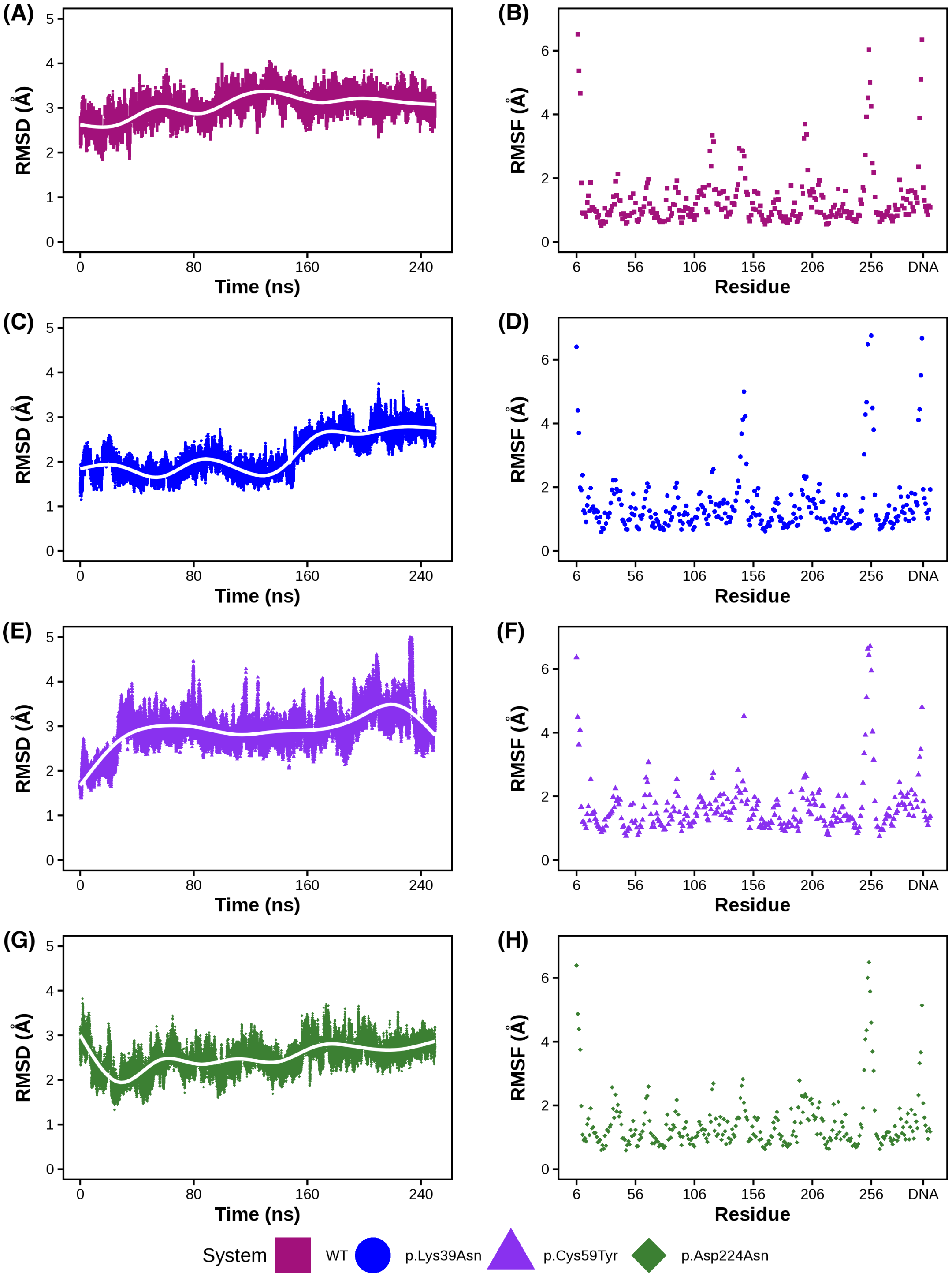

Supplementary Figure 8. RMSD and RMSF. RMSD (left) and RMSF (right) of the third MD replicate.

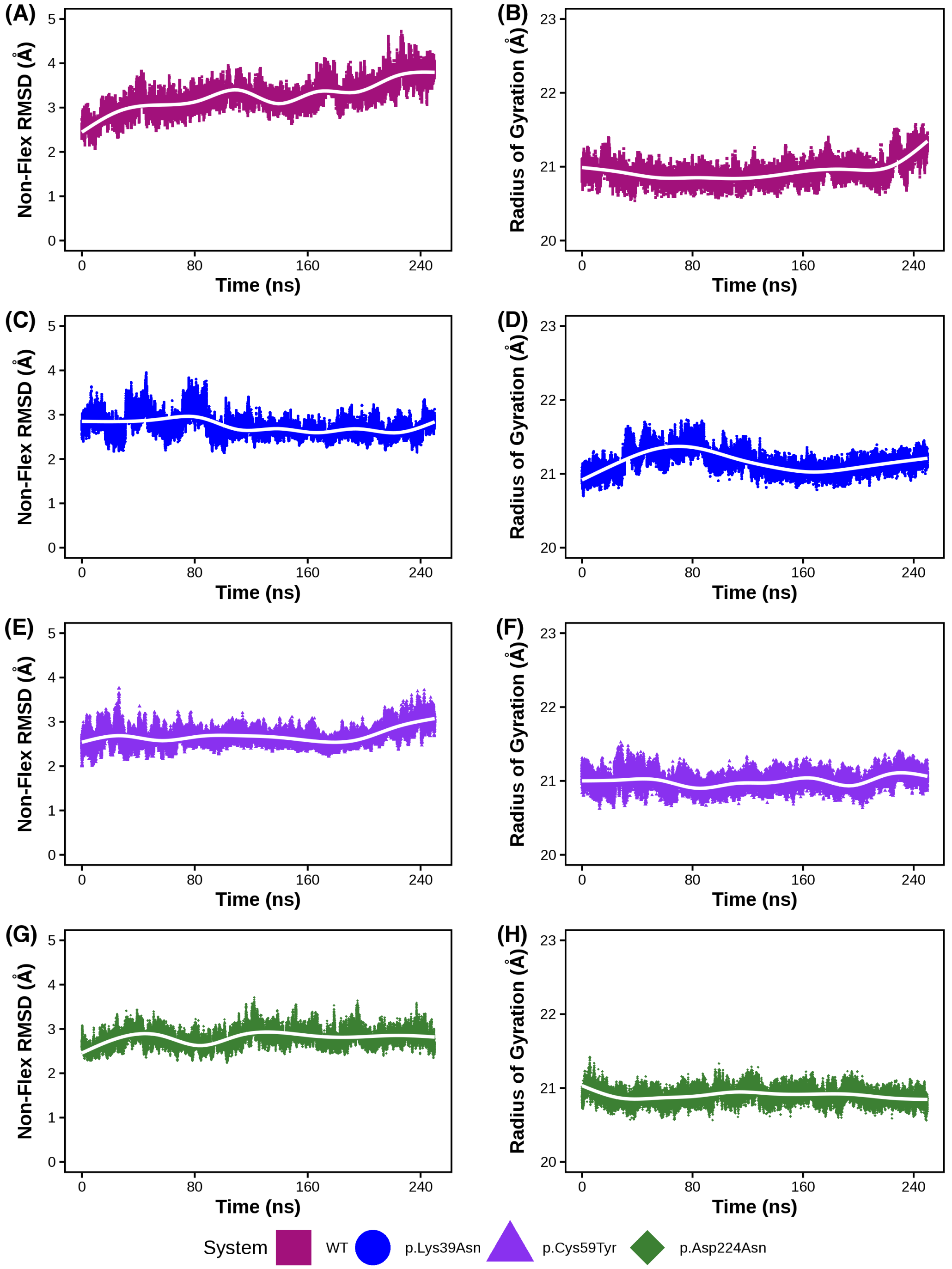

Supplementary Figure 9. Non-flexible RMSD and radius of gyration. Non-flexible RMSD (left) and radius of gyration (right) of the first MD replicate.

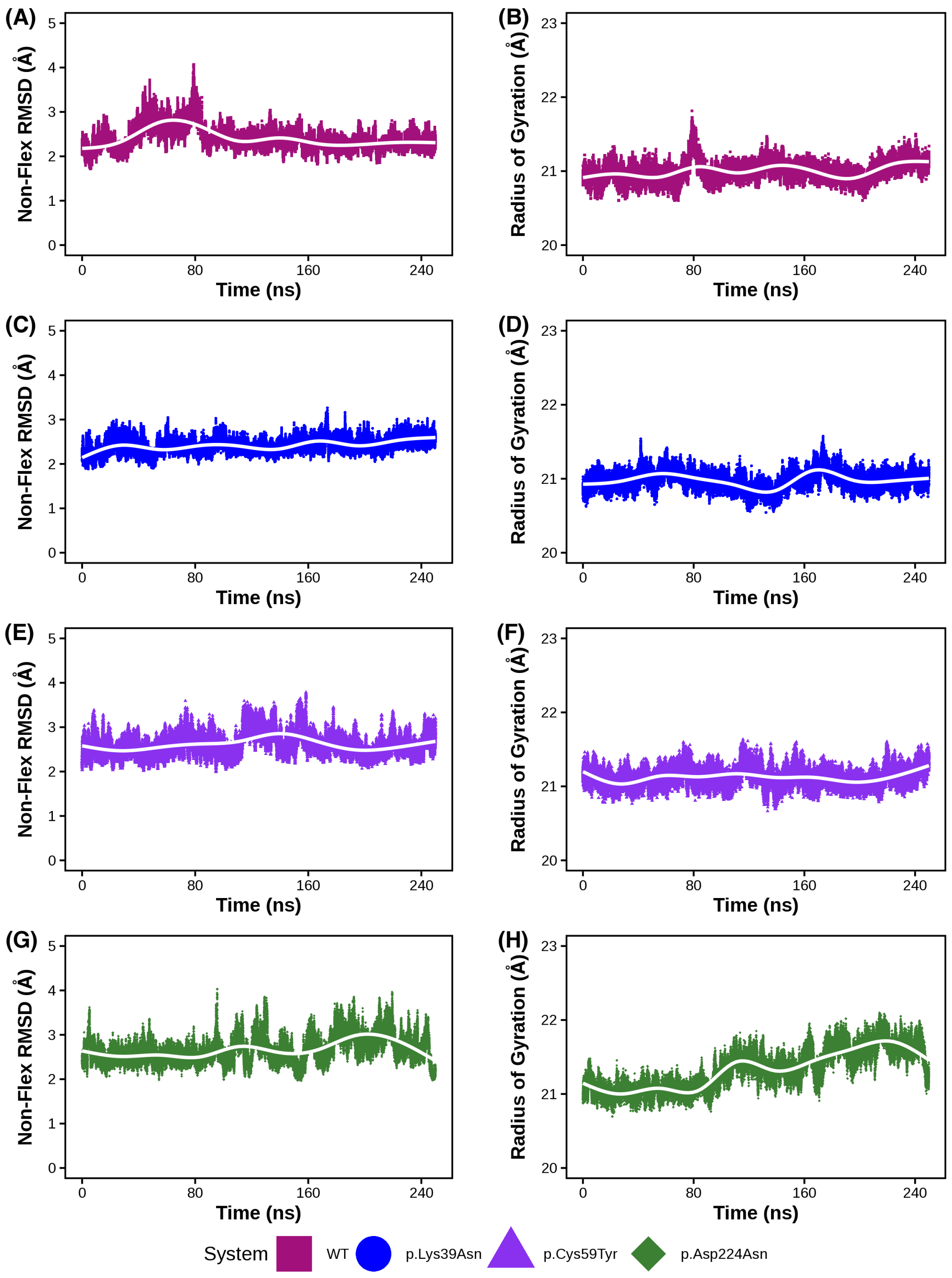

Supplementary Figure 10. Non-flexible RMSD and radius of gyration. Non-flexible RMSD (left) and radius of gyration (right) of the second MD replicate.

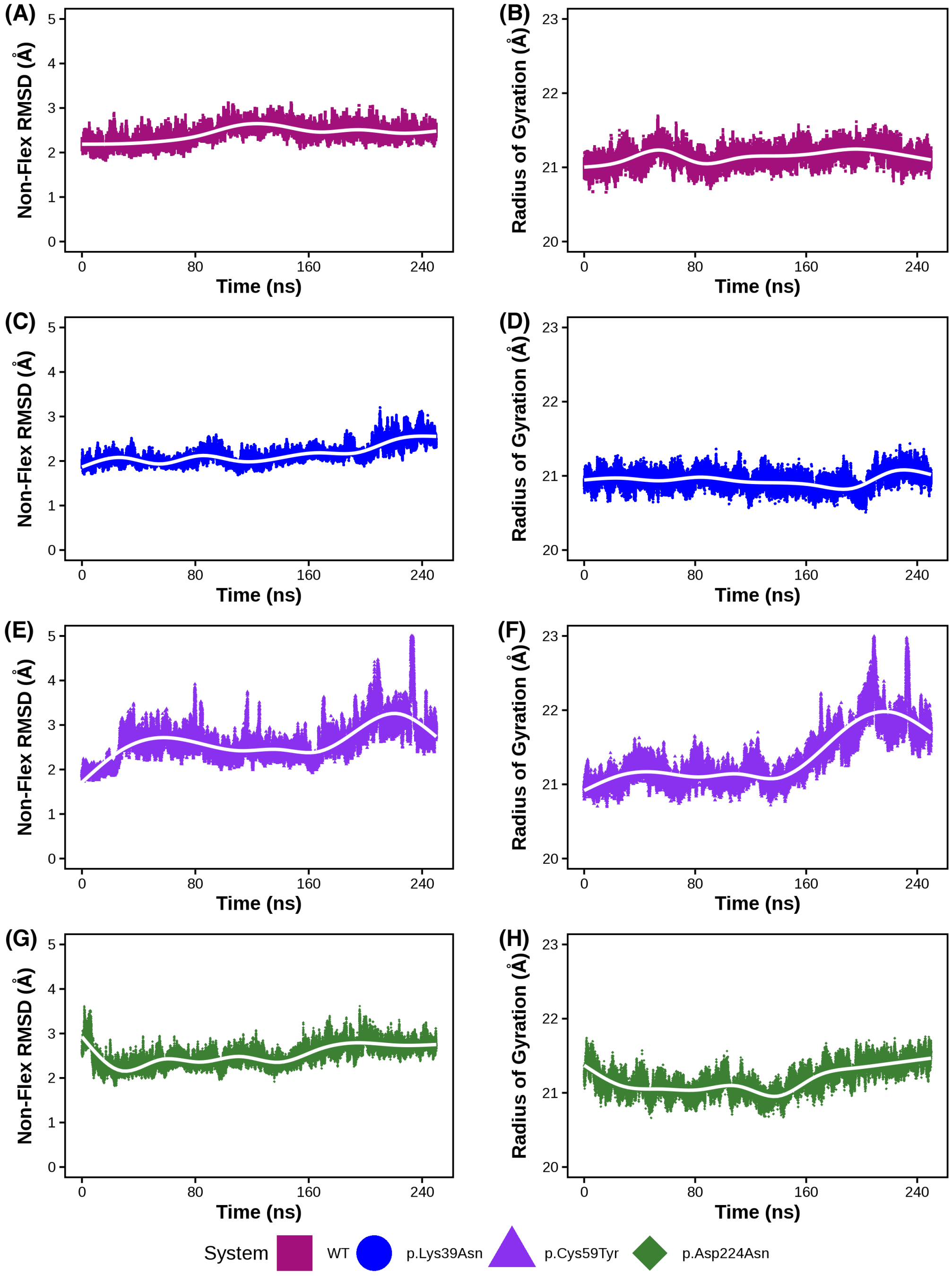

Supplementary Figure 11. Non-flexible RMSD and radius of gyration. Non-flexible RMSD (left) and radius of gyration (right) of the third MD replicate.

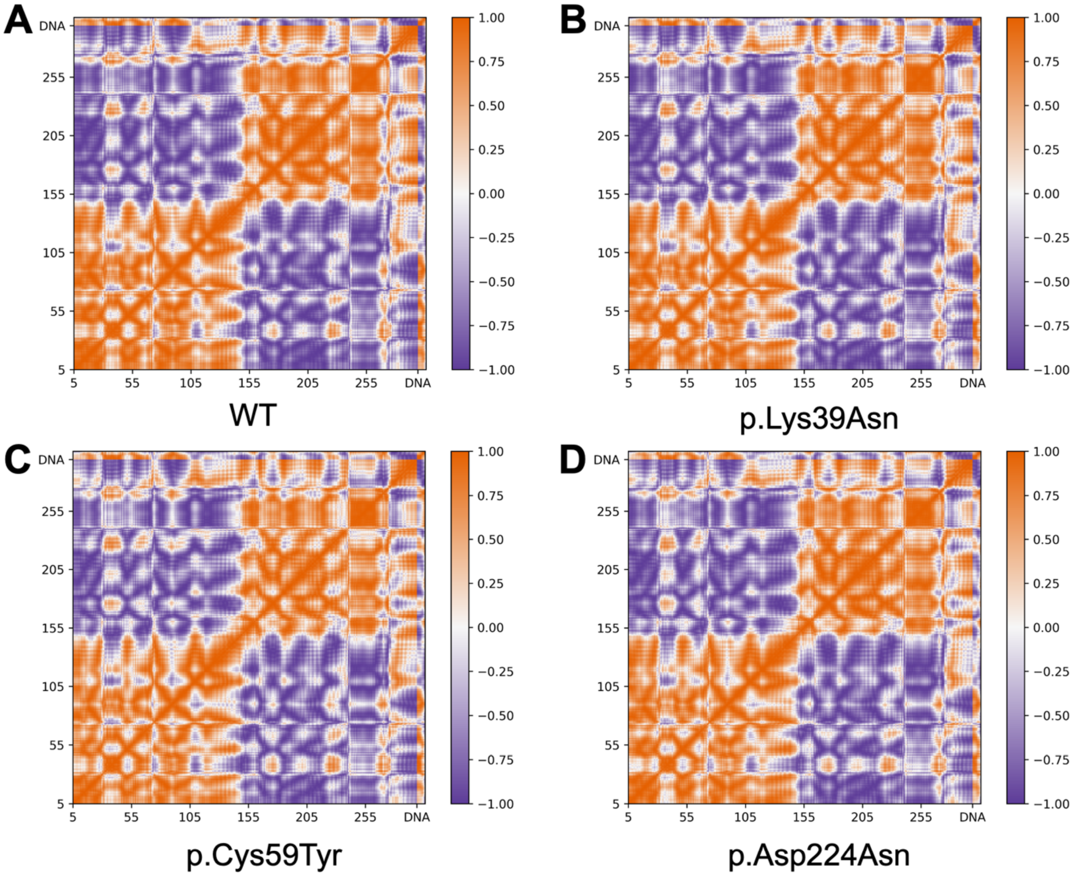

Supplementary Figure 12. Correlation matrices. Correlation matrices for (A) WT, (B) K39N, (C) C59Y, and (D) D224N. Areas of correlation are orange (1.0), areas with no correlation are white (0.0), and areas with anti-correlation are purple (-1.0).

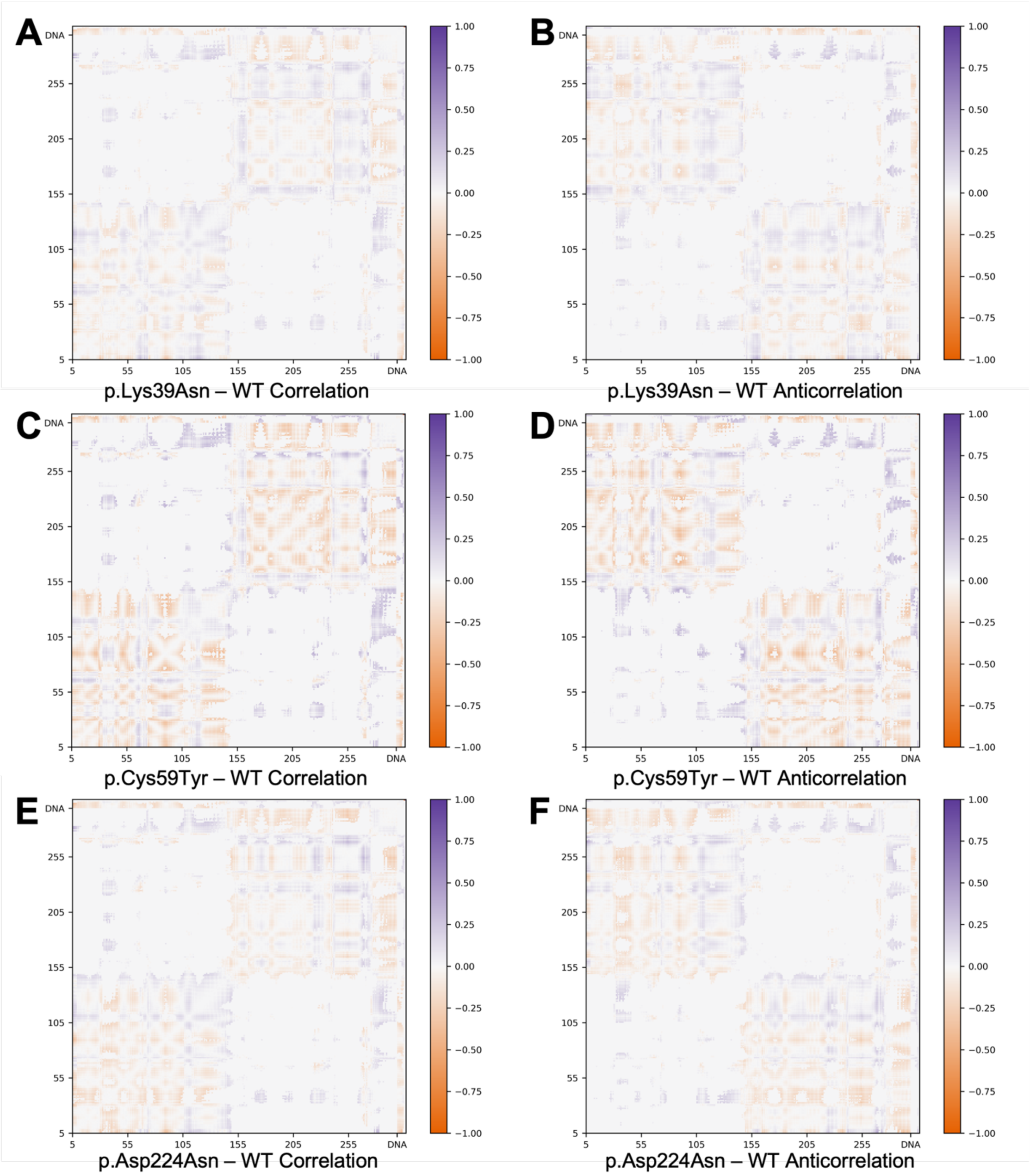

Supplementary Figure 13. Differences in correlated and anti-correlated motions. Differences in (A,C,E) correlated motions and (B,D,F) anticorrelated motions for (A–B) K39N – WT, (C–D) C59Y – WT, and (E–F) D224N – WT. Areas where that type of motion is more present in WT are orange, and areas where that type of motion is more present in the variant are purple.

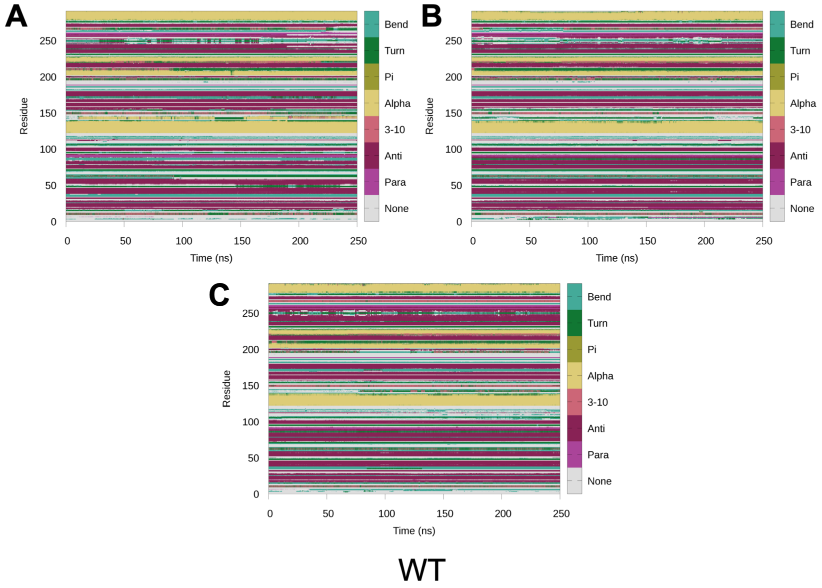

Supplementary Figure 14. WT secondary structure. Secondary structure analysis of each replicate of WT.

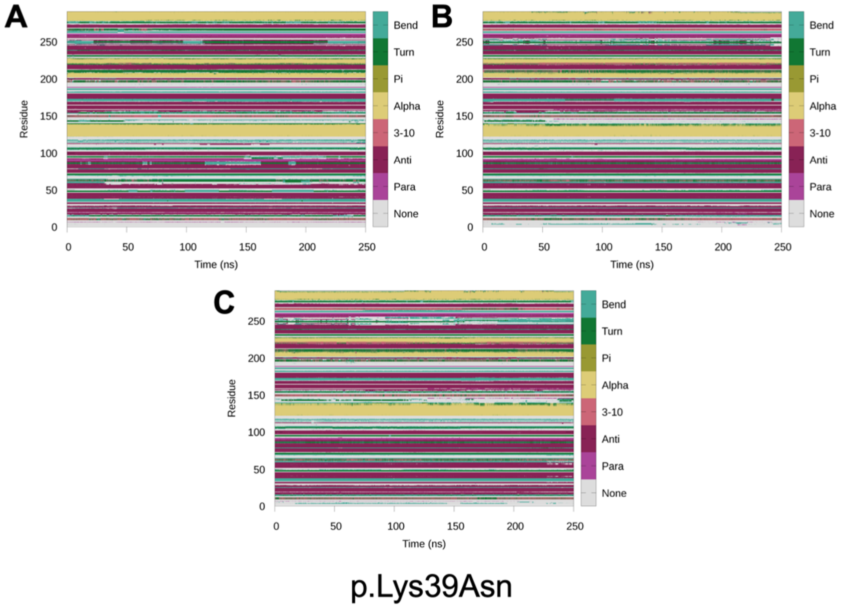

Supplementary Figure 15. K39N secondary structure. Secondary structure analysis of each replicate of K39N.

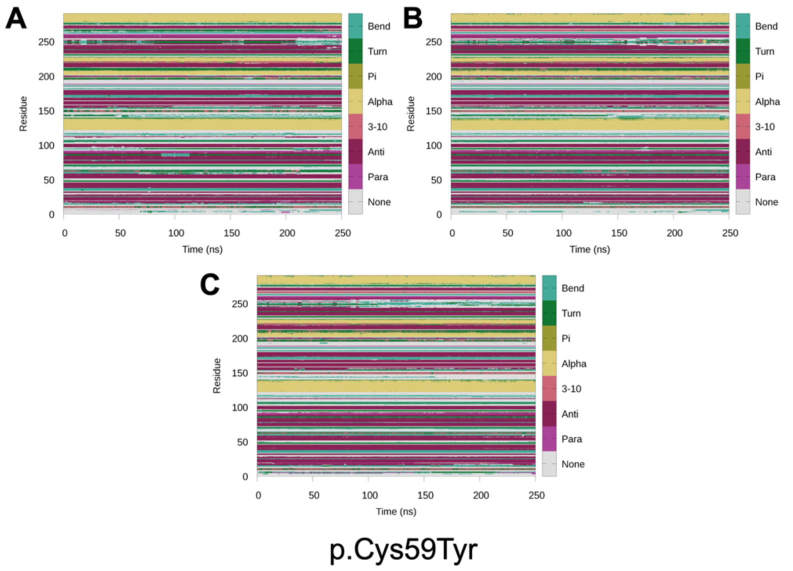

Supplementary Figure 16. C59Y secondary structure. Secondary structure analysis of each replicate of C59Y.

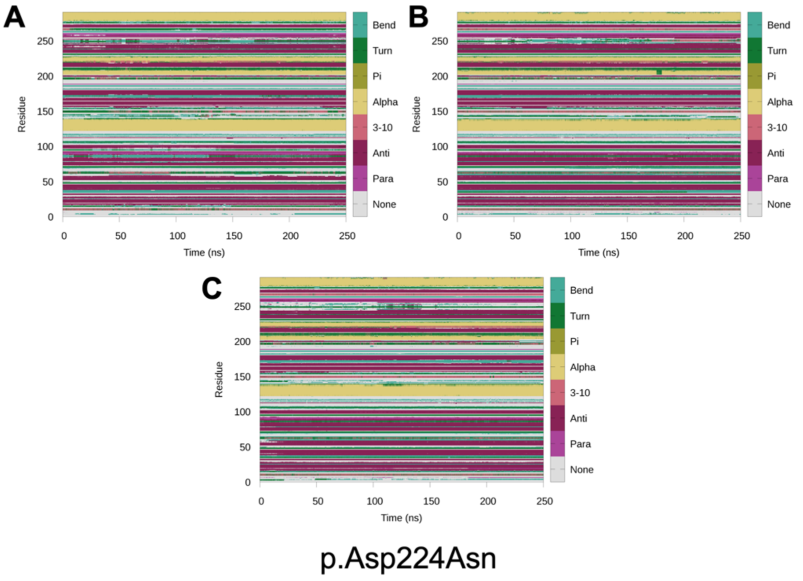

Supplementary Figure 17. D224N secondary structure. Secondary structure analysis of each replicate of D224N.

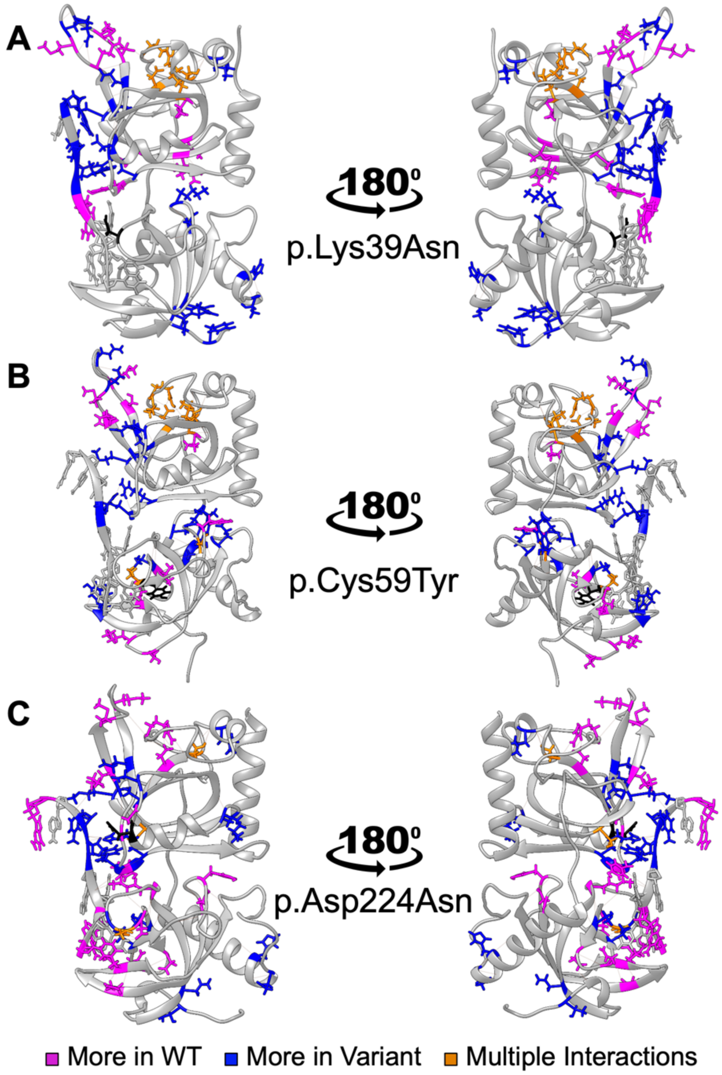

Supplementary Figure 18. Differences in hydrogen bond interactions. Differences in hydrogen bond interactions greater than 20% of the total simulation time for (A) WT and K39N, (B) WT and C59Y, and (C) WT and D224N. Interactions that are present for a larger percentage of simulation time in the WT are pink, and those more present in the variant structures are blue. Residues that had a combination of hydrogen bond interactions favored between both WT and the variant are shown in orange. The mutation location is shown in black, and all data are shown on the respective variant structure.

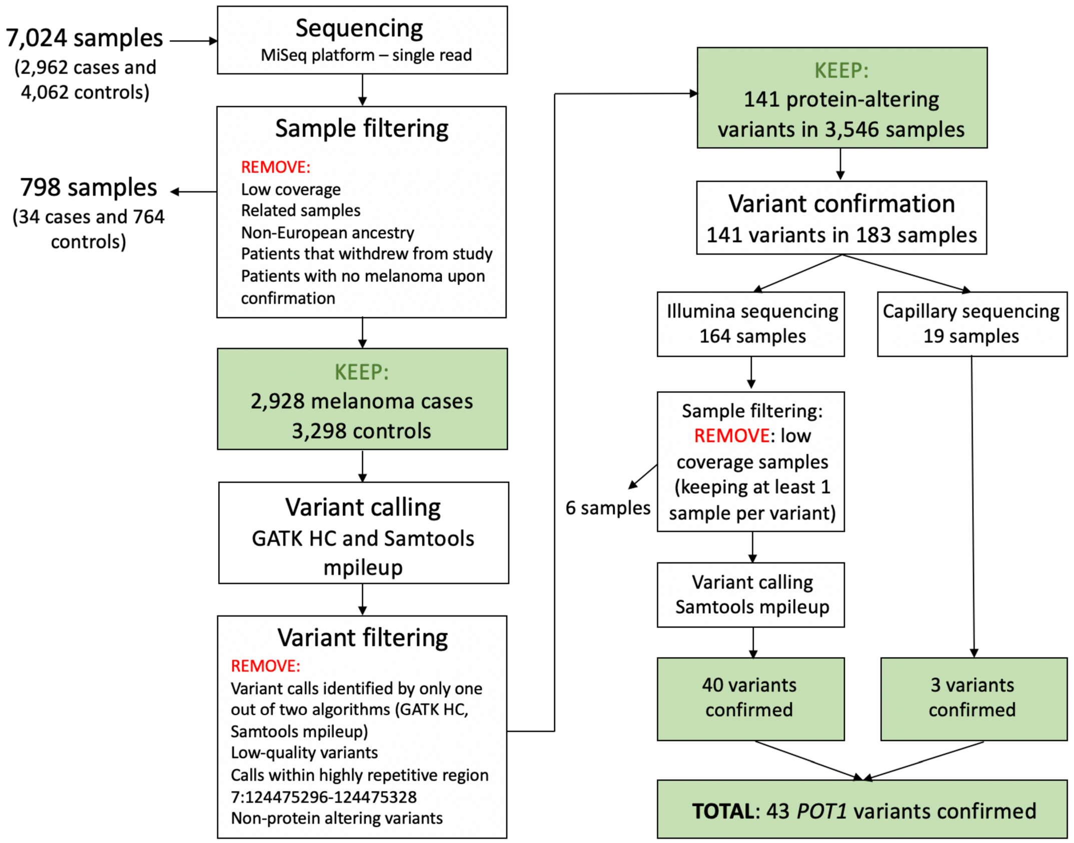

Supplementary Figure 19. Flowchart with the analysis steps followed in this study.

Supplementary Movie. First and second normal modes of the WT, K39N, C59Y, and D224N POT1-ssDNA variants from molecular dynamics simulations. The mutation position is colored pink.

Supplementary Tables and legends

Supplementary Table 1. All protein-altering variants in POT1 found in this study. Coordinates are in the GRCh37 reference genome. Consequences were predicted with VEP, Ensembl release 104, which also annotated co-existing variation, SIFT, PolyPhen and ClinVar pathogenicity predictions, and allele frequencies in the gnomAD database. All variants in this table were confirmed by resequencing of the original samples by a different method.

Supplementary Table 2. *POT1* variants classified as Group 1. Coordinates are from the GRCh37 reference genome. Consequences were predicted with VEP, Ensembl release 104, which also annotated co-existing variation, SIFT, PolyPhen and ClinVar pathogenicity predictions, and allele frequencies in the gnomAD database. All variants in this table were confirmed by resequencing of the original samples by a different method.

Supplementary Table 3. Age of onset statistics by pathogenicity group. Age of onset was not available for all participants.

Supplementary Table 4. Gender distribution by pathogenicity group in cases and controls. Please note there were two cases for which gender was unknown.

Supplementary Table 5. Family history of melanoma by pathogenicity group. *P*-value (Fisher's exact test): 0.88. Five cases were excluded from this comparison for lack of information.

Supplementary Table 6. Site of melanoma distribution by pathogenicity group.

Supplementary Table 7. Linear model used for adjusting telomere lengths by cohort.

Supplementary Table 8. Change in interactions with K39N. Residues that showed the greatest differences (K39N – WT) across systems in their total interaction energy with respect to the K39N residue. The average total interaction energy ± average standard deviation is provided. All values are in kcal mol^{-1}.

Supplementary Table 9. Change in interactions with D224N. Residues that showed the greatest differences (D224N – WT) across systems in their total interaction energy with respect to the D224N residue. The average total interaction energy ± average standard deviation is provided. All values are in kcal mol^{-1}.

Supplementary Table 10. Change in interactions with dG6 for K39N. Residues that showed the greatest differences (K39N – WT) across systems in their total interaction energy with respect to the dG6 residue. The average total interaction energy ± average standard deviation is provided. All values are in kcal mol^{-1}.

Supplementary Table 11. Change in interactions with dG6 for C59Y. Residues that showed the greatest differences (C59Y – WT) across systems in their total interaction energy with respect to the dG6 residue. The average total interaction energy ± average standard deviation is provided. All values are in kcal mol^{-1}.

Supplementary Table 12. Change in interactions with dG6 for D224N. Residues that showed the greatest differences (D224N – WT) across systems in their total interaction energy with respect to the dG6 residue. The average total interaction energy ± average standard deviation is provided. All values are in kcal mol^{-1}.

Supplementary Table 13. Samples included in this study. The list shows all 6,226 samples in this study, with their ID, proportion of high-quality bases sequenced in the 1st sequencing round, case/control status and cohort of origin.

Supplementary Table 14. All protein-altering variants in POT1 found in this study after the first round of sequencing. Coordinates are in the GRCh37 reference genome. Details of the number of samples that were re-sequenced for confirmation and other information are included. Variants confirmed are highlighted in green.

Supplementary Table 15. List of samples that were re-sequenced by Illumina for confirmation. The average coverage of coding *POT1* exons is included. In red, samples with a coverage lower than 10.

Supplementary Table 16. List of samples that were re-sequenced by capillary. Information about the variants re-sequenced and the result of the experiment is included. In green, samples and variants that were confirmed.

Supplementary Table 17. List of samples with variants in known melanoma predisposition genes. Genes *CDK4*, *CDKN2A* and *BAP1* were checked for variants. Only samples that were resequenced by Illumina (Supplementary Table 15) were assessed.

References

1 Newton-Bishop JA, Chang Y-M, Iles MM, Taylor JC, Bakker B, Chan M, Leake S, Karpavicius B, Haynes S, Fitzgibbon E, Elliott F, Kanetsky PA, Harland M, Barrett JH, Bishop DT. Melanocytic nevi, nevus genes, and melanoma risk in a large case-control study in the United Kingdom. *Cancer Epidemiol Biomarkers Prev* 2010;**19**:2043–54.

2 Pooley KA, Tyrer J, Shah M, Driver KE, Leyland J, Brown J, Audley T, McGuffog L, Ponder BAJ, Pharoah PDP, Easton DF, Dunning AM. No association between TERT-CLPTM1L single nucleotide polymorphism rs401681 and mean telomere length or cancer risk. *Cancer Epidemiol Biomarkers Prev* 2010;**19**:1862–5.

3 Wellcome Trust Case Control Consortium. Genome-wide association study of 14,000 cases of seven common diseases and 3,000 shared controls. *Nature* 2007;**447**:661–78.

4 Salomon-Ferrer R, Götz AW, Poole D, Le Grand S, Walker RC. Routine Microsecond Molecular Dynamics Simulations with AMBER on GPUs. 2. Explicit Solvent Particle Mesh Ewald. *Journal of Chemical Theory and Computation* 2013;**9**:3878–88.

5 Götz AW, Williamson MJ, Xu D, Poole D, Le Grand S, Walker RC. Routine Microsecond Molecular Dynamics Simulations with AMBER on GPUs. 1. Generalized Born. *Journal of Chemical Theory and Computation* 2012;**8**:1542–55.

6 Case DA, Ben-Shalom IY, Brozell SR, Cerutti DS, Cheatham III TE, Cruzeiro VWD, Darden TA, Duke RE, Ghoreishi D, Giambasu G, Giese T, Gilson MK, Gohlke H, Goetz AW, Greene D, Harris R, Homeyer N, Huang Y, Izadi S, Kovalenko A, Krasny R, Kurtzman T, Lee TS, LeGrand S, Li P, Lin C, Liu J, Luchko T, Luo R, Man V, Mermelstein DJ, Merz KM, Miao Y, Monard G, Nguyen C, Nguyen H, Onufriev A, Pan F, Qi R, Roe DR, Roitberg A, Sagui C, Schott-Verdugo S, Shen J, Simmerling CL, Smith J, Swails J, Walker RC, Wang J, Wei H, Wilson L, Wolf RM, Wu X, Xiao L, Xiong Y, York DM, Kollman PA. Amber19. 2019.

7 Tian C, Kasavajhala K, Belfon KAA, Raguette L, Huang H, Migues AN, Bickel J, Wang Y, Pincay J, Wu Q, Simmerling C. ff19SB: Amino-Acid-Specific Protein Backbone Parameters Trained against Quantum Mechanics Energy Surfaces in Solution. *Journal of Chemical Theory and Computation* 2020;**16**:528–52.

8 Lei M, Podell ER, Cech TR. Crystal structure of human POT1 bound to telomeric single-stranded DNA (TTAGGGTTAG). 2004. doi:10.2210/pdb1XJV/pdb

9 Lei M, Podell ER, Cech TR. Structure of human POT1 bound to telomeric single-stranded DNA provides a model for chromosome end-protection. *Nat Struct Mol Biol* 2004;**11**:1223–9.

10 Emsley P, Lohkamp B, Scott WG, Cowtan K. Features and development of Coot. *Acta Cryst D* 2010;**66**:486–501.

11 Chen VB, Arendall WB, Headd JJ, Keedy DA, Immormino RM, Kapral GJ, Murray LW, Richardson JS, Richardson DC. MolProbity: all-atom structure validation for macromolecular crystallography. *Acta Cryst D* 2010;**66**:12–21.

12 Sali A \u, Blundell TL. Comparative Protein Modelling by Satisfaction of Spatial Restraints. *Journal of Molecular Biology* 1993;**234**:779–815.

13 Fiser A, Do RKG, Šali A. Modeling of loops in protein structures. *Protein Science* 2000;**9**:1753–73.

14 Pettersen EF, Goddard TD, Huang CC, Couch GS, Greenblatt DM, Meng EC, Ferrin TE. UCSF Chimera—A visualization system for exploratory research and analysis. *Journal of Computational Chemistry* 2004;**25**:1605–12.
15 Dunbrack RL. Rotamer Libraries in the 21st Century. *Current Opinion in Structural Biology* 2002;**12**:431–40.
16 Jorgensen WL, Chandrasekhar J, Madura JD, Impey RW, Klein ML. Comparison of simple potential functions for simulating liquid water. *The Journal of Chemical Physics* 1983;**79**:926–35.
17 Essmann U, Perera L, Berkowitz ML, Darden T, Lee H, Pedersen LG. A smooth particle mesh Ewald method. *The Journal of Chemical Physics* 1995;**103**:8577–93.
18 Roe DR, Cheatham TE. PTRAJ and CPPTRAJ: Software for Processing and Analysis of Molecular Dynamics Trajectory Data. *Journal of Chemical Theory and Computation* 2013;**9**:3084–95.
19 Bakan A, Bahar I, Meireles LM. ProDy: Protein Dynamics Inferred from Theory and Experiments. *Bioinformatics* 2011;**27**:1575–7.
20 Humphrey W, Dalke A, Schulten K. VMD: Visual molecular dynamics. *Journal of Molecular Graphics* 1996;**14**:33–8.
21 Leddin E, Group CR, Cisneros GA. CisnerosResearch/AMBER-EDA: First Release. 2020. doi:10.5281/zenodo.4469902
22 R Core Team. *R: A Language and Environment for Statistical Computing*. Vienna, Austria: : R Foundation for Statistical Computing 2018. https://www.R-project.org/
23 Dowle M, Srinivasan A. *data.table: Extension of `data.frame`*. 2018. https://CRAN.R-project.org/package=data.table
24 Plate T, Heiberger R. *abind: Combine Multidimensional Arrays*. 2016. https://CRAN.R-project.org/package=abind
25 Wickham H. *tidyverse: Easily Install and Load the 'Tidyverse'*. 2017. https://CRAN.R-project.org/package=tidyverse
26 Hou T, Wang J, Li Y, Wang W. Assessing the Performance of the MM/PBSA and MM/GBSA Methods. 1. The Accuracy of Binding Free Energy Calculations Based on Molecular Dynamics Simulations. *J Chem Inf Model* 2011;**51**:69–82.
27 Miller BRI, McGee TDJr, Swails JM, Homeyer N, Gohlke H, Roitberg AE. MMPBSA.py: An Efficient Program for End-State Free Energy Calculations. *J Chem Theory Comput* 2012;**8**:3314–21.
28 Case DA, Aktulga HM, Belfon K, I.Y. Ben-Shalom, J.T. Berryman, S.R. Brozell, D.S. Cerutti, T.E., Cheatham, III, G.A. Cisneros, V.W.D. Cruzeiro, T.A. Darden, R.E. Duke, G. Giambasu, M.K. Gilson, H., Gohlke, A.W. Goetz, R. Harris, S. Izadi, S.A. Izmailov, K. Kasavajhala, M.C. Kaymak, E. King, A. Kovalenko, T. Kurtzman, T.S. Lee, S. LeGrand, P. Li, C. Lin, J. Liu, T. Luchko, R. Luo, M. Machado, V., Man, M. Manathunga, K.M. Merz, Y. Miao, O. Mikhailovskii, G. Monard, H. Nguyen, K.A. O'Hearn, A., Onufriev, F. Pan, S. Pantano, R. Qi, A. Rahnamoun, D.R. Roe, A. Roitberg, C. Sagui, S. Schott-Verdugo, A. Shajan, J. Shen, C.L. Simmerling, N.R. Skrynnikov, J. Smith, J. Swails, R.C. Walker, J. Wang, J. Wang,, H. Wei, R.M. Wolf, X. Wu, Y. Xiong, Y. Xue, D.M. York, S. Zhao, and P.A. Kollman. Amber 2022. 2022. University of California, San Francisco.

29 Williams T, Kelley C. Gnuplot 5.2: an interactive plotting program. 2019.http://gnuplot.sourceforge.net/

30 Hunter JD. Matplotlib: A 2D graphics environment. *Computing in Science & Engineering* 2007;**9**:90–5.

31 Wickham H. *ggplot2: Elegant Graphics for Data Analysis*. Springer-Verlag New York 2016. https://ggplot2.tidyverse.org
