# Supplementary material for: Population-based analysis of POT1 variants in a cutaneous melanoma case–control cohort
Source: J Med Genet. 2022 Dec 20;60(7):692–6. doi: 10.1136/jmg-2022-108776 (PMC10279804; doi:10.1136/jmg-2022-108776)
Supplement: Supplementary data [file jmg-2022-108776supp005.pdf]

Supplementary Table 7. Linear model used for adjusting telomere lengths by cohort.

Call:

lm(formula = tel ~ cohort, data = full_data, subset = case == control)

Residuals:

Min	1Q	Median	3Q	Max
-301.52	-107.17	-20.56	46.31	814.11

Coefficients

	Estimate Std.	Error	t value	Pr(>|t|)	
**(Intercept)**	563.49	24.37	23.12	< 2e-16	***
**Cohort: Cambridge**	-116.38	32.73	-3.556	0.000542	***
**Cohort: WTCCC**	-173.1	54.95	-3.15	0.002064	**

Signif. codes: 0 ‘***’ 0.001 ‘**’ 0.01 ‘*’ 0.05 ‘.’ 0.1 ‘ ’ 1

Residual standard error: 170.6 on 119 degrees of freedom

(37 observations deleted due to missingness)
Multiple R-squared:
0.1252, Adjusted R-squared: 0.1105

F-statistic: 8.512 on 2 and 119 DF, p-value: 0.0003506

Supplementary Table 8. Change in interactions with K39N. Residues that showed the greatest differences (K39N – WT) across systems in their total interaction energy with respect to the K39N residue. The average total interaction energy ± average standard deviation is provided. All values are in kcal mol^{-1}.

	K39N – WT
Ala6	-7.42 ± 0.01
Lys18	-9.83 ± 0.02
Lys30	-11.88 ± 0.02
Lys33	-25.84 ± 0.11
Pro34	1.05 ± 0.01
Leu37	2.27 ± 0.04
Asp42	25.20 ± 0.02
Asp51	9.96 ± 0.01
Lys56	-13.17 ± 0.01
Glu67	13.36 ± 0.09
Lys74	-10.33 ± 0.05
Asp77	11.35 ± 0.04
Arg80	-9.92 ± 0.01
Arg83	-10.83 ± 0.03
Lys85	-14.64 ± 0.04
Lys90	-20.02 ± 0.04
Lys91	-13.28 ± 0.08
Glu92	13.97 ± 0.08
Glu107	9.75 ± 0.04
Arg117	-10.99 ± 0.05
Lys121	-9.29 ± 0.04
Glu128	7.61 ± 0.01
Asp129	9.00 ± 0.01
Lys131	-7.24 ± 0.01

Glu134	7.64 ± 0.01
Arg137	-8.59 ± 0.03
Lys152	-8.87 ± 0.05
Asp155	8.37 ± 0.03
Asp163	12.76 ± 0.05
Lys171	-13.01 ± 0.17
Glu173	14.63 ± 0.24
Asp175	15.10 ± 0.25
Lys182	-9.87 ± 0.08
Asp185	9.23 ± 0.05
Arg188	-7.60 ± 0.04
Arg195	-9.10 ± 0.04
Asp200	8.91 ± 0.08
Glu204	10.90 ± 0.12
Asp206	8.41 ± 0.06
Arg212	-7.42 ± 0.05
Asp219	10.27 ± 0.08
Asp224	25.66 ± 0.78
Arg231	-17.86 ± 0.34
Lys234	-10.94 ± 0.12
Arg240	-11.45 ± 0.08
Lys247	-11.71 ± 0.06
Glu254	7.68 ± 0.09
Glu262	11.20 ± 0.07
Gly268	-1.31 ± 0.04
Arg273	-18.62 ± 0.17
Arg276	-14.51 ± 0.08
Glu280	9.27 ± 0.06
Asp284	9.78 ± 0.04
Asp286	8.08 ± 0.04

Lys289	-8.51 ± 0.05
Lys290	-7.54 ± 0.03
Asp291	8.09 ± 0.04
Glu293	7.17 ± 0.03
Ala299	6.71 ± 0.03
dT1	9.38 ± 0.14
dT2	32.79 ± 0.71
dA3	47.18 ± 1.32
dG4	27.20 ± 0.46
dG5	17.45 ± 0.28
dG6	18.03 ± 0.38
dT7	15.12 ± 0.29
dT8	17.25 ± 0.41
dA9	19.70 ± 0.82
dG10	12.51 ± 0.59

Supplementary Table 9. Change in interactions with D224N. Residues that showed the greatest differences (D224N – WT) across systems in their total interaction energy with respect to the D224N residue. The average total interaction energy ± average standard deviation is provided. All values are in kcal mol^{-1}.

	D224N – WT
Ala6	6.59 ± 0.02
Lys18	7.97 ± 0.05
Lys30	11.83 ± 0.03
Lys33	23.01 ± 0.11
Leu37	-1.06 ± 0.05
Lys39	26.00 ± 0.79
Asp42	-15.03 ± 0.19
Asp51	-9.06 ± 0.02
Lys56	12.19 ± 0.03
Glu67	-12.81 ± 0.11
Lys74	11.63 ± 0.02
Asp77	-11.84 ± 0.01
Arg80	8.79 ± 0.02
Arg83	8.53 ± 0.04
Lys85	9.92 ± 0.06
Lys90	14.16 ± 0.11
Lys91	10.66 ± 0.06
Glu92	-11.49 ± 0.04
Glu107	-10.35 ± 0.01
Arg117	11.35 ± 0.03
Lys121	8.55 ± 0.05
Glu128	-7.02 ± 0.01

Asp129	-8.20 ± 0.02
Lys131	6.78 ± 0.01
Glu134	-7.63 ± 0.01
Arg137	8.72 ± 0.01
Lys152	11.27 ± 0.02
Asp155	-10.60 ± 0.03
Asp163	-16.07 ± 0.04
Lys171	18.41 ± 0.02
Glu173	-24.24 ± 0.09
Val174	1.31 ± 0.01
Asp175	-32.49 ± 0.33
Ala177	-4.17 ± 0.21
Ser178	1.19 ± 0.05
Lys182	15.15 ± 0.06
Asp185	-12.22 ± 0.02
Arg188	10.21 ± 0.03
Arg195	13.68 ± 0.08
Asp200	-12.76 ± 0.06
Glu204	-14.84 ± 0.01
Asp206	-11.05 ± 0.01
Arg212	9.52 ± 0.02
Asp219	-16.37 ± 0.08
His226	3.76 ± 0.09
Val227	3.45 ± 0.07
His228	3.29 ± 0.14
Val229	1.34 ± 0.01
Ala230	1.42 ± 0.00

Arg231	29.38 ± 0.11
Lys234	12.94 ± 0.03
Arg240	13.76 ± 0.03
Lys247	19.67 ± 0.21
Glu254	-10.82 ± 0.09
Glu262	-19.03 ± 0.12
Leu265	-1.88 ± 0.03
His266	1.46 ± 0.21
Gly267	5.74 ± 0.12
Arg273	30.99 ± 0.18
Arg276	15.50 ± 0.07
Glu280	-10.55 ± 0.01
Asp284	-11.26 ± 0.01
Asp286	-9.21 ± 0.00
Lys289	9.88 ± 0.00
Lys290	8.73 ± 0.01
Asp291	-9.95 ± 0.02
Glu293	-8.66 ± 0.01
Ala299	-8.51 ± 0.02
dT1	-4.29 ± 0.11
dT2	-14.74 ± 0.37
dA3	-18.74 ± 0.52
dG4	-17.86 ± 0.36
dG5	-14.14 ± 0.21
dG6	-17.65 ± 0.27
dT7	-17.94 ± 0.21
dT8	-24.81 ± 0.39

dA9	-28.25 ± 0.53
dG10	-10.10 ± 1.25

Supplementary Table 10. Change in interactions with dG6 for K39N. Residues that showed the greatest differences (K39N – WT) across systems in their total interaction energy with respect to the dG6 residue. The average total interaction energy ± average standard deviation is provided. All values are in kcal mol^{-1}.

	K39N – WT
Lys30	2.41 ± 0.76
Lys33	11.95 ± 3.73
Lys39	18.03 ± 0.38
Lys56	4.10 ± 1.02
Lys74	1.11 ± 0.35
Asp77	-1.26 ± 0.37
Lys91	1.13 ± 0.69
Glu92	4.95 ± 0.75
Asp163	-2.28 ± 0.56
Arg240	2.04 ± 0.43
Thr269	-1.25 ± 0.23
Ser270	1.06 ± 0.75
Tyr271	1.59 ± 0.51
Gly272	1.14 ± 0.18
Arg273	2.47 ± 1.10
Asp284	-1.07 ± 0.25
dA9	-2.43 ± 0.39

Supplementary Table 11. Change in interactions with dG6 for C59Y. Residues that showed the greatest differences (C59Y – WT) across systems in their total interaction energy with respect to the dG6 residue. The average total interaction energy ± average standard deviation is provided. All values are in kcal mol^{-1}.

	C59Y – WT
Lys18	-1.01 ± 0.19
Lys33	8.00 ± 2.67
Asp51	1.28 ± 0.20
Lys56	-10.02 ± 0.80
Lys90	-2.62 ± 0.66
Glu92	5.88 ± 0.76
Asp163	1.31 ± 0.36
Asp224	1.13 ± 0.41
Lys247	-1.43 ± 0.23
Thr269	-1.09 ± 0.27
Ser270	-1.09 ± 0.50
Arg273	-1.88 ± 0.83
Arg276	-1.37 ± 0.25
dA3	-1.31 ± 0.29
dG4	-3.12 ± 0.70
dT8	1.02 ± 0.38
dA9	-1.94 ± 0.40

Supplementary Table 12. Change in interactions with dG6 for D224N. Residues that showed the greatest differences (D224N – WT) across systems in their total interaction energy with respect to the dG6 residue. The average total interaction energy ± average standard deviation is provided. All values are in kcal mol^{-1}.

	D224N – WT
Lys30	1.71 ± 0.94
Lys33	11.17 ± 3.92
Lys39	2.07 ± 3.92
Asp42	-1.02 ± 0.49
Lys56	3.74 ± 1.13
Glu67	-1.26 ± 0.33
Lys74	1.13 ± 0.41
Asp77	-1.21 ± 0.45
Lys90	1.76 ± 0.63
Lys91	2.06 ± 0.85
Glu92	4.17 ± 1.03
Asp163	-1.70 ± 0.77
Asp224	-17.65 ± 0.27
Arg240	1.72 ± 0.54
Tyr271	1.78 ± 0.49
Gly272	1.26 ± 0.18
Arg273	2.96 ± 1.27
dA3	-1.56 ± 0.43
dG4	-5.10 ± 0.99
dT8	1.11 ± 0.33

Supplementary Table 13. Samples included in this study. The list shows all 6,226 samples in this study, with their ID, proportion of high-quality bases sequenced in the 1st sequencing round, case/control status and cohort of origin.

Sample ID	Proportion of bases covered with high quality	Status	Cohort
Ctrl_1	1	Control	WTCCC
Ctrl_2	1	Control	WTCCC
Ctrl_3	1	Control	WTCCC
Ctrl_4	1	Control	WTCCC
Ctrl_5	1	Control	WTCCC
Ctrl_6	1	Control	WTCCC
Ctrl_7	1	Control	WTCCC
Ctrl_8	1	Control	WTCCC
Ctrl_9	1	Control	WTCCC
Ctrl_10	1	Control	WTCCC
Ctrl_11	1	Control	WTCCC
Ctrl_12	1	Control	WTCCC
Ctrl_13	1	Control	WTCCC
Ctrl_14	1	Control	WTCCC
Ctrl_15	1	Control	WTCCC
Ctrl_16	1	Control	WTCCC
Ctrl_17	1	Control	WTCCC
Ctrl_18	1	Control	WTCCC
Ctrl_19	1	Control	WTCCC
Ctrl_20	1	Control	WTCCC
Ctrl_21	1	Control	WTCCC
Ctrl_22	0.992650919	Control	WTCCC
Ctrl_23	1	Control	WTCCC
Ctrl_24	1	Control	WTCCC
Ctrl_25	1	Control	WTCCC
Ctrl_26	1	Control	WTCCC
Ctrl_27	1	Control	WTCCC
Ctrl_28	1	Control	WTCCC
Ctrl_29	1	Control	WTCCC
Ctrl_30	1	Control	WTCCC
Ctrl_31	1	Control	WTCCC
Ctrl_32	1	Control	WTCCC
Ctrl_33	1	Control	WTCCC

Ctrl_34	1	Control	WTCCC
Ctrl_35	1	Control	WTCCC
Ctrl_36	1	Control	WTCCC
Ctrl_37	1	Control	WTCCC
Ctrl_38	1	Control	WTCCC
Ctrl_39	1	Control	WTCCC
Ctrl_40	1	Control	WTCCC
Ctrl_41	1	Control	WTCCC
Ctrl_42	1	Control	WTCCC
Ctrl_43	1	Control	WTCCC
Ctrl_44	1	Control	WTCCC
Ctrl_45	1	Control	WTCCC
Ctrl_46	1	Control	WTCCC
Ctrl_47	1	Control	WTCCC
Ctrl_48	1	Control	WTCCC
Ctrl_49	1	Control	WTCCC
Ctrl_50	1	Control	WTCCC
Ctrl_51	1	Control	WTCCC
Ctrl_52	1	Control	WTCCC
Ctrl_53	1	Control	WTCCC
Ctrl_54	1	Control	WTCCC
Ctrl_55	1	Control	WTCCC
Ctrl_56	1	Control	WTCCC
Ctrl_57	1	Control	WTCCC
Ctrl_58	1	Control	WTCCC
Ctrl_59	1	Control	WTCCC
Ctrl_60	1	Control	WTCCC
Ctrl_61	1	Control	WTCCC
Ctrl_62	1	Control	WTCCC
Ctrl_63	1	Control	WTCCC
Ctrl_64	1	Control	WTCCC
Ctrl_65	1	Control	WTCCC
PD30749a	1	Control	WTCCC
Ctrl_66	1	Control	WTCCC
Ctrl_67	1	Control	WTCCC
Ctrl_68	1	Control	WTCCC
Ctrl_69	1	Control	WTCCC
Ctrl_70	1	Control	WTCCC
Ctrl_71	1	Control	WTCCC
Ctrl_72	1	Control	WTCCC

Ctrl_73	1	Control	WTCCC
Ctrl_74	1	Control	WTCCC
Ctrl_75	1	Control	WTCCC
PD30746a	1	Control	WTCCC
Ctrl_76	1	Control	WTCCC
Ctrl_77	1	Control	WTCCC
Ctrl_78	1	Control	WTCCC
Ctrl_79	1	Control	WTCCC
Ctrl_80	0.940682415	Control	WTCCC
Ctrl_81	1	Control	WTCCC
Ctrl_82	1	Control	WTCCC
Ctrl_83	1	Control	WTCCC
Ctrl_84	1	Control	WTCCC
Ctrl_85	1	Control	WTCCC
Ctrl_86	1	Control	WTCCC
Ctrl_87	1	Control	WTCCC
Ctrl_88	1	Control	WTCCC
Ctrl_89	1	Control	WTCCC
Ctrl_90	1	Control	WTCCC
Ctrl_91	1	Control	WTCCC
Ctrl_92	1	Control	WTCCC
Ctrl_93	1	Control	WTCCC
Ctrl_94	1	Control	WTCCC
Ctrl_95	1	Control	WTCCC
Ctrl_96	1	Control	WTCCC
Ctrl_97	1	Control	WTCCC
Ctrl_98	1	Control	WTCCC
Ctrl_99	1	Control	WTCCC
Ctrl_100	1	Control	WTCCC
Ctrl_101	1	Control	WTCCC
Ctrl_102	1	Control	WTCCC
Ctrl_103	1	Control	WTCCC
Ctrl_104	1	Control	WTCCC
Ctrl_105	1	Control	WTCCC
Ctrl_106	1	Control	WTCCC
Ctrl_107	1	Control	WTCCC
Ctrl_108	1	Control	WTCCC
Ctrl_109	1	Control	WTCCC
Ctrl_110	1	Control	WTCCC
Ctrl_111	1	Control	WTCCC

Ctrl_112	1	Control	WTCCC
Ctrl_113	1	Control	WTCCC
Ctrl_114	1	Control	WTCCC
Ctrl_115	1	Control	WTCCC
Ctrl_116	1	Control	WTCCC
Ctrl_117	1	Control	WTCCC
Ctrl_118	1	Control	WTCCC
Ctrl_119	1	Control	WTCCC
Ctrl_120	1	Control	WTCCC
Ctrl_121	1	Control	WTCCC
Ctrl_122	1	Control	WTCCC
Ctrl_123	1	Control	WTCCC
Ctrl_124	1	Control	WTCCC
Ctrl_125	1	Control	WTCCC
Ctrl_126	1	Control	WTCCC
Ctrl_127	0.998950131	Control	WTCCC
Ctrl_128	1	Control	WTCCC
Ctrl_129	1	Control	WTCCC
Ctrl_130	1	Control	WTCCC
Ctrl_131	1	Control	WTCCC
Ctrl_132	1	Control	WTCCC
Ctrl_133	1	Control	WTCCC
Ctrl_134	1	Control	WTCCC
Ctrl_135	1	Control	WTCCC
Ctrl_136	1	Control	WTCCC
Ctrl_137	1	Control	WTCCC
Ctrl_138	1	Control	WTCCC
Ctrl_139	1	Control	WTCCC
Ctrl_140	1	Control	WTCCC
Ctrl_141	1	Control	WTCCC
Ctrl_142	1	Control	WTCCC
Ctrl_143	1	Control	WTCCC
Ctrl_144	1	Control	WTCCC
Ctrl_145	1	Control	WTCCC
Ctrl_146	1	Control	WTCCC
Ctrl_147	1	Control	WTCCC
Ctrl_148	1	Control	WTCCC
Ctrl_149	1	Control	WTCCC
Ctrl_150	1	Control	WTCCC
Ctrl_151	1	Control	WTCCC

Ctrl_152	1	Control	WTCCC
Ctrl_153	1	Control	WTCCC
Ctrl_154	1	Control	WTCCC
Ctrl_155	1	Control	WTCCC
Ctrl_156	1	Control	WTCCC
Ctrl_157	1	Control	WTCCC
Ctrl_158	1	Control	WTCCC
Ctrl_159	1	Control	WTCCC
Ctrl_160	1	Control	WTCCC
Ctrl_161	1	Control	WTCCC
Ctrl_162	0.951181102	Control	WTCCC
Ctrl_163	1	Control	WTCCC
Ctrl_164	1	Control	WTCCC
Ctrl_165	1	Control	WTCCC
Ctrl_166	0.995275591	Control	WTCCC
Ctrl_167	1	Control	WTCCC
Ctrl_168	1	Control	WTCCC
Ctrl_169	1	Control	WTCCC
Ctrl_170	1	Control	WTCCC
Ctrl_171	1	Control	WTCCC
Ctrl_172	1	Control	WTCCC
Ctrl_173	1	Control	WTCCC
Ctrl_174	1	Control	WTCCC
Ctrl_175	1	Control	WTCCC
Ctrl_176	1	Control	WTCCC
Ctrl_177	1	Control	WTCCC
Ctrl_178	1	Control	WTCCC
Ctrl_179	1	Control	WTCCC
Ctrl_180	1	Control	WTCCC
Ctrl_181	1	Control	WTCCC
Ctrl_182	1	Control	WTCCC
Ctrl_183	1	Control	WTCCC
Ctrl_184	1	Control	WTCCC
Ctrl_185	1	Control	WTCCC
Ctrl_186	1	Control	WTCCC
Ctrl_187	1	Control	WTCCC
Ctrl_188	0.992650919	Control	WTCCC
Ctrl_189	1	Control	WTCCC
Ctrl_190	1	Control	WTCCC
Ctrl_191	1	Control	WTCCC

Ctrl_192	1	Control	WTCCC
Ctrl_193	1	Control	WTCCC
Ctrl_194	1	Control	WTCCC
Ctrl_195	1	Control	WTCCC
Ctrl_196	1	Control	WTCCC
Ctrl_197	1	Control	WTCCC
Ctrl_198	1	Control	WTCCC
Ctrl_199	1	Control	WTCCC
Ctrl_200	1	Control	WTCCC
Ctrl_201	1	Control	WTCCC
Ctrl_202	1	Control	WTCCC
Ctrl_203	1	Control	WTCCC
Ctrl_204	1	Control	WTCCC
Ctrl_205	1	Control	WTCCC
Ctrl_206	1	Control	WTCCC
Ctrl_207	1	Control	WTCCC
Ctrl_208	1	Control	WTCCC
Ctrl_209	1	Control	WTCCC
Ctrl_210	1	Control	WTCCC
Ctrl_211	1	Control	WTCCC
Ctrl_212	1	Control	WTCCC
Ctrl_213	1	Control	WTCCC
Ctrl_214	1	Control	WTCCC
Ctrl_215	1	Control	WTCCC
Ctrl_216	1	Control	WTCCC
Ctrl_217	1	Control	WTCCC
Ctrl_218	1	Control	WTCCC
Ctrl_219	1	Control	WTCCC
Ctrl_220	0.951706037	Control	WTCCC
Ctrl_221	1	Control	WTCCC
Ctrl_222	1	Control	WTCCC
Ctrl_223	1	Control	WTCCC
PD30751a	1	Control	WTCCC
Ctrl_224	1	Control	WTCCC
Ctrl_225	1	Control	WTCCC
Ctrl_226	1	Control	WTCCC
PD30759a	1	Control	WTCCC
Ctrl_227	1	Control	WTCCC
Ctrl_228	1	Control	WTCCC
Ctrl_229	1	Control	WTCCC

Ctrl_230	1	Control	WTCCC
Ctrl_231	1	Control	WTCCC
Ctrl_232	1	Control	WTCCC
Ctrl_233	1	Control	WTCCC
Ctrl_234	1	Control	WTCCC
PD30752a	1	Control	WTCCC
Ctrl_235	1	Control	WTCCC
Ctrl_236	1	Control	WTCCC
Ctrl_237	1	Control	WTCCC
Ctrl_238	1	Control	WTCCC
Ctrl_239	1	Control	WTCCC
Ctrl_240	1	Control	WTCCC
Ctrl_241	1	Control	WTCCC
Ctrl_242	1	Control	WTCCC
Ctrl_243	1	Control	WTCCC
Ctrl_244	1	Control	WTCCC
Ctrl_245	1	Control	WTCCC
Ctrl_246	1	Control	WTCCC
Ctrl_247	1	Control	WTCCC
Ctrl_248	1	Control	WTCCC
Ctrl_249	1	Control	WTCCC
Ctrl_250	1	Control	WTCCC
Ctrl_251	1	Control	WTCCC
Ctrl_252	1	Control	WTCCC
Ctrl_253	1	Control	WTCCC
Ctrl_254	1	Control	WTCCC
Ctrl_255	1	Control	WTCCC
Ctrl_256	1	Control	WTCCC
Ctrl_257	1	Control	WTCCC
Ctrl_258	1	Control	WTCCC
Ctrl_259	1	Control	WTCCC
Ctrl_260	1	Control	WTCCC
Ctrl_261	1	Control	WTCCC
Ctrl_262	1	Control	WTCCC
Ctrl_263	1	Control	WTCCC
Ctrl_264	1	Control	WTCCC
Ctrl_265	1	Control	WTCCC
Ctrl_266	1	Control	WTCCC
Ctrl_267	1	Control	WTCCC
Ctrl_268	1	Control	WTCCC

Ctrl_269	1	Control	WTCCC
PD30750a	1	Control	WTCCC
Ctrl_270	1	Control	WTCCC
Ctrl_271	1	Control	WTCCC
Ctrl_272	0.94855643	Control	WTCCC
Ctrl_273	1	Control	WTCCC
Ctrl_274	1	Control	WTCCC
Ctrl_275	1	Control	WTCCC
Ctrl_276	1	Control	WTCCC
Ctrl_277	1	Control	WTCCC
Ctrl_278	1	Control	WTCCC
Ctrl_279	1	Control	WTCCC
Ctrl_280	1	Control	WTCCC
Ctrl_281	1	Control	WTCCC
Ctrl_282	1	Control	WTCCC
Ctrl_283	1	Control	WTCCC
Ctrl_284	1	Control	WTCCC
Ctrl_285	1	Control	WTCCC
Ctrl_286	1	Control	WTCCC
Ctrl_287	1	Control	WTCCC
Ctrl_288	1	Control	WTCCC
Ctrl_289	1	Control	WTCCC
Ctrl_290	1	Control	WTCCC
Ctrl_291	1	Control	WTCCC
Ctrl_292	1	Control	WTCCC
Ctrl_293	1	Control	WTCCC
Ctrl_294	1	Control	WTCCC
Ctrl_295	1	Control	WTCCC
Ctrl_296	1	Control	WTCCC
Ctrl_297	1	Control	WTCCC
Ctrl_298	1	Control	WTCCC
Ctrl_299	1	Control	WTCCC
Ctrl_300	1	Control	WTCCC
Ctrl_301	1	Control	WTCCC
Ctrl_302	1	Control	WTCCC
Ctrl_303	1	Control	WTCCC
Ctrl_304	1	Control	WTCCC
Ctrl_305	1	Control	WTCCC
Ctrl_306	1	Control	WTCCC
Ctrl_307	1	Control	WTCCC

Ctrl_308	1	Control	WTCCC
Ctrl_309	1	Control	WTCCC
PD30760a	0.95328084	Control	WTCCC
Ctrl_310	1	Control	WTCCC
Ctrl_311	1	Control	WTCCC
PD30748a	1	Control	WTCCC
Ctrl_312	1	Control	WTCCC
Ctrl_313	1	Control	WTCCC
Ctrl_314	1	Control	WTCCC
Ctrl_315	1	Control	WTCCC
Ctrl_316	1	Control	WTCCC
Ctrl_317	1	Control	WTCCC
Ctrl_318	1	Control	WTCCC
Ctrl_319	1	Control	WTCCC
Ctrl_320	1	Control	WTCCC
Ctrl_321	1	Control	WTCCC
Ctrl_322	1	Control	WTCCC
Ctrl_323	1	Control	WTCCC
Ctrl_324	1	Control	WTCCC
Ctrl_325	1	Control	WTCCC
Ctrl_326	1	Control	WTCCC
Ctrl_327	1	Control	WTCCC
Ctrl_328	1	Control	WTCCC
Ctrl_329	1	Control	WTCCC
Ctrl_330	1	Control	WTCCC
Ctrl_331	1	Control	WTCCC
Ctrl_332	1	Control	WTCCC
Ctrl_333	1	Control	WTCCC
Ctrl_334	1	Control	WTCCC
Ctrl_335	1	Control	WTCCC
Ctrl_336	1	Control	WTCCC
Ctrl_337	1	Control	WTCCC
Ctrl_338	1	Control	WTCCC
Ctrl_339	1	Control	WTCCC
Ctrl_340	1	Control	WTCCC
Ctrl_341	1	Control	WTCCC
Ctrl_342	1	Control	WTCCC
Ctrl_343	0.951706037	Control	WTCCC
Ctrl_344	1	Control	WTCCC
Ctrl_345	1	Control	WTCCC

Ctrl_346	1	Control	WTCCC
Ctrl_347	1	Control	WTCCC
Ctrl_348	1	Control	WTCCC
Ctrl_349	1	Control	WTCCC
Ctrl_350	1	Control	WTCCC
Ctrl_351	1	Control	WTCCC
Ctrl_352	1	Control	WTCCC
PD30761a	1	Control	WTCCC
Ctrl_353	1	Control	WTCCC
Ctrl_354	1	Control	WTCCC
Ctrl_355	1	Control	WTCCC
Ctrl_356	1	Control	WTCCC
Ctrl_357	1	Control	WTCCC
Ctrl_358	1	Control	WTCCC
Ctrl_359	1	Control	WTCCC
Ctrl_360	1	Control	WTCCC
Ctrl_361	1	Control	WTCCC
Ctrl_362	1	Control	WTCCC
Ctrl_363	1	Control	WTCCC
Ctrl_364	1	Control	WTCCC
Ctrl_365	1	Control	WTCCC
Ctrl_366	1	Control	WTCCC
Ctrl_367	1	Control	WTCCC
Ctrl_368	1	Control	WTCCC
Ctrl_369	1	Control	WTCCC
Ctrl_370	1	Control	WTCCC
Ctrl_371	1	Control	WTCCC
Ctrl_372	1	Control	WTCCC
Ctrl_373	1	Control	WTCCC
Ctrl_374	1	Control	WTCCC
Ctrl_375	1	Control	WTCCC
Ctrl_376	1	Control	WTCCC
Ctrl_377	1	Control	WTCCC
Ctrl_378	1	Control	WTCCC
Ctrl_379	1	Control	WTCCC
Ctrl_380	1	Control	WTCCC
Ctrl_381	1	Control	WTCCC
Ctrl_382	1	Control	WTCCC
Ctrl_383	1	Control	WTCCC
Ctrl_384	1	Control	WTCCC

PD30743a	1	Control	WTCCC
Ctrl_385	1	Control	WTCCC
Ctrl_386	1	Control	WTCCC
Ctrl_387	1	Control	WTCCC
Ctrl_388	1	Control	WTCCC
Ctrl_389	1	Control	WTCCC
Ctrl_390	1	Control	WTCCC
Ctrl_391	0.999475066	Control	WTCCC
Ctrl_392	1	Control	WTCCC
Ctrl_393	1	Control	WTCCC
Ctrl_394	1	Control	WTCCC
Ctrl_395	1	Control	WTCCC
Ctrl_396	1	Control	WTCCC
Ctrl_397	1	Control	WTCCC
Ctrl_398	1	Control	WTCCC
Ctrl_399	1	Control	WTCCC
Ctrl_400	1	Control	WTCCC
Ctrl_401	1	Control	WTCCC
Ctrl_402	1	Control	WTCCC
Ctrl_403	1	Control	WTCCC
Ctrl_404	1	Control	WTCCC
Ctrl_405	1	Control	WTCCC
Ctrl_406	1	Control	WTCCC
Ctrl_407	1	Control	WTCCC
Ctrl_408	1	Control	WTCCC
Ctrl_409	1	Control	WTCCC
Ctrl_410	1	Control	WTCCC
Ctrl_411	1	Control	WTCCC
Ctrl_412	1	Control	WTCCC
Ctrl_413	1	Control	WTCCC
Ctrl_414	1	Control	WTCCC
Ctrl_415	1	Control	WTCCC
Ctrl_416	1	Control	WTCCC
Ctrl_417	1	Control	WTCCC
Ctrl_418	1	Control	WTCCC
PD30753a	1	Control	WTCCC
Ctrl_419	1	Control	WTCCC
Ctrl_420	1	Control	WTCCC
Ctrl_421	1	Control	WTCCC
Ctrl_422	1	Control	WTCCC

Ctrl_423	1	Control	WTCCC
Ctrl_424	1	Control	WTCCC
Ctrl_425	1	Control	WTCCC
Ctrl_426	1	Control	WTCCC
Ctrl_427	1	Control	WTCCC
Ctrl_428	1	Control	WTCCC
Ctrl_429	1	Control	WTCCC
Ctrl_430	1	Control	WTCCC
Ctrl_431	1	Control	WTCCC
Ctrl_432	1	Control	WTCCC
Ctrl_433	1	Control	WTCCC
Ctrl_434	1	Control	WTCCC
Ctrl_435	1	Control	WTCCC
Ctrl_436	1	Control	WTCCC
Ctrl_437	1	Control	WTCCC
Ctrl_438	1	Control	WTCCC
Ctrl_439	1	Control	WTCCC
Ctrl_440	1	Control	WTCCC
Ctrl_441	1	Control	WTCCC
Ctrl_442	1	Control	WTCCC
Ctrl_443	1	Control	WTCCC
Ctrl_444	1	Control	WTCCC
Ctrl_445	1	Control	WTCCC
Ctrl_446	1	Control	WTCCC
Ctrl_447	1	Control	WTCCC
Ctrl_448	1	Control	WTCCC
Ctrl_449	1	Control	WTCCC
Ctrl_450	1	Control	WTCCC
Ctrl_451	1	Control	WTCCC
Ctrl_452	1	Control	WTCCC
Ctrl_453	1	Control	WTCCC
Ctrl_454	1	Control	WTCCC
Ctrl_455	1	Control	WTCCC
Ctrl_456	1	Control	WTCCC
Ctrl_457	1	Control	WTCCC
Ctrl_458	1	Control	WTCCC
Ctrl_459	1	Control	WTCCC
Ctrl_460	1	Control	WTCCC
Ctrl_461	1	Control	WTCCC
Ctrl_462	1	Control	WTCCC

Ctrl_463	1	Control	WTCCC
Ctrl_464	1	Control	WTCCC
Ctrl_465	1	Control	WTCCC
Ctrl_466	1	Control	WTCCC
Ctrl_467	1	Control	WTCCC
Ctrl_468	1	Control	WTCCC
Ctrl_469	1	Control	WTCCC
Ctrl_470	1	Control	WTCCC
Ctrl_471	1	Control	WTCCC
Ctrl_472	1	Control	WTCCC
Ctrl_473	1	Control	WTCCC
Ctrl_474	1	Control	WTCCC
Ctrl_475	1	Control	WTCCC
Ctrl_476	1	Control	WTCCC
Ctrl_477	1	Control	WTCCC
Ctrl_478	0.997900262	Control	WTCCC
Ctrl_479	1	Control	WTCCC
Ctrl_480	1	Control	WTCCC
Ctrl_481	1	Control	WTCCC
Ctrl_482	1	Control	WTCCC
Ctrl_483	1	Control	WTCCC
Ctrl_484	1	Control	WTCCC
Ctrl_485	1	Control	WTCCC
Ctrl_486	1	Control	WTCCC
Ctrl_487	1	Control	WTCCC
Ctrl_488	1	Control	WTCCC
Ctrl_489	1	Control	WTCCC
Ctrl_490	1	Control	WTCCC
Ctrl_491	1	Control	WTCCC
Ctrl_492	1	Control	WTCCC
Ctrl_493	1	Control	WTCCC
Ctrl_494	1	Control	WTCCC
Ctrl_495	1	Control	WTCCC
Ctrl_496	1	Control	WTCCC
Ctrl_497	1	Control	WTCCC
Ctrl_498	1	Control	WTCCC
Ctrl_499	1	Control	WTCCC
Ctrl_500	1	Control	WTCCC
Ctrl_501	1	Control	WTCCC
Ctrl_502	1	Control	WTCCC

Ctrl_503	1	Control	WTCCC
Ctrl_504	1	Control	WTCCC
Ctrl_505	1	Control	WTCCC
Ctrl_506	1	Control	WTCCC
Ctrl_507	1	Control	WTCCC
Ctrl_508	1	Control	WTCCC
Ctrl_509	1	Control	WTCCC
Ctrl_510	1	Control	WTCCC
Ctrl_511	1	Control	WTCCC
Ctrl_512	1	Control	WTCCC
Ctrl_513	1	Control	WTCCC
Ctrl_514	1	Control	WTCCC
Ctrl_515	1	Control	WTCCC
Ctrl_516	1	Control	WTCCC
Ctrl_517	1	Control	WTCCC
Ctrl_518	1	Control	WTCCC
Ctrl_519	1	Control	WTCCC
Ctrl_520	1	Control	WTCCC
Ctrl_521	1	Control	WTCCC
Ctrl_522	1	Control	WTCCC
Ctrl_523	1	Control	WTCCC
Ctrl_524	1	Control	WTCCC
Ctrl_525	1	Control	WTCCC
Ctrl_526	1	Control	WTCCC
Ctrl_527	1	Control	WTCCC
Ctrl_528	1	Control	WTCCC
Ctrl_529	1	Control	WTCCC
Ctrl_530	1	Control	WTCCC
Ctrl_531	1	Control	WTCCC
Ctrl_532	1	Control	WTCCC
Ctrl_533	1	Control	WTCCC
Ctrl_534	1	Control	WTCCC
Ctrl_535	1	Control	WTCCC
Ctrl_536	1	Control	WTCCC
Ctrl_537	1	Control	WTCCC
Ctrl_538	1	Control	WTCCC
Ctrl_539	1	Control	WTCCC
Ctrl_540	1	Control	WTCCC
Ctrl_541	1	Control	WTCCC
Ctrl_542	1	Control	WTCCC

Ctrl_543	1	Control	WTCCC
Ctrl_544	1	Control	WTCCC
Ctrl_545	1	Control	WTCCC
Ctrl_546	1	Control	WTCCC
Ctrl_547	1	Control	WTCCC
Ctrl_548	1	Control	WTCCC
Ctrl_549	1	Control	WTCCC
Ctrl_550	1	Control	WTCCC
Ctrl_551	1	Control	WTCCC
Ctrl_552	1	Control	WTCCC
Ctrl_553	1	Control	WTCCC
Ctrl_554	1	Control	WTCCC
Ctrl_555	1	Control	WTCCC
Ctrl_556	1	Control	WTCCC
Ctrl_557	1	Control	WTCCC
Ctrl_558	1	Control	WTCCC
Ctrl_559	1	Control	WTCCC
Ctrl_560	1	Control	WTCCC
Ctrl_561	1	Control	WTCCC
Ctrl_562	0.94855643	Control	WTCCC
Ctrl_563	1	Control	WTCCC
Ctrl_564	1	Control	WTCCC
Ctrl_565	1	Control	WTCCC
Ctrl_566	1	Control	WTCCC
Ctrl_567	1	Control	WTCCC
Ctrl_568	0.94855643	Control	WTCCC
Ctrl_569	1	Control	WTCCC
Ctrl_570	0.94855643	Control	WTCCC
Ctrl_571	1	Control	WTCCC
Ctrl_572	1	Control	WTCCC
Ctrl_573	1	Control	WTCCC
Ctrl_574	1	Control	WTCCC
Ctrl_575	1	Control	WTCCC
Ctrl_576	1	Control	WTCCC
Ctrl_577	1	Control	WTCCC
Ctrl_578	1	Control	WTCCC
Ctrl_579	1	Control	WTCCC
Ctrl_580	1	Control	WTCCC
Ctrl_581	1	Control	WTCCC
Ctrl_582	1	Control	WTCCC

Ctrl_583	1	Control	WTCCC
Ctrl_584	1	Control	WTCCC
Ctrl_585	1	Control	WTCCC
Ctrl_586	1	Control	WTCCC
Ctrl_587	1	Control	WTCCC
Ctrl_588	1	Control	WTCCC
Ctrl_589	1	Control	WTCCC
Ctrl_590	1	Control	WTCCC
Ctrl_591	1	Control	WTCCC
Ctrl_592	1	Control	WTCCC
Ctrl_593	1	Control	WTCCC
Ctrl_594	1	Control	WTCCC
Ctrl_595	1	Control	WTCCC
Ctrl_596	1	Control	WTCCC
Ctrl_597	1	Control	WTCCC
Ctrl_598	1	Control	WTCCC
Ctrl_599	1	Control	WTCCC
Ctrl_600	1	Control	WTCCC
Ctrl_601	1	Control	WTCCC
Ctrl_602	1	Control	WTCCC
Ctrl_603	1	Control	WTCCC
Ctrl_604	1	Control	WTCCC
Ctrl_605	1	Control	WTCCC
Ctrl_606	1	Control	WTCCC
Ctrl_607	1	Control	WTCCC
Ctrl_608	1	Control	WTCCC
Ctrl_609	1	Control	WTCCC
Ctrl_610	1	Control	WTCCC
Ctrl_611	1	Control	WTCCC
Ctrl_612	1	Control	WTCCC
Ctrl_613	1	Control	WTCCC
Ctrl_614	1	Control	WTCCC
Ctrl_615	1	Control	WTCCC
Ctrl_616	1	Control	WTCCC
Ctrl_617	1	Control	WTCCC
Ctrl_618	1	Control	WTCCC
Ctrl_619	1	Control	WTCCC
Ctrl_620	1	Control	WTCCC
Ctrl_621	1	Control	WTCCC
Ctrl_622	1	Control	WTCCC

Ctrl_623	1	Control	WTCCC
Ctrl_624	1	Control	WTCCC
Ctrl_625	1	Control	WTCCC
Ctrl_626	1	Control	WTCCC
Ctrl_627	1	Control	WTCCC
Ctrl_628	1	Control	WTCCC
Ctrl_629	1	Control	WTCCC
Ctrl_630	1	Control	WTCCC
Ctrl_631	1	Control	WTCCC
Ctrl_632	1	Control	WTCCC
Ctrl_633	1	Control	WTCCC
Ctrl_634	1	Control	WTCCC
Ctrl_635	1	Control	WTCCC
Ctrl_636	1	Control	WTCCC
Ctrl_637	1	Control	WTCCC
Ctrl_638	1	Control	WTCCC
Ctrl_639	1	Control	WTCCC
Ctrl_640	1	Control	WTCCC
PD30740a	1	Control	WTCCC
Ctrl_641	1	Control	WTCCC
Ctrl_642	1	Control	WTCCC
Ctrl_643	1	Control	WTCCC
Ctrl_644	1	Control	WTCCC
Ctrl_645	1	Control	WTCCC
Ctrl_646	1	Control	WTCCC
Ctrl_647	1	Control	WTCCC
Ctrl_648	1	Control	WTCCC
Ctrl_649	1	Control	WTCCC
Ctrl_650	1	Control	WTCCC
Ctrl_651	1	Control	WTCCC
Ctrl_652	1	Control	WTCCC
Ctrl_653	1	Control	WTCCC
Ctrl_654	1	Control	WTCCC
Ctrl_655	1	Control	WTCCC
Ctrl_656	1	Control	WTCCC
Ctrl_657	1	Control	WTCCC
Ctrl_658	1	Control	WTCCC
Ctrl_659	1	Control	WTCCC
Ctrl_660	1	Control	WTCCC
Ctrl_661	1	Control	WTCCC

Ctrl_662	1	Control	WTCCC
Ctrl_663	1	Control	WTCCC
Ctrl_664	0.94855643	Control	WTCCC
Ctrl_665	1	Control	WTCCC
Ctrl_666	1	Control	WTCCC
Ctrl_667	1	Control	WTCCC
Ctrl_668	1	Control	WTCCC
Ctrl_669	1	Control	WTCCC
Ctrl_670	1	Control	WTCCC
Ctrl_671	1	Control	WTCCC
Ctrl_672	1	Control	WTCCC
Ctrl_673	1	Control	WTCCC
Ctrl_674	1	Control	WTCCC
Ctrl_675	1	Control	WTCCC
Ctrl_676	1	Control	WTCCC
Ctrl_677	1	Control	WTCCC
Ctrl_678	1	Control	WTCCC
PD30757a	1	Control	WTCCC
Ctrl_679	1	Control	WTCCC
Ctrl_680	1	Control	WTCCC
Ctrl_681	1	Control	WTCCC
Ctrl_682	1	Control	WTCCC
Ctrl_683	1	Control	WTCCC
Ctrl_684	1	Control	WTCCC
Ctrl_685	1	Control	WTCCC
Ctrl_686	1	Control	WTCCC
Ctrl_687	1	Control	WTCCC
Ctrl_688	1	Control	WTCCC
Ctrl_689	1	Control	WTCCC
PD30762a	1	Control	WTCCC
Ctrl_690	1	Control	WTCCC
Ctrl_691	1	Control	WTCCC
Ctrl_692	1	Control	WTCCC
Ctrl_693	1	Control	WTCCC
Ctrl_694	1	Control	WTCCC
Ctrl_695	1	Control	WTCCC
Ctrl_696	1	Control	WTCCC
Ctrl_697	1	Control	WTCCC
Ctrl_698	1	Control	WTCCC
Ctrl_699	1	Control	WTCCC

PD30758a	1	Control	WTCCC
Ctrl_700	1	Control	WTCCC
Ctrl_701	1	Control	WTCCC
Ctrl_702	1	Control	WTCCC
Ctrl_703	1	Control	WTCCC
Ctrl_704	1	Control	WTCCC
Ctrl_705	1	Control	WTCCC
Ctrl_706	1	Control	WTCCC
Ctrl_707	1	Control	WTCCC
Ctrl_708	1	Control	WTCCC
Ctrl_709	1	Control	WTCCC
Ctrl_710	1	Control	WTCCC
Ctrl_711	1	Control	WTCCC
Ctrl_712	1	Control	WTCCC
Ctrl_713	1	Control	WTCCC
Ctrl_714	1	Control	WTCCC
Ctrl_715	1	Control	WTCCC
Ctrl_716	1	Control	WTCCC
Ctrl_717	1	Control	WTCCC
Ctrl_718	1	Control	WTCCC
Ctrl_719	1	Control	WTCCC
Ctrl_720	1	Control	WTCCC
Ctrl_721	1	Control	WTCCC
Ctrl_722	1	Control	WTCCC
Ctrl_723	1	Control	WTCCC
Ctrl_724	1	Control	WTCCC
Ctrl_725	1	Control	WTCCC
Ctrl_726	1	Control	WTCCC
Ctrl_727	1	Control	WTCCC
Ctrl_728	1	Control	WTCCC
Ctrl_729	1	Control	WTCCC
Ctrl_730	1	Control	WTCCC
Ctrl_731	1	Control	WTCCC
Ctrl_732	1	Control	WTCCC
Ctrl_733	1	Control	WTCCC
Ctrl_734	1	Control	WTCCC
Ctrl_735	1	Control	WTCCC
Ctrl_736	1	Control	WTCCC
Ctrl_737	1	Control	WTCCC
Ctrl_738	1	Control	WTCCC

Ctrl_739	1	Control	WTCCC
Ctrl_740	1	Control	WTCCC
Ctrl_741	1	Control	WTCCC
Ctrl_742	1	Control	WTCCC
Ctrl_743	1	Control	WTCCC
Ctrl_744	1	Control	WTCCC
Ctrl_745	1	Control	WTCCC
Ctrl_746	1	Control	WTCCC
Ctrl_747	1	Control	WTCCC
Ctrl_748	1	Control	WTCCC
Ctrl_749	1	Control	WTCCC
Ctrl_750	1	Control	WTCCC
Ctrl_751	1	Control	WTCCC
Ctrl_752	1	Control	WTCCC
Ctrl_753	1	Control	WTCCC
Ctrl_754	1	Control	WTCCC
Ctrl_755	1	Control	WTCCC
Ctrl_756	1	Control	WTCCC
Ctrl_757	1	Control	WTCCC
Ctrl_758	1	Control	WTCCC
Ctrl_759	1	Control	WTCCC
Ctrl_760	1	Control	WTCCC
Ctrl_761	1	Control	WTCCC
Ctrl_762	1	Control	WTCCC
Ctrl_763	1	Control	WTCCC
Ctrl_764	1	Control	WTCCC
Ctrl_765	1	Control	WTCCC
Ctrl_766	1	Control	WTCCC
Ctrl_767	1	Control	WTCCC
Ctrl_768	1	Control	WTCCC
Ctrl_769	0.985301837	Control	WTCCC
Ctrl_770	1	Control	WTCCC
Ctrl_771	1	Control	WTCCC
Ctrl_772	1	Control	WTCCC
Ctrl_773	1	Control	WTCCC
Ctrl_774	1	Control	WTCCC
PD30741a	1	Control	WTCCC
Ctrl_775	1	Control	WTCCC
Ctrl_776	1	Control	WTCCC
Ctrl_777	1	Control	WTCCC

Ctrl_778	1	Control	WTCCC
Ctrl_779	1	Control	WTCCC
Ctrl_780	1	Control	WTCCC
Ctrl_781	1	Control	WTCCC
Ctrl_782	1	Control	WTCCC
Ctrl_783	1	Control	WTCCC
Ctrl_784	1	Control	WTCCC
Ctrl_785	1	Control	WTCCC
Ctrl_786	1	Control	WTCCC
Ctrl_787	1	Control	WTCCC
Ctrl_788	1	Control	WTCCC
Ctrl_789	1	Control	WTCCC
Ctrl_790	1	Control	WTCCC
Ctrl_791	1	Control	WTCCC
Ctrl_792	1	Control	WTCCC
Ctrl_793	1	Control	WTCCC
Ctrl_794	1	Control	WTCCC
Ctrl_795	1	Control	WTCCC
Ctrl_796	1	Control	WTCCC
Ctrl_797	1	Control	WTCCC
Ctrl_798	1	Control	WTCCC
Ctrl_799	1	Control	WTCCC
Ctrl_800	1	Control	WTCCC
Ctrl_801	1	Control	WTCCC
PD30744a	1	Control	WTCCC
Ctrl_802	1	Control	WTCCC
Ctrl_803	1	Control	WTCCC
Ctrl_804	1	Control	WTCCC
Ctrl_805	1	Control	WTCCC
Ctrl_806	1	Control	WTCCC
Ctrl_807	1	Control	WTCCC
Ctrl_808	1	Control	WTCCC
Ctrl_809	1	Control	WTCCC
Ctrl_810	1	Control	WTCCC
Ctrl_811	1	Control	WTCCC
Ctrl_812	1	Control	WTCCC
PD30737a	1	Control	WTCCC
Ctrl_813	1	Control	WTCCC
Ctrl_814	1	Control	WTCCC
Ctrl_815	1	Control	WTCCC

Ctrl_816	1	Control	WTCCC
Ctrl_817	1	Control	WTCCC
Ctrl_818	1	Control	WTCCC
Ctrl_819	0.943832021	Control	WTCCC
Ctrl_820	1	Control	WTCCC
Ctrl_821	1	Control	WTCCC
Ctrl_822	1	Control	WTCCC
Ctrl_823	1	Control	WTCCC
Ctrl_824	1	Control	WTCCC
Ctrl_825	1	Control	WTCCC
Ctrl_826	1	Control	WTCCC
PD30754a	1	Control	WTCCC
Ctrl_827	1	Control	WTCCC
Ctrl_828	1	Control	WTCCC
Ctrl_829	1	Control	WTCCC
Ctrl_830	1	Control	WTCCC
Ctrl_831	1	Control	WTCCC
Ctrl_832	1	Control	WTCCC
Ctrl_833	1	Control	WTCCC
Ctrl_834	1	Control	WTCCC
Ctrl_835	1	Control	WTCCC
Ctrl_836	1	Control	WTCCC
Ctrl_837	1	Control	WTCCC
Ctrl_838	1	Control	WTCCC
Ctrl_839	1	Control	WTCCC
Ctrl_840	1	Control	WTCCC
Ctrl_841	1	Control	WTCCC
Ctrl_842	1	Control	WTCCC
Ctrl_843	1	Control	WTCCC
Ctrl_844	1	Control	WTCCC
Ctrl_845	1	Control	WTCCC
Ctrl_846	1	Control	WTCCC
Ctrl_847	1	Control	WTCCC
Ctrl_848	1	Control	WTCCC
Ctrl_849	1	Control	WTCCC
Ctrl_850	1	Control	WTCCC
Ctrl_851	0.985301837	Control	WTCCC
Ctrl_852	1	Control	WTCCC
Ctrl_853	1	Control	WTCCC
Ctrl_854	1	Control	WTCCC

Ctrl_855	1	Control	WTCCC
Ctrl_856	1	Control	WTCCC
Ctrl_857	1	Control	WTCCC
PD30742a	0.991076115	Control	WTCCC
Ctrl_858	1	Control	WTCCC
Ctrl_859	1	Control	WTCCC
Ctrl_860	1	Control	WTCCC
Ctrl_861	1	Control	WTCCC
Ctrl_862	1	Control	WTCCC
Ctrl_863	1	Control	WTCCC
Ctrl_864	1	Control	WTCCC
PD30738a	0.999475066	Control	WTCCC
Ctrl_865	1	Control	WTCCC
Ctrl_866	1	Control	WTCCC
Ctrl_867	1	Control	WTCCC
Ctrl_868	1	Control	WTCCC
Ctrl_869	1	Control	WTCCC
Ctrl_870	1	Control	WTCCC
Ctrl_871	1	Control	WTCCC
Ctrl_872	1	Control	WTCCC
Ctrl_873	1	Control	WTCCC
Ctrl_874	1	Control	WTCCC
Ctrl_875	1	Control	WTCCC
Ctrl_876	1	Control	WTCCC
Ctrl_877	1	Control	WTCCC
Ctrl_878	0.995800525	Control	WTCCC
Ctrl_879	1	Control	WTCCC
Ctrl_880	1	Control	WTCCC
Ctrl_881	1	Control	WTCCC
Ctrl_882	1	Control	WTCCC
Ctrl_883	1	Control	WTCCC
Ctrl_884	1	Control	WTCCC
Ctrl_885	1	Control	WTCCC
Ctrl_886	1	Control	WTCCC
Ctrl_887	1	Control	WTCCC
Ctrl_888	1	Control	WTCCC
Ctrl_889	1	Control	WTCCC
Ctrl_890	1	Control	WTCCC
Ctrl_891	1	Control	WTCCC
Ctrl_892	1	Control	WTCCC

Ctrl_893	1	Control	WTCCC
Ctrl_894	1	Control	WTCCC
Ctrl_895	1	Control	WTCCC
Ctrl_896	1	Control	WTCCC
Ctrl_897	1	Control	WTCCC
Ctrl_898	1	Control	WTCCC
Ctrl_899	1	Control	WTCCC
Ctrl_900	1	Control	WTCCC
Ctrl_901	1	Control	WTCCC
Ctrl_902	1	Control	WTCCC
Ctrl_903	1	Control	WTCCC
Ctrl_904	1	Control	WTCCC
Ctrl_905	1	Control	WTCCC
Ctrl_906	1	Control	WTCCC
Ctrl_907	1	Control	WTCCC
Ctrl_908	1	Control	WTCCC
Ctrl_909	1	Control	WTCCC
Ctrl_910	1	Control	WTCCC
Ctrl_911	1	Control	WTCCC
Ctrl_912	1	Control	WTCCC
Ctrl_913	1	Control	WTCCC
Ctrl_914	1	Control	WTCCC
Ctrl_915	1	Control	WTCCC
Ctrl_916	1	Control	WTCCC
Ctrl_917	1	Control	WTCCC
Ctrl_918	1	Control	WTCCC
Ctrl_919	1	Control	WTCCC
Ctrl_920	1	Control	WTCCC
Ctrl_921	1	Control	WTCCC
Ctrl_922	1	Control	WTCCC
PD30755a	1	Control	WTCCC
Ctrl_923	1	Control	WTCCC
Ctrl_924	1	Control	WTCCC
PD30739a	0.994750656	Control	WTCCC
Ctrl_925	1	Control	WTCCC
Ctrl_926	1	Control	WTCCC
Ctrl_927	1	Control	WTCCC
Ctrl_928	1	Control	WTCCC
Ctrl_929	1	Control	WTCCC
Ctrl_930	1	Control	WTCCC

Ctrl_931	1	Control	WTCCC
Ctrl_932	1	Control	WTCCC
Ctrl_933	1	Control	WTCCC
Ctrl_934	1	Control	WTCCC
Ctrl_935	1	Control	WTCCC
Ctrl_936	1	Control	WTCCC
PD30745a	1	Control	WTCCC
Ctrl_937	1	Control	WTCCC
Ctrl_938	1	Control	WTCCC
Ctrl_939	1	Control	WTCCC
Ctrl_940	1	Control	WTCCC
Ctrl_941	1	Control	WTCCC
Ctrl_942	0.94855643	Control	WTCCC
Ctrl_943	0.94855643	Control	WTCCC
Ctrl_944	1	Control	WTCCC
Ctrl_945	1	Control	WTCCC
Ctrl_946	1	Control	WTCCC
Ctrl_947	1	Control	WTCCC
Ctrl_948	1	Control	WTCCC
Ctrl_949	0.94855643	Control	WTCCC
Ctrl_950	1	Control	WTCCC
Ctrl_951	0.94855643	Control	WTCCC
Ctrl_952	1	Control	WTCCC
Ctrl_953	1	Control	WTCCC
Ctrl_954	1	Control	WTCCC
Ctrl_955	1	Control	WTCCC
Ctrl_956	1	Control	WTCCC
Ctrl_957	0.94855643	Control	WTCCC
Ctrl_958	1	Control	WTCCC
Ctrl_959	1	Control	WTCCC
Ctrl_960	0.94855643	Control	WTCCC
Ctrl_961	1	Control	WTCCC
Ctrl_962	1	Control	WTCCC
Ctrl_963	1	Control	WTCCC
Ctrl_964	1	Control	WTCCC
Ctrl_965	1	Control	WTCCC
Ctrl_966	1	Control	WTCCC
Ctrl_967	1	Control	WTCCC
Ctrl_968	1	Control	WTCCC
Ctrl_969	1	Control	WTCCC

Ctrl_970	1	Control	WTCCC
Ctrl_971	1	Control	WTCCC
Ctrl_972	1	Control	WTCCC
Ctrl_973	1	Control	WTCCC
Ctrl_974	1	Control	WTCCC
Ctrl_975	1	Control	WTCCC
Ctrl_976	1	Control	WTCCC
Ctrl_977	1	Control	WTCCC
Ctrl_978	1	Control	WTCCC
Ctrl_979	1	Control	WTCCC
Ctrl_980	1	Control	WTCCC
Ctrl_981	1	Control	WTCCC
Ctrl_982	1	Control	WTCCC
Ctrl_983	1	Control	WTCCC
Ctrl_984	1	Control	WTCCC
Ctrl_985	0.94855643	Control	WTCCC
Ctrl_986	1	Control	WTCCC
Ctrl_987	0.94855643	Control	WTCCC
Ctrl_988	1	Control	WTCCC
Ctrl_989	0.94855643	Control	WTCCC
Ctrl_990	1	Control	WTCCC
Ctrl_991	1	Control	WTCCC
Ctrl_992	0.985301837	Control	WTCCC
Ctrl_993	1	Control	WTCCC
Ctrl_994	1	Control	WTCCC
Ctrl_995	1	Control	WTCCC
Ctrl_996	1	Control	WTCCC
Ctrl_997	1	Control	WTCCC
Ctrl_998	1	Control	WTCCC
Ctrl_999	1	Control	WTCCC
Ctrl_1000	0.94855643	Control	WTCCC
Ctrl_1001	0.94855643	Control	WTCCC
Ctrl_1002	1	Control	WTCCC
Ctrl_1003	1	Control	WTCCC
Ctrl_1004	1	Control	WTCCC
Ctrl_1005	1	Control	WTCCC
Ctrl_1006	1	Control	WTCCC
Ctrl_1007	1	Control	WTCCC
Ctrl_1008	1	Control	WTCCC
Ctrl_1009	1	Control	WTCCC

Ctrl_1010	1	Control	WTCCC
Ctrl_1011	1	Control	WTCCC
Ctrl_1012	1	Control	WTCCC
Ctrl_1013	1	Control	WTCCC
Ctrl_1014	1	Control	WTCCC
Ctrl_1015	1	Control	WTCCC
Ctrl_1016	1	Control	WTCCC
Ctrl_1017	1	Control	WTCCC
Ctrl_1018	1	Control	WTCCC
Ctrl_1019	1	Control	WTCCC
Ctrl_1020	1	Control	WTCCC
Ctrl_1021	0.94855643	Control	WTCCC
Ctrl_1022	1	Control	WTCCC
Ctrl_1023	1	Control	WTCCC
Ctrl_1024	1	Control	WTCCC
Ctrl_1025	1	Control	WTCCC
Ctrl_1026	1	Control	WTCCC
Ctrl_1027	1	Control	WTCCC
Ctrl_1028	1	Control	WTCCC
Ctrl_1029	1	Control	WTCCC
Ctrl_1030	1	Control	WTCCC
Ctrl_1031	1	Control	WTCCC
Ctrl_1032	1	Control	WTCCC
Ctrl_1033	1	Control	WTCCC
Ctrl_1034	1	Control	WTCCC
Ctrl_1035	1	Control	WTCCC
Ctrl_1036	1	Control	WTCCC
Ctrl_1037	1	Control	WTCCC
Ctrl_1038	1	Control	WTCCC
Ctrl_1039	1	Control	WTCCC
Ctrl_1040	1	Control	WTCCC
Ctrl_1041	1	Control	WTCCC
Ctrl_1042	1	Control	WTCCC
Ctrl_1043	1	Control	WTCCC
Ctrl_1044	1	Control	WTCCC
Ctrl_1045	1	Control	WTCCC
Ctrl_1046	1	Control	WTCCC
Ctrl_1047	1	Control	WTCCC
Ctrl_1048	1	Control	WTCCC
Ctrl_1049	1	Control	WTCCC

Ctrl_1050	1	Control	WTCCC
Ctrl_1051	1	Control	WTCCC
Ctrl_1052	1	Control	WTCCC
Ctrl_1053	1	Control	WTCCC
Ctrl_1054	1	Control	WTCCC
Ctrl_1055	1	Control	WTCCC
Ctrl_1056	1	Control	WTCCC
Ctrl_1057	1	Control	WTCCC
Ctrl_1058	1	Control	WTCCC
Ctrl_1059	1	Control	WTCCC
Ctrl_1060	1	Control	WTCCC
Ctrl_1061	1	Control	WTCCC
Ctrl_1062	1	Control	WTCCC
Ctrl_1063	1	Control	WTCCC
Ctrl_1064	1	Control	WTCCC
Ctrl_1065	1	Control	WTCCC
Ctrl_1066	1	Control	WTCCC
Ctrl_1067	1	Control	WTCCC
Ctrl_1068	1	Control	WTCCC
Ctrl_1069	1	Control	WTCCC
Ctrl_1070	1	Control	WTCCC
Ctrl_1071	1	Control	WTCCC
Ctrl_1072	1	Control	WTCCC
Ctrl_1073	1	Control	WTCCC
Ctrl_1074	1	Control	WTCCC
Ctrl_1075	1	Control	WTCCC
Ctrl_1076	1	Control	WTCCC
Ctrl_1077	1	Control	WTCCC
Ctrl_1078	1	Control	WTCCC
Ctrl_1079	1	Control	WTCCC
Ctrl_1080	1	Control	WTCCC
Ctrl_1081	1	Control	WTCCC
Ctrl_1082	1	Control	WTCCC
Ctrl_1083	1	Control	WTCCC
Ctrl_1084	1	Control	WTCCC
Ctrl_1085	1	Control	WTCCC
Ctrl_1086	1	Control	WTCCC
Ctrl_1087	1	Control	WTCCC
Ctrl_1088	1	Control	WTCCC
Ctrl_1089	1	Control	WTCCC

Ctrl_1090	1	Control	WTCCC
Ctrl_1091	1	Control	WTCCC
Ctrl_1092	1	Control	WTCCC
Ctrl_1093	1	Control	WTCCC
Ctrl_1094	0.94855643	Control	WTCCC
Ctrl_1095	1	Control	WTCCC
Ctrl_1096	1	Control	WTCCC
Ctrl_1097	1	Control	WTCCC
Ctrl_1098	1	Control	WTCCC
Ctrl_1099	1	Control	WTCCC
Ctrl_1100	0.94855643	Control	WTCCC
Ctrl_1101	1	Control	WTCCC
Ctrl_1102	1	Control	WTCCC
Ctrl_1103	1	Control	WTCCC
Ctrl_1104	1	Control	WTCCC
Ctrl_1105	1	Control	WTCCC
Ctrl_1106	1	Control	WTCCC
Ctrl_1107	1	Control	WTCCC
Ctrl_1108	1	Control	WTCCC
Ctrl_1109	1	Control	WTCCC
Ctrl_1110	1	Control	WTCCC
Ctrl_1111	1	Control	WTCCC
Ctrl_1112	1	Control	WTCCC
Ctrl_1113	1	Control	WTCCC
Ctrl_1114	1	Control	WTCCC
Ctrl_1115	1	Control	WTCCC
Ctrl_1116	1	Control	WTCCC
Ctrl_1117	1	Control	WTCCC
Ctrl_1118	1	Control	WTCCC
Ctrl_1119	1	Control	WTCCC
Ctrl_1120	1	Control	WTCCC
Ctrl_1121	1	Control	WTCCC
Ctrl_1122	1	Control	WTCCC
Ctrl_1123	1	Control	WTCCC
Ctrl_1124	1	Control	WTCCC
Ctrl_1125	1	Control	WTCCC
Ctrl_1126	1	Control	WTCCC
Ctrl_1127	0.94855643	Control	WTCCC
Ctrl_1128	0.94855643	Control	WTCCC
Ctrl_1129	1	Control	WTCCC

Ctrl_1130	1	Control	WTCCC
Ctrl_1131	1	Control	WTCCC
Ctrl_1132	1	Control	WTCCC
Ctrl_1133	1	Control	WTCCC
Ctrl_1134	1	Control	WTCCC
Ctrl_1135	1	Control	WTCCC
Ctrl_1136	1	Control	WTCCC
Ctrl_1137	1	Control	WTCCC
Ctrl_1138	1	Control	WTCCC
Ctrl_1139	1	Control	WTCCC
Ctrl_1140	1	Control	WTCCC
Ctrl_1141	1	Control	WTCCC
Ctrl_1142	1	Control	WTCCC
Ctrl_1143	1	Control	WTCCC
Ctrl_1144	1	Control	WTCCC
Ctrl_1145	1	Control	WTCCC
Ctrl_1146	1	Control	WTCCC
Ctrl_1147	1	Control	WTCCC
Ctrl_1148	1	Control	WTCCC
Ctrl_1149	1	Control	WTCCC
Ctrl_1150	1	Control	WTCCC
Ctrl_1151	1	Control	WTCCC
Ctrl_1152	1	Control	WTCCC
Ctrl_1153	1	Control	WTCCC
Ctrl_1154	1	Control	WTCCC
Ctrl_1155	1	Control	WTCCC
Ctrl_1156	1	Control	WTCCC
Ctrl_1157	1	Control	WTCCC
Ctrl_1158	1	Control	WTCCC
Ctrl_1159	1	Control	WTCCC
Ctrl_1160	1	Control	WTCCC
Ctrl_1161	1	Control	WTCCC
Ctrl_1162	1	Control	WTCCC
Ctrl_1163	1	Control	WTCCC
Ctrl_1164	1	Control	WTCCC
Ctrl_1165	1	Control	WTCCC
Ctrl_1166	1	Control	WTCCC
Ctrl_1167	1	Control	WTCCC
Ctrl_1168	1	Control	WTCCC
Ctrl_1169	1	Control	WTCCC

Ctrl_1170	1	Control	WTCCC
Ctrl_1171	1	Control	WTCCC
Ctrl_1172	1	Control	WTCCC
Ctrl_1173	1	Control	WTCCC
Ctrl_1174	1	Control	WTCCC
Ctrl_1175	1	Control	WTCCC
Ctrl_1176	1	Control	WTCCC
Ctrl_1177	1	Control	WTCCC
Ctrl_1178	1	Control	WTCCC
Ctrl_1179	1	Control	WTCCC
Ctrl_1180	1	Control	WTCCC
Ctrl_1181	1	Control	WTCCC
Ctrl_1182	1	Control	WTCCC
Ctrl_1183	1	Control	WTCCC
Ctrl_1184	0.999475066	Control	WTCCC
Ctrl_1185	1	Control	WTCCC
Ctrl_1186	1	Control	WTCCC
PD30747a	1	Control	WTCCC
Ctrl_1187	1	Control	WTCCC
Ctrl_1188	1	Control	WTCCC
Ctrl_1189	1	Control	WTCCC
Ctrl_1190	1	Control	WTCCC
Ctrl_1191	1	Control	WTCCC
Ctrl_1192	1	Control	WTCCC
Ctrl_1193	1	Control	WTCCC
Ctrl_1194	1	Control	WTCCC
Ctrl_1195	1	Control	WTCCC
Ctrl_1196	0.997375328	Control	WTCCC
Ctrl_1197	1	Control	WTCCC
Ctrl_1198	1	Control	WTCCC
Ctrl_1199	1	Control	WTCCC
Ctrl_1200	1	Control	WTCCC
Ctrl_1201	1	Control	WTCCC
Ctrl_1202	1	Control	WTCCC
Ctrl_1203	1	Control	WTCCC
Ctrl_1204	1	Control	WTCCC
Ctrl_1205	1	Control	WTCCC
Ctrl_1206	1	Control	WTCCC
Ctrl_1207	1	Control	WTCCC
Ctrl_1208	1	Control	WTCCC

Ctrl_1209	1	Control	WTCCC
Ctrl_1210	1	Control	WTCCC
Ctrl_1211	1	Control	WTCCC
Ctrl_1212	1	Control	WTCCC
Ctrl_1213	1	Control	WTCCC
Ctrl_1214	1	Control	WTCCC
Ctrl_1215	1	Control	WTCCC
Ctrl_1216	1	Control	WTCCC
Ctrl_1217	0.999475066	Control	WTCCC
Ctrl_1218	1	Control	WTCCC
Ctrl_1219	1	Control	WTCCC
PD30756a	1	Control	WTCCC
Ctrl_1220	1	Control	WTCCC
Ctrl_1221	1	Control	WTCCC
Ctrl_1222	1	Control	WTCCC
Ctrl_1223	0.94855643	Control	WTCCC
Ctrl_1224	0.94855643	Control	WTCCC
Ctrl_1225	1	Control	WTCCC
Ctrl_1226	0.94855643	Control	WTCCC
Ctrl_1227	0.94855643	Control	WTCCC
Ctrl_1228	0.94855643	Control	WTCCC
Ctrl_1229	1	Control	WTCCC
Ctrl_1230	1	Control	WTCCC
Ctrl_1231	1	Control	WTCCC
Ctrl_1232	1	Control	WTCCC
Ctrl_1233	1	Control	WTCCC
Ctrl_1234	1	Control	WTCCC
Ctrl_1235	1	Control	WTCCC
Ctrl_1236	1	Control	WTCCC
Ctrl_1237	1	Control	WTCCC
Ctrl_1238	1	Control	WTCCC
Ctrl_1239	1	Control	WTCCC
Ctrl_1240	1	Control	WTCCC
Ctrl_1241	1	Control	WTCCC
Ctrl_1242	1	Control	WTCCC
Ctrl_1243	1	Control	WTCCC
Ctrl_1244	1	Control	WTCCC
Ctrl_1245	1	Control	WTCCC
Ctrl_1246	1	Control	WTCCC
Ctrl_1247	1	Control	WTCCC

Ctrl_1248	1	Control	WTCCC
Ctrl_1249	1	Control	WTCCC
Ctrl_1250	0.94855643	Control	WTCCC
Ctrl_1251	1	Control	WTCCC
Ctrl_1252	0.94855643	Control	WTCCC
Ctrl_1253	1	Control	WTCCC
Ctrl_1254	0.94855643	Control	WTCCC
Ctrl_1255	1	Control	WTCCC
Ctrl_1256	1	Control	WTCCC
Ctrl_1257	1	Control	WTCCC
Ctrl_1258	1	Control	WTCCC
Ctrl_1259	0.952230971	Control	WTCCC
Ctrl_1260	0.94855643	Control	WTCCC
Ctrl_1261	1	Control	WTCCC
Ctrl_1262	0.94855643	Control	WTCCC
Ctrl_1263	1	Control	WTCCC
Ctrl_1264	1	Control	WTCCC
Ctrl_1265	1	Control	WTCCC
Ctrl_1266	0.94855643	Control	WTCCC
Ctrl_1267	0.94855643	Control	WTCCC
Ctrl_1268	1	Control	WTCCC
Ctrl_1269	1	Control	WTCCC
Ctrl_1270	1	Control	WTCCC
Ctrl_1271	0.94855643	Control	WTCCC
Ctrl_1272	0.94855643	Control	WTCCC
Ctrl_1273	0.94855643	Control	WTCCC
Ctrl_1274	1	Control	WTCCC
Ctrl_1275	1	Control	WTCCC
Ctrl_1276	1	Control	WTCCC
Ctrl_1277	0.94855643	Control	WTCCC
Ctrl_1278	1	Control	WTCCC
Ctrl_1279	1	Control	WTCCC
Ctrl_1280	1	Control	WTCCC
Ctrl_1281	1	Control	WTCCC
Ctrl_1282	1	Control	WTCCC
Ctrl_1283	1	Control	WTCCC
Ctrl_1284	1	Control	WTCCC
Ctrl_1285	1	Control	WTCCC
Ctrl_1286	1	Control	WTCCC
Ctrl_1287	1	Control	WTCCC

Ctrl_1288	1	Control	WTCCC
Ctrl_1289	1	Control	WTCCC
Ctrl_1290	1	Control	WTCCC
Ctrl_1291	1	Control	WTCCC
Ctrl_1292	1	Control	WTCCC
Ctrl_1293	1	Control	WTCCC
Ctrl_1294	1	Control	WTCCC
Ctrl_1295	1	Control	WTCCC
Ctrl_1296	1	Control	WTCCC
Ctrl_1297	1	Control	WTCCC
Ctrl_1298	1	Control	WTCCC
Ctrl_1299	1	Control	WTCCC
Ctrl_1300	1	Control	WTCCC
Ctrl_1301	1	Control	WTCCC
Ctrl_1302	1	Control	WTCCC
Ctrl_1303	1	Control	WTCCC
Ctrl_1304	1	Control	WTCCC
Ctrl_1305	1	Control	WTCCC
Ctrl_1306	1	Control	WTCCC
Ctrl_1307	1	Control	WTCCC
Ctrl_1308	1	Control	WTCCC
Ctrl_1309	1	Control	WTCCC
Ctrl_1310	1	Control	WTCCC
Ctrl_1311	1	Control	WTCCC
Ctrl_1312	1	Control	WTCCC
Ctrl_1313	1	Control	WTCCC
Ctrl_1314	1	Control	WTCCC
Ctrl_1315	1	Control	WTCCC
Ctrl_1316	1	Control	WTCCC
Ctrl_1317	1	Control	WTCCC
Ctrl_1318	1	Control	WTCCC
Ctrl_1319	1	Control	WTCCC
Ctrl_1320	1	Control	WTCCC
Ctrl_1321	1	Control	WTCCC
Ctrl_1322	1	Control	WTCCC
Ctrl_1323	1	Control	WTCCC
Ctrl_1324	1	Control	WTCCC
Ctrl_1325	1	Control	WTCCC
Ctrl_1326	1	Control	WTCCC
Ctrl_1327	1	Control	WTCCC

Ctrl_1328	1	Control	WTCCC
Ctrl_1329	1	Control	WTCCC
Ctrl_1330	1	Control	WTCCC
Ctrl_1331	0.94855643	Control	WTCCC
Ctrl_1332	0.94855643	Control	WTCCC
Ctrl_1333	0.948031496	Control	WTCCC
Ctrl_1334	1	Control	WTCCC
Ctrl_1335	1	Control	WTCCC
Ctrl_1336	1	Control	WTCCC
Ctrl_1337	0.94855643	Control	WTCCC
Ctrl_1338	1	Control	WTCCC
Ctrl_1339	1	Control	WTCCC
Ctrl_1340	1	Control	WTCCC
Ctrl_1341	1	Control	WTCCC
Ctrl_1342	1	Control	WTCCC
Ctrl_1343	0.94855643	Control	WTCCC
Ctrl_1344	1	Control	WTCCC
Ctrl_1345	1	Control	WTCCC
Ctrl_1346	1	Control	WTCCC
Ctrl_1347	1	Control	WTCCC
Ctrl_1348	1	Control	WTCCC
Ctrl_1349	1	Control	WTCCC
Ctrl_1350	1	Control	WTCCC
Ctrl_1351	1	Control	WTCCC
Ctrl_1352	0.94855643	Control	WTCCC
Ctrl_1353	1	Control	WTCCC
Ctrl_1354	0.94855643	Control	WTCCC
Ctrl_1355	1	Control	WTCCC
Ctrl_1356	1	Control	WTCCC
Ctrl_1357	1	Control	WTCCC
Ctrl_1358	1	Control	WTCCC
Ctrl_1359	1	Control	WTCCC
Ctrl_1360	1	Control	WTCCC
Ctrl_1361	1	Control	WTCCC
Ctrl_1362	1	Control	WTCCC
Ctrl_1363	1	Control	WTCCC
Ctrl_1364	1	Control	WTCCC
Ctrl_1365	1	Control	WTCCC
Ctrl_1366	1	Control	WTCCC
Ctrl_1367	1	Control	WTCCC

Ctrl_1368	1	Control	WTCCC
Ctrl_1369	1	Control	WTCCC
Ctrl_1370	1	Control	WTCCC
Ctrl_1371	1	Control	WTCCC
Ctrl_1372	1	Control	WTCCC
Ctrl_1373	1	Control	WTCCC
Ctrl_1374	1	Control	WTCCC
Ctrl_1375	1	Control	WTCCC
Ctrl_1376	1	Control	WTCCC
Ctrl_1377	1	Control	WTCCC
Ctrl_1378	1	Control	WTCCC
Ctrl_1379	1	Control	WTCCC
Ctrl_1380	1	Control	WTCCC
Ctrl_1381	1	Control	WTCCC
Ctrl_1382	1	Control	WTCCC
Ctrl_1383	1	Control	WTCCC
Ctrl_1384	1	Control	WTCCC
Ctrl_1385	1	Control	WTCCC
Ctrl_1386	1	Control	WTCCC
Ctrl_1387	1	Control	WTCCC
Ctrl_1388	1	Control	WTCCC
Ctrl_1389	1	Control	WTCCC
Ctrl_1390	1	Control	WTCCC
Ctrl_1391	1	Control	WTCCC
Ctrl_1392	1	Control	WTCCC
Ctrl_1393	1	Control	WTCCC
Ctrl_1394	1	Control	WTCCC
Ctrl_1395	1	Control	WTCCC
Ctrl_1396	1	Control	WTCCC
Ctrl_1397	1	Control	WTCCC
Ctrl_1398	1	Control	WTCCC
Ctrl_1399	1	Control	WTCCC
Ctrl_1400	1	Control	WTCCC
Ctrl_1401	1	Control	WTCCC
Ctrl_1402	1	Control	WTCCC
Ctrl_1403	1	Control	WTCCC
Ctrl_1404	1	Control	WTCCC
Ctrl_1405	1	Control	WTCCC
Case_1	1	Case	Leeds
Case_2	1	Case	Leeds

Case_3	1	Case	Leeds
Case_4	1	Case	Leeds
Case_5	1	Case	Leeds
Case_6	1	Case	Leeds
Case_7	1	Case	Leeds
Case_8	1	Case	Leeds
Case_9	1	Case	Leeds
PD30576a	0.988976378	Case	Leeds
Case_10	1	Case	Leeds
Case_11	1	Case	Leeds
Case_12	1	Case	Leeds
Case_13	1	Case	Leeds
Case_14	1	Case	Leeds
Case_15	1	Case	Leeds
Case_16	1	Case	Leeds
Case_17	1	Case	Leeds
Case_18	1	Case	Leeds
Case_19	1	Case	Leeds
PD30577a	0.985826772	Case	Leeds
Case_20	1	Case	Leeds
Case_21	1	Case	Leeds
Case_22	1	Case	Leeds
Case_23	1	Case	Leeds
Case_24	1	Case	Leeds
Case_25	1	Case	Leeds
Case_26	1	Case	Leeds
Case_27	1	Case	Leeds
Case_28	1	Case	Leeds
Case_29	1	Case	Leeds
Case_30	1	Case	Leeds
Case_31	1	Case	Leeds
Case_32	1	Case	Leeds
Case_33	1	Case	Leeds
Case_34	1	Case	Leeds
Case_35	1	Case	Leeds
Case_36	1	Case	Leeds
Case_37	1	Case	Leeds
Case_38	1	Case	Leeds
Case_39	1	Case	Leeds
Case_40	1	Case	Leeds

Case_41	1	Case	Leeds
Case_42	1	Case	Leeds
Case_43	1	Case	Leeds
PD30578a	1	Case	Leeds
Case_44	1	Case	Leeds
Case_45	1	Case	Leeds
Case_46	1	Case	Leeds
Case_47	1	Case	Leeds
Case_48	1	Case	Leeds
Case_49	1	Case	Leeds
Case_50	1	Case	Leeds
Case_51	1	Case	Leeds
Case_52	1	Case	Leeds
Case_53	1	Case	Leeds
Case_54	1	Case	Leeds
Case_55	1	Case	Leeds
Case_56	1	Case	Leeds
Case_57	1	Case	Leeds
Case_58	1	Case	Leeds
Case_59	1	Case	Leeds
Case_60	1	Case	Leeds
Case_61	1	Case	Leeds
Case_62	1	Case	Leeds
Case_63	1	Case	Leeds
Case_64	1	Case	Leeds
Case_65	1	Case	Leeds
Case_66	1	Case	Leeds
Case_67	1	Case	Leeds
Case_68	1	Case	Leeds
Case_69	1	Case	Leeds
Case_70	1	Case	Leeds
Case_71	1	Case	Leeds
Case_72	1	Case	Leeds
Case_73	1	Case	Leeds
Case_74	1	Case	Leeds
Case_75	1	Case	Leeds
Case_76	1	Case	Leeds
Case_77	1	Case	Leeds
Case_78	1	Case	Leeds
Case_79	1	Case	Leeds

Case_80	1	Case	Leeds
Case_81	1	Case	Leeds
Case_82	1	Case	Leeds
Case_83	1	Case	Leeds
Case_84	1	Case	Leeds
Case_85	1	Case	Leeds
Case_86	1	Case	Leeds
Case_87	1	Case	Leeds
Case_88	1	Case	Leeds
Case_89	1	Case	Leeds
Case_90	0.987926509	Case	Leeds
Case_91	1	Case	Leeds
Case_92	1	Case	Leeds
Case_93	1	Case	Leeds
Case_94	1	Case	Leeds
Case_95	1	Case	Leeds
Case_96	1	Case	Leeds
Case_97	1	Case	Leeds
Case_98	1	Case	Leeds
Case_99	1	Case	Leeds
Case_100	1	Case	Leeds
Case_101	1	Case	Leeds
Case_102	1	Case	Leeds
Case_103	1	Case	Leeds
Case_104	1	Case	Leeds
Case_105	1	Case	Leeds
Case_106	1	Case	Leeds
Case_107	1	Case	Leeds
Case_108	1	Case	Leeds
Case_109	1	Case	Leeds
Case_110	1	Case	Leeds
Case_111	1	Case	Leeds
Case_112	1	Case	Leeds
Case_113	1	Case	Leeds
Case_114	1	Case	Leeds
Case_115	1	Case	Leeds
Case_116	1	Case	Leeds
Case_117	1	Case	Leeds
Case_118	1	Case	Leeds
Case_119	1	Case	Leeds

Case_120	1	Case	Leeds
Case_121	1	Case	Leeds
Case_122	1	Case	Leeds
Case_123	1	Case	Leeds
Case_124	1	Case	Leeds
Case_125	1	Case	Leeds
Case_126	1	Case	Leeds
Case_127	0.996325459	Case	Leeds
Case_128	1	Case	Leeds
Case_129	1	Case	Leeds
Case_130	1	Case	Leeds
Case_131	1	Case	Leeds
Case_132	1	Case	Leeds
Case_133	1	Case	Leeds
Case_134	1	Case	Leeds
Case_135	1	Case	Leeds
Case_136	1	Case	Leeds
Case_137	1	Case	Leeds
Case_138	1	Case	Leeds
Case_139	1	Case	Leeds
Case_140	1	Case	Leeds
Case_141	1	Case	Leeds
Case_142	1	Case	Leeds
Case_143	1	Case	Leeds
Case_144	1	Case	Leeds
PD30580a	1	Case	Leeds
Case_145	1	Case	Leeds
Case_146	1	Case	Leeds
Case_147	1	Case	Leeds
Case_148	1	Case	Leeds
PD30581a	1	Case	Leeds
Case_149	1	Case	Leeds
Case_150	1	Case	Leeds
Case_151	1	Case	Leeds
Case_152	1	Case	Leeds
Case_153	1	Case	Leeds
Case_154	1	Case	Leeds
Case_155	1	Case	Leeds
Case_156	1	Case	Leeds
Case_157	1	Case	Leeds

Case_158	0.992125984	Case	Leeds
Case_159	1	Case	Leeds
Case_160	1	Case	Leeds
Case_161	1	Case	Leeds
Case_162	1	Case	Leeds
Case_163	1	Case	Leeds
Case_164	1	Case	Leeds
Case_165	1	Case	Leeds
Case_166	1	Case	Leeds
Case_167	1	Case	Leeds
Case_168	1	Case	Leeds
Case_169	1	Case	Leeds
Case_170	1	Case	Leeds
Case_171	1	Case	Leeds
Case_172	1	Case	Leeds
Case_173	1	Case	Leeds
Case_174	1	Case	Leeds
Case_175	1	Case	Leeds
Case_176	1	Case	Leeds
Case_177	1	Case	Leeds
Case_178	1	Case	Leeds
Case_179	1	Case	Leeds
Case_180	1	Case	Leeds
Case_181	1	Case	Leeds
Case_182	1	Case	Leeds
Case_183	1	Case	Leeds
Case_184	1	Case	Leeds
Case_185	1	Case	Leeds
Case_186	1	Case	Leeds
Case_187	1	Case	Leeds
Case_188	1	Case	Leeds
Case_189	1	Case	Leeds
Case_190	1	Case	Leeds
Case_191	1	Case	Leeds
Case_192	1	Case	Leeds
Case_193	1	Case	Leeds
Case_194	1	Case	Leeds
Case_195	1	Case	Leeds
Case_196	1	Case	Leeds
Case_197	1	Case	Leeds

Case_198	1	Case	Leeds
Case_199	1	Case	Leeds
Case_200	1	Case	Leeds
Case_201	1	Case	Leeds
Case_202	1	Case	Leeds
Case_203	1	Case	Leeds
Case_204	1	Case	Leeds
Case_205	1	Case	Leeds
Case_206	1	Case	Leeds
Case_207	1	Case	Leeds
Case_208	1	Case	Leeds
Case_209	1	Case	Leeds
Case_210	1	Case	Leeds
Case_211	1	Case	Leeds
Case_212	1	Case	Leeds
Case_213	1	Case	Leeds
Case_214	1	Case	Leeds
Case_215	1	Case	Leeds
Case_216	1	Case	Leeds
Case_217	1	Case	Leeds
Case_218	1	Case	Leeds
Case_219	1	Case	Leeds
Case_220	1	Case	Leeds
Case_221	1	Case	Leeds
Case_222	1	Case	Leeds
Case_223	1	Case	Leeds
Case_224	1	Case	Leeds
Case_225	1	Case	Leeds
Case_226	1	Case	Leeds
Case_227	1	Case	Leeds
Case_228	1	Case	Leeds
Case_229	1	Case	Leeds
Case_230	1	Case	Leeds
Case_231	1	Case	Leeds
Case_232	1	Case	Leeds
Case_233	1	Case	Leeds
Case_234	1	Case	Leeds
Case_235	1	Case	Leeds
Case_236	1	Case	Leeds
Case_237	1	Case	Leeds

Case_238	1	Case	Leeds
Case_239	1	Case	Leeds
Case_240	1	Case	Leeds
Case_241	1	Case	Leeds
Case_242	1	Case	Leeds
Case_243	1	Case	Leeds
Case_244	1	Case	Leeds
Case_245	1	Case	Leeds
Case_246	1	Case	Leeds
Case_247	1	Case	Leeds
Case_248	1	Case	Leeds
Case_249	1	Case	Leeds
Case_250	1	Case	Leeds
Case_251	1	Case	Leeds
Case_252	1	Case	Leeds
Case_253	1	Case	Leeds
Case_254	1	Case	Leeds
Case_255	1	Case	Leeds
Case_256	1	Case	Leeds
Case_257	1	Case	Leeds
Case_258	1	Case	Leeds
Case_259	1	Case	Leeds
Case_260	1	Case	Leeds
Case_261	1	Case	Leeds
Case_262	1	Case	Leeds
Case_263	1	Case	Leeds
Case_264	1	Case	Leeds
Case_265	1	Case	Leeds
Case_266	1	Case	Leeds
Case_267	1	Case	Leeds
Case_268	1	Case	Leeds
Case_269	1	Case	Leeds
Case_270	1	Case	Leeds
Case_271	1	Case	Leeds
Case_272	1	Case	Leeds
Case_273	1	Case	Leeds
Case_274	1	Case	Leeds
Case_275	1	Case	Leeds
Case_276	1	Case	Leeds
Case_277	1	Case	Leeds

Case_278	1	Case	Leeds
Case_279	1	Case	Leeds
Case_280	1	Case	Leeds
PD30582a	1	Case	Leeds
Case_281	1	Case	Leeds
Case_282	1	Case	Leeds
Case_283	1	Case	Leeds
Case_284	1	Case	Leeds
Case_285	1	Case	Leeds
Case_286	1	Case	Leeds
Case_287	1	Case	Leeds
Case_288	1	Case	Leeds
Case_289	1	Case	Leeds
Case_290	1	Case	Leeds
Case_291	1	Case	Leeds
Case_292	1	Case	Leeds
Case_293	1	Case	Leeds
Case_294	1	Case	Leeds
Case_295	1	Case	Leeds
Case_296	1	Case	Leeds
Case_297	1	Case	Leeds
Case_298	1	Case	Leeds
Case_299	1	Case	Leeds
Case_300	1	Case	Leeds
Case_301	1	Case	Leeds
Case_302	1	Case	Leeds
Case_303	1	Case	Leeds
Case_304	1	Case	Leeds
Case_305	1	Case	Leeds
Case_306	1	Case	Leeds
Case_307	1	Case	Leeds
Case_308	1	Case	Leeds
Case_309	1	Case	Leeds
Case_310	1	Case	Leeds
Case_311	1	Case	Leeds
Case_312	1	Case	Leeds
Case_313	1	Case	Leeds
Case_314	1	Case	Leeds
Case_315	1	Case	Leeds
Case_316	1	Case	Leeds

Case_317	1	Case	Leeds
Case_318	1	Case	Leeds
Case_319	1	Case	Leeds
Case_320	1	Case	Leeds
Case_321	1	Case	Leeds
Case_322	1	Case	Leeds
Case_323	1	Case	Leeds
Case_324	1	Case	Leeds
Ctrl_1406	1	Control	Leeds
Case_325	1	Case	Leeds
Case_326	1	Case	Leeds
Case_327	1	Case	Leeds
Case_328	0.999475066	Case	Leeds
Case_329	1	Case	Leeds
Case_330	1	Case	Leeds
Case_331	1	Case	Leeds
Case_332	1	Case	Leeds
Case_333	1	Case	Leeds
Case_334	1	Case	Leeds
Case_335	1	Case	Leeds
Case_336	1	Case	Leeds
Case_337	1	Case	Leeds
Case_338	1	Case	Leeds
Case_339	1	Case	Leeds
PD30583a	0.995275591	Case	Leeds
Case_340	1	Case	Leeds
Case_341	1	Case	Leeds
Case_342	1	Case	Leeds
Case_343	1	Case	Leeds
Case_344	1	Case	Leeds
Case_345	1	Case	Leeds
Case_346	1	Case	Leeds
Case_347	1	Case	Leeds
Case_348	1	Case	Leeds
Case_349	1	Case	Leeds
Case_350	1	Case	Leeds
Case_351	1	Case	Leeds
Case_352	1	Case	Leeds
Case_353	1	Case	Leeds
Case_354	1	Case	Leeds

Case_355	1	Case	Leeds
Case_356	1	Case	Leeds
Case_357	1	Case	Leeds
Case_358	1	Case	Leeds
Case_359	1	Case	Leeds
Case_360	1	Case	Leeds
Case_361	1	Case	Leeds
Case_362	1	Case	Leeds
Case_363	1	Case	Leeds
Case_364	1	Case	Leeds
Case_365	1	Case	Leeds
Case_366	1	Case	Leeds
Case_367	1	Case	Leeds
Case_368	1	Case	Leeds
Case_369	1	Case	Leeds
Case_370	1	Case	Leeds
Case_371	1	Case	Leeds
Case_372	1	Case	Leeds
Case_373	1	Case	Leeds
Case_374	1	Case	Leeds
Case_375	1	Case	Leeds
Case_376	1	Case	Leeds
Case_377	1	Case	Leeds
Case_378	1	Case	Leeds
Case_379	1	Case	Leeds
Case_380	1	Case	Leeds
Case_381	1	Case	Leeds
Case_382	1	Case	Leeds
Case_383	1	Case	Leeds
Case_384	1	Case	Leeds
Case_385	1	Case	Leeds
Case_386	1	Case	Leeds
Case_387	1	Case	Leeds
Case_388	1	Case	Leeds
Case_389	1	Case	Leeds
Case_390	1	Case	Leeds
Case_391	1	Case	Leeds
Case_392	1	Case	Leeds
Case_393	1	Case	Leeds
Case_394	1	Case	Leeds

Case_395	1	Case	Leeds
Case_396	1	Case	Leeds
Case_397	1	Case	Leeds
Case_398	1	Case	Leeds
Case_399	1	Case	Leeds
Case_400	1	Case	Leeds
Case_401	1	Case	Leeds
Case_402	1	Case	Leeds
Case_403	1	Case	Leeds
Case_404	1	Case	Leeds
Case_405	1	Case	Leeds
Case_406	1	Case	Leeds
Case_407	1	Case	Leeds
Case_408	1	Case	Leeds
Case_409	1	Case	Leeds
Case_410	1	Case	Leeds
Case_411	1	Case	Leeds
Case_412	0.995275591	Case	Leeds
Case_413	1	Case	Leeds
Case_414	1	Case	Leeds
Case_415	1	Case	Leeds
Case_416	1	Case	Leeds
PD30584a	1	Case	Leeds
Case_417	1	Case	Leeds
Case_418	1	Case	Leeds
Case_419	1	Case	Leeds
Case_420	1	Case	Leeds
Case_421	1	Case	Leeds
Case_422	1	Case	Leeds
Case_423	1	Case	Leeds
Case_424	1	Case	Leeds
Case_425	1	Case	Leeds
Case_426	1	Case	Leeds
Case_427	1	Case	Leeds
Case_428	1	Case	Leeds
Case_429	1	Case	Leeds
Case_430	1	Case	Leeds
Case_431	1	Case	Leeds
PD30585a	1	Case	Leeds
Case_432	1	Case	Leeds

Case_433	1	Case	Leeds
Case_434	1	Case	Leeds
Case_435	1	Case	Leeds
Case_436	1	Case	Leeds
Case_437	1	Case	Leeds
Case_438	1	Case	Leeds
Case_439	1	Case	Leeds
Case_440	1	Case	Leeds
Case_441	1	Case	Leeds
Case_442	1	Case	Leeds
Case_443	1	Case	Leeds
PD30586a	1	Case	Leeds
Case_444	1	Case	Leeds
Case_445	1	Case	Leeds
Case_446	1	Case	Leeds
Case_447	1	Case	Leeds
PD30587a	1	Case	Leeds
Case_448	1	Case	Leeds
Case_449	1	Case	Leeds
Case_450	1	Case	Leeds
Case_451	1	Case	Leeds
Case_452	1	Case	Leeds
Case_453	1	Case	Leeds
Case_454	1	Case	Leeds
Case_455	1	Case	Leeds
Case_456	1	Case	Leeds
Case_457	1	Case	Leeds
Case_458	1	Case	Leeds
Case_459	1	Case	Leeds
Case_460	1	Case	Leeds
Case_461	1	Case	Leeds
Case_462	1	Case	Leeds
Case_463	1	Case	Leeds
Case_464	1	Case	Leeds
Case_465	1	Case	Leeds
Case_466	1	Case	Leeds
Case_467	1	Case	Leeds
Case_468	1	Case	Leeds
Case_469	1	Case	Leeds
Case_470	1	Case	Leeds

Case_471	1	Case	Leeds
Case_472	1	Case	Leeds
Case_473	1	Case	Leeds
Case_474	1	Case	Leeds
Case_475	1	Case	Leeds
Case_476	0.951181102	Case	Leeds
Case_477	1	Case	Leeds
Case_478	1	Case	Leeds
Case_479	1	Case	Leeds
Case_480	1	Case	Leeds
Case_481	1	Case	Leeds
Case_482	1	Case	Leeds
Case_483	1	Case	Leeds
Case_484	1	Case	Leeds
Case_485	1	Case	Leeds
Case_486	1	Case	Leeds
Case_487	1	Case	Leeds
Case_488	1	Case	Leeds
Case_489	1	Case	Leeds
Case_490	1	Case	Leeds
Case_491	1	Case	Leeds
Case_492	1	Case	Leeds
Case_493	1	Case	Leeds
Case_494	1	Case	Leeds
Case_495	1	Case	Leeds
Case_496	1	Case	Leeds
Case_497	1	Case	Leeds
Case_498	1	Case	Leeds
Case_499	1	Case	Leeds
Case_500	1	Case	Leeds
Case_501	1	Case	Leeds
Case_502	1	Case	Leeds
Case_503	1	Case	Leeds
Case_504	1	Case	Leeds
Case_505	1	Case	Leeds
Case_506	1	Case	Leeds
Case_507	1	Case	Leeds
Case_508	1	Case	Leeds
Case_509	1	Case	Leeds
Case_510	1	Case	Leeds

Case_511	1	Case	Leeds
Case_512	1	Case	Leeds
Case_513	1	Case	Leeds
Case_514	1	Case	Leeds
Case_515	1	Case	Leeds
Case_516	1	Case	Leeds
Case_517	1	Case	Leeds
Ctrl_1407	1	Control	Leeds
Ctrl_1408	1	Control	Leeds
Case_518	1	Case	Leeds
Case_519	1	Case	Leeds
Case_520	1	Case	Leeds
Case_521	1	Case	Leeds
Case_522	1	Case	Leeds
Case_523	1	Case	Leeds
Case_524	1	Case	Leeds
Case_525	1	Case	Leeds
Case_526	1	Case	Leeds
Case_527	1	Case	Leeds
Ctrl_1409	1	Control	Leeds
Case_528	1	Case	Leeds
Case_529	1	Case	Leeds
Case_530	1	Case	Leeds
Case_531	1	Case	Leeds
Case_532	1	Case	Leeds
Case_533	1	Case	Leeds
Case_534	1	Case	Leeds
Case_535	1	Case	Leeds
Case_536	1	Case	Leeds
Case_537	1	Case	Leeds
Case_538	1	Case	Leeds
Case_539	1	Case	Leeds
Case_540	1	Case	Leeds
Case_541	1	Case	Leeds
Case_542	1	Case	Leeds
Case_543	1	Case	Leeds
Case_544	1	Case	Leeds
Case_545	1	Case	Leeds
Case_546	1	Case	Leeds
Case_547	1	Case	Leeds

Case_548	1	Case	Leeds
Case_549	1	Case	Leeds
Case_550	1	Case	Leeds
Case_551	1	Case	Leeds
Case_552	1	Case	Leeds
Case_553	1	Case	Leeds
PD30588a	1	Case	Leeds
Case_554	1	Case	Leeds
Case_555	1	Case	Leeds
Case_556	1	Case	Leeds
Case_557	1	Case	Leeds
Case_558	1	Case	Leeds
Case_559	1	Case	Leeds
Case_560	1	Case	Leeds
Case_561	1	Case	Leeds
Case_562	1	Case	Leeds
Case_563	1	Case	Leeds
Case_564	1	Case	Leeds
Case_565	1	Case	Leeds
Case_566	1	Case	Leeds
Case_567	1	Case	Leeds
Case_568	1	Case	Leeds
Case_569	1	Case	Leeds
Case_570	1	Case	Leeds
Case_571	1	Case	Leeds
Case_572	1	Case	Leeds
Case_573	1	Case	Leeds
Case_574	1	Case	Leeds
Case_575	1	Case	Leeds
Case_576	1	Case	Leeds
Case_577	1	Case	Leeds
Case_578	1	Case	Leeds
Case_579	1	Case	Leeds
Case_580	1	Case	Leeds
Case_581	1	Case	Leeds
Case_582	1	Case	Leeds
Case_583	1	Case	Leeds
Case_584	1	Case	Leeds
Case_585	1	Case	Leeds
Case_586	1	Case	Leeds

Case_587	1	Case	Leeds
Case_588	1	Case	Leeds
Case_589	1	Case	Leeds
Case_590	1	Case	Leeds
Case_591	1	Case	Leeds
Case_592	1	Case	Leeds
Case_593	1	Case	Leeds
Case_594	1	Case	Leeds
Case_595	1	Case	Leeds
Ctrl_1410	1	Control	Leeds
Case_596	1	Case	Leeds
Case_597	1	Case	Leeds
Case_598	1	Case	Leeds
Case_599	1	Case	Leeds
Case_600	1	Case	Leeds
Case_601	1	Case	Leeds
Case_602	1	Case	Leeds
Case_603	1	Case	Leeds
Case_604	1	Case	Leeds
Case_605	1	Case	Leeds
Case_606	1	Case	Leeds
Case_607	1	Case	Leeds
Case_608	1	Case	Leeds
Case_609	1	Case	Leeds
Case_610	1	Case	Leeds
Case_611	1	Case	Leeds
Case_612	1	Case	Leeds
Case_613	1	Case	Leeds
Case_614	1	Case	Leeds
Case_615	1	Case	Leeds
Case_616	1	Case	Leeds
Case_617	1	Case	Leeds
Case_618	1	Case	Leeds
Case_619	1	Case	Leeds
Case_620	1	Case	Leeds
Case_621	1	Case	Leeds
Case_622	1	Case	Leeds
Case_623	1	Case	Leeds
Case_624	1	Case	Leeds
Case_625	1	Case	Leeds

Case_626	1	Case	Leeds
Case_627	1	Case	Leeds
Case_628	1	Case	Leeds
Case_629	1	Case	Leeds
Case_630	1	Case	Leeds
Case_631	1	Case	Leeds
Case_632	1	Case	Leeds
Case_633	1	Case	Leeds
Case_634	1	Case	Leeds
Case_635	1	Case	Leeds
Case_636	1	Case	Leeds
Case_637	1	Case	Leeds
Case_638	1	Case	Leeds
Case_639	1	Case	Leeds
Case_640	1	Case	Leeds
Case_641	1	Case	Leeds
Case_642	1	Case	Leeds
Ctrl_1411	1	Control	Leeds
Case_643	1	Case	Leeds
Case_644	1	Case	Leeds
Case_645	1	Case	Leeds
Case_646	1	Case	Leeds
Ctrl_1412	1	Control	Leeds
Ctrl_1413	1	Control	Leeds
Ctrl_1414	1	Control	Leeds
Ctrl_1415	1	Control	Leeds
Ctrl_1416	1	Control	Leeds
Case_647	1	Case	Leeds
Case_648	1	Case	Leeds
Ctrl_1417	1	Control	Leeds
Case_649	0.955905512	Case	Leeds
Case_650	1	Case	Leeds
Case_651	1	Case	Leeds
Case_652	1	Case	Leeds
Case_653	1	Case	Leeds
Case_654	1	Case	Leeds
Case_655	1	Case	Leeds
Ctrl_1418	1	Control	Leeds
Case_656	1	Case	Leeds
Case_657	1	Case	Leeds

Case_658	1	Case	Leeds
PD30589a	1	Case	Leeds
PD30590a	1	Case	Leeds
Case_659	1	Case	Leeds
Case_660	1	Case	Leeds
Ctrl_1419	1	Control	Leeds
Ctrl_1420	1	Control	Leeds
Case_661	1	Case	Leeds
Ctrl_1421	1	Control	Leeds
Case_662	1	Case	Leeds
Ctrl_1422	1	Control	Leeds
Case_663	1	Case	Leeds
Case_664	1	Case	Leeds
Ctrl_1423	1	Control	Leeds
Case_665	1	Case	Leeds
Case_666	1	Case	Leeds
Case_667	1	Case	Leeds
Case_668	1	Case	Leeds
Case_669	1	Case	Leeds
Ctrl_1424	1	Control	Leeds
Case_670	1	Case	Leeds
Ctrl_1425	1	Control	Leeds
Case_671	1	Case	Leeds
Ctrl_1426	1	Control	Leeds
Case_672	1	Case	Leeds
Case_673	1	Case	Leeds
Case_674	1	Case	Leeds
Case_675	1	Case	Leeds
Case_676	1	Case	Leeds
Case_677	1	Case	Leeds
Case_678	1	Case	Leeds
Case_679	1	Case	Leeds
Case_680	1	Case	Leeds
Case_681	1	Case	Leeds
Case_682	1	Case	Leeds
Case_683	1	Case	Leeds
Case_684	1	Case	Leeds
Case_685	1	Case	Leeds
Ctrl_1427	1	Control	Leeds
Ctrl_1428	1	Control	Leeds

Ctrl_1429	1	Control	Leeds
Case_686	1	Case	Leeds
Ctrl_1430	1	Control	Leeds
Case_687	1	Case	Leeds
Ctrl_1431	1	Control	Leeds
Ctrl_1432	1	Control	Leeds
Case_688	1	Case	Leeds
Case_689	1	Case	Leeds
Ctrl_1433	1	Control	Leeds
Ctrl_1434	1	Control	Leeds
Ctrl_1435	1	Control	Leeds
Ctrl_1436	1	Control	Leeds
Ctrl_1437	1	Control	Leeds
Ctrl_1438	1	Control	Leeds
Ctrl_1439	1	Control	Leeds
Case_690	1	Case	Leeds
Case_691	1	Case	Leeds
Ctrl_1440	1	Control	Leeds
Ctrl_1441	1	Control	Leeds
Ctrl_1442	1	Control	Leeds
Case_692	1	Case	Leeds
Ctrl_1443	1	Control	Leeds
Case_693	1	Case	Leeds
Ctrl_1444	1	Control	Leeds
Ctrl_1445	0.995275591	Control	Leeds
Case_694	1	Case	Leeds
Case_695	1	Case	Leeds
Ctrl_1446	1	Control	Leeds
Case_696	1	Case	Leeds
Case_697	1	Case	Leeds
Case_698	1	Case	Leeds
Ctrl_1447	1	Control	Leeds
Ctrl_1448	1	Control	Leeds
Ctrl_1449	1	Control	Leeds
Ctrl_1450	1	Control	Leeds
Ctrl_1451	1	Control	Leeds
Ctrl_1452	1	Control	Leeds
Ctrl_1453	1	Control	Leeds
Ctrl_1454	1	Control	Leeds
Case_699	1	Case	Leeds

Ctrl_1455	1	Control	Leeds
Ctrl_1456	1	Control	Leeds
Ctrl_1457	1	Control	Leeds
Case_700	1	Case	Leeds
Case_701	1	Case	Leeds
Case_702	1	Case	Leeds
Ctrl_1458	1	Control	Leeds
Ctrl_1459	1	Control	Leeds
Ctrl_1460	1	Control	Leeds
Ctrl_1461	1	Control	Leeds
Case_703	1	Case	Leeds
Case_704	1	Case	Leeds
Case_705	1	Case	Leeds
Case_706	1	Case	Leeds
Case_707	1	Case	Leeds
Case_708	1	Case	Leeds
Ctrl_1462	1	Control	Leeds
Ctrl_1463	1	Control	Leeds
Ctrl_1464	1	Control	Leeds
Ctrl_1465	1	Control	Leeds
Ctrl_1466	1	Control	Leeds
Ctrl_1467	1	Control	Leeds
Ctrl_1468	1	Control	Leeds
Ctrl_1469	1	Control	Leeds
Case_709	1	Case	Leeds
Ctrl_1470	1	Control	Leeds
Case_710	1	Case	Leeds
Ctrl_1471	1	Control	Leeds
Case_711	1	Case	Leeds
Case_712	1	Case	Leeds
Case_713	1	Case	Leeds
Case_714	1	Case	Leeds
Ctrl_1472	1	Control	Leeds
Case_715	1	Case	Leeds
PD30591a	1	Control	Leeds
Ctrl_1473	1	Control	Leeds
Ctrl_1474	1	Control	Leeds
Case_716	1	Case	Leeds
Case_717	1	Case	Leeds
Case_718	1	Case	Leeds

Case_719	1	Case	Leeds
Case_720	1	Case	Leeds
Case_721	1	Case	Leeds
Ctrl_1475	1	Control	Leeds
Ctrl_1476	1	Control	Leeds
Ctrl_1477	1	Control	Leeds
Case_722	1	Case	Leeds
Ctrl_1478	1	Control	Leeds
Ctrl_1479	1	Control	Leeds
Ctrl_1480	1	Control	Leeds
Ctrl_1481	1	Control	Leeds
Ctrl_1482	1	Control	Leeds
Case_723	1	Case	Leeds
Case_724	1	Case	Leeds
Ctrl_1483	1	Control	Leeds
Case_725	1	Case	Leeds
Ctrl_1484	1	Control	Leeds
Ctrl_1485	1	Control	Leeds
Ctrl_1486	1	Control	Leeds
Case_726	1	Case	Leeds
Case_727	1	Case	Leeds
Ctrl_1487	1	Control	Leeds
Ctrl_1488	1	Control	Leeds
Case_728	1	Case	Leeds
Case_729	1	Case	Leeds
Case_730	1	Case	Leeds
Case_731	1	Case	Leeds
Case_732	1	Case	Leeds
Ctrl_1489	1	Control	Leeds
Case_733	1	Case	Leeds
Case_734	1	Case	Leeds
Case_735	1	Case	Leeds
Ctrl_1490	1	Control	Leeds
Ctrl_1491	1	Control	Leeds
Case_736	1	Case	Leeds
Ctrl_1492	1	Control	Leeds
Case_737	1	Case	Leeds
Case_738	1	Case	Leeds
Ctrl_1493	1	Control	Leeds
Ctrl_1494	1	Control	Leeds

PD30592a	1	Control	Leeds
Ctrl_1495	1	Control	Leeds
Ctrl_1496	1	Control	Leeds
Ctrl_1497	1	Control	Leeds
Ctrl_1498	1	Control	Leeds
Ctrl_1499	1	Control	Leeds
Ctrl_1500	1	Control	Leeds
Ctrl_1501	1	Control	Leeds
Case_739	1	Case	Leeds
Case_740	1	Case	Leeds
Ctrl_1502	1	Control	Leeds
Ctrl_1503	1	Control	Leeds
Ctrl_1504	1	Control	Leeds
Ctrl_1505	1	Control	Leeds
Ctrl_1506	1	Control	Leeds
Ctrl_1507	1	Control	Leeds
Ctrl_1508	1	Control	Leeds
Ctrl_1509	1	Control	Leeds
Case_741	1	Case	Leeds
Ctrl_1510	1	Control	Leeds
Ctrl_1511	1	Control	Leeds
Ctrl_1512	1	Control	Leeds
Case_742	1	Case	Leeds
Case_743	1	Case	Leeds
Case_744	1	Case	Leeds
Case_745	1	Case	Leeds
Ctrl_1513	1	Control	Leeds
Case_746	1	Case	Leeds
Ctrl_1514	1	Control	Leeds
Case_747	1	Case	Leeds
Case_748	1	Case	Leeds
Ctrl_1515	1	Control	Leeds
Case_749	1	Case	Leeds
Ctrl_1516	1	Control	Leeds
Ctrl_1517	1	Control	Leeds
Ctrl_1518	1	Control	Leeds
Ctrl_1519	1	Control	Leeds
Ctrl_1520	1	Control	Leeds
Ctrl_1521	1	Control	Leeds
Ctrl_1522	1	Control	Leeds

Ctrl_1523	1	Control	Leeds
Ctrl_1524	1	Control	Leeds
Ctrl_1525	1	Control	Leeds
Ctrl_1526	1	Control	Leeds
Ctrl_1527	1	Control	Leeds
Ctrl_1528	1	Control	Leeds
Case_750	1	Case	Leeds
Ctrl_1529	1	Control	Leeds
Ctrl_1530	1	Control	Leeds
Case_751	1	Case	Leeds
Ctrl_1531	1	Control	Leeds
Case_752	1	Case	Leeds
Case_753	1	Case	Leeds
Case_754	1	Case	Leeds
Case_755	1	Case	Leeds
Case_756	1	Case	Leeds
Ctrl_1532	1	Control	Leeds
Case_757	1	Case	Leeds
Ctrl_1533	1	Control	Leeds
Ctrl_1534	1	Control	Leeds
Case_758	1	Case	Leeds
Case_759	1	Case	Leeds
Case_760	1	Case	Leeds
Ctrl_1535	1	Control	Leeds
Ctrl_1536	1	Control	Leeds
Case_761	1	Case	Leeds
Ctrl_1537	1	Control	Leeds
Case_762	1	Case	Leeds
Case_763	1	Case	Leeds
Case_764	1	Case	Leeds
Ctrl_1538	1	Control	Leeds
Ctrl_1539	1	Control	Leeds
Case_765	1	Case	Leeds
Case_766	1	Case	Leeds
Ctrl_1540	1	Control	Leeds
Case_767	1	Case	Leeds
Case_768	1	Case	Leeds
Ctrl_1541	1	Control	Leeds
Ctrl_1542	1	Control	Leeds
Ctrl_1543	1	Control	Leeds

Ctrl_1544	1	Control	Leeds
Ctrl_1545	1	Control	Leeds
Ctrl_1546	1	Control	Leeds
Ctrl_1547	1	Control	Leeds
Ctrl_1548	1	Control	Leeds
Ctrl_1549	1	Control	Leeds
Ctrl_1550	1	Control	Leeds
Case_769	1	Case	Leeds
PD30593a	1	Case	Leeds
Case_770	1	Case	Leeds
Case_771	1	Case	Leeds
Case_772	1	Case	Leeds
Case_773	1	Case	Leeds
Ctrl_1551	1	Control	Leeds
Ctrl_1552	1	Control	Leeds
Case_774	1	Case	Leeds
Case_775	1	Case	Leeds
Ctrl_1553	1	Control	Leeds
Case_776	1	Case	Leeds
Case_777	1	Case	Leeds
Ctrl_1554	1	Control	Leeds
Ctrl_1555	1	Control	Leeds
Ctrl_1556	1	Control	Leeds
Case_778	1	Case	Leeds
Case_779	1	Case	Leeds
Ctrl_1557	1	Control	Leeds
Case_780	1	Case	Leeds
Case_781	1	Case	Leeds
Case_782	1	Case	Leeds
Ctrl_1558	1	Control	Leeds
Case_783	1	Case	Leeds
Ctrl_1559	1	Control	Leeds
Ctrl_1560	1	Control	Leeds
Ctrl_1561	1	Control	Leeds
Ctrl_1562	1	Control	Leeds
Ctrl_1563	1	Control	Leeds
Ctrl_1564	1	Control	Leeds
Case_784	1	Case	Leeds
Case_785	1	Case	Leeds
Case_786	1	Case	Leeds

Case_787	1	Case	Leeds
Case_788	1	Case	Leeds
Ctrl_1565	1	Control	Leeds
Case_789	1	Case	Leeds
Ctrl_1566	1	Control	Leeds
Case_790	1	Case	Leeds
Ctrl_1567	1	Control	Leeds
Case_791	1	Case	Leeds
Ctrl_1568	1	Control	Leeds
Case_792	1	Case	Leeds
Case_793	1	Case	Leeds
Case_794	1	Case	Leeds
Ctrl_1569	1	Control	Leeds
Case_795	1	Case	Leeds
Case_796	1	Case	Leeds
Case_797	1	Case	Leeds
Case_798	1	Case	Leeds
Case_799	1	Case	Leeds
Case_800	1	Case	Leeds
PD30594a	1	Case	Leeds
Ctrl_1570	1	Control	Leeds
Case_801	1	Case	Leeds
Ctrl_1571	1	Control	Leeds
Ctrl_1572	1	Control	Leeds
Ctrl_1573	1	Control	Leeds
Case_802	1	Case	Leeds
Ctrl_1574	1	Control	Leeds
Case_803	1	Case	Leeds
Case_804	1	Case	Leeds
Ctrl_1575	1	Control	Leeds
Case_805	1	Case	Leeds
Ctrl_1576	1	Control	Leeds
Case_806	1	Case	Leeds
Ctrl_1577	1	Control	Leeds
Case_807	1	Case	Leeds
Case_808	1	Case	Leeds
Case_809	1	Case	Leeds
Case_810	1	Case	Leeds
Ctrl_1578	1	Control	Leeds
Ctrl_1579	1	Control	Leeds

Ctrl_1580	1	Control	Leeds
Ctrl_1581	1	Control	Leeds
Ctrl_1582	1	Control	Leeds
Ctrl_1583	1	Control	Leeds
Case_811	1	Case	Leeds
Case_812	1	Case	Leeds
Case_813	1	Case	Leeds
Case_814	1	Case	Leeds
Case_815	1	Case	Leeds
Ctrl_1584	1	Control	Leeds
Ctrl_1585	1	Control	Leeds
Ctrl_1586	1	Control	Leeds
Ctrl_1587	1	Control	Leeds
Case_816	1	Case	Leeds
Case_817	1	Case	Leeds
Case_818	1	Case	Leeds
Ctrl_1588	1	Control	Leeds
Ctrl_1589	1	Control	Leeds
Case_819	1	Case	Leeds
Case_820	1	Case	Leeds
Case_821	1	Case	Leeds
Ctrl_1590	1	Control	Leeds
Ctrl_1591	1	Control	Leeds
Ctrl_1592	1	Control	Leeds
Ctrl_1593	1	Control	Leeds
Ctrl_1594	1	Control	Leeds
Ctrl_1595	1	Control	Leeds
Ctrl_1596	1	Control	Leeds
Ctrl_1597	1	Control	Leeds
Ctrl_1598	1	Control	Leeds
Ctrl_1599	1	Control	Leeds
Ctrl_1600	1	Control	Leeds
Case_822	1	Case	Leeds
Case_823	1	Case	Leeds
Case_824	1	Case	Leeds
Case_825	1	Case	Leeds
Case_826	1	Case	Leeds
Case_827	1	Case	Leeds
Case_828	1	Case	Leeds
Case_829	1	Case	Leeds

Case_830	1	Case	Leeds
Case_831	1	Case	Leeds
Case_832	1	Case	Leeds
Case_833	1	Case	Leeds
Ctrl_1601	1	Control	Leeds
Case_834	1	Case	Leeds
Case_835	1	Case	Leeds
Case_836	1	Case	Leeds
Case_837	1	Case	Leeds
Case_838	1	Case	Leeds
Case_839	1	Case	Leeds
Case_840	1	Case	Leeds
Ctrl_1602	1	Control	Leeds
Case_841	1	Case	Leeds
Case_842	1	Case	Leeds
Case_843	1	Case	Leeds
Ctrl_1603	1	Control	Leeds
Case_844	1	Case	Leeds
Ctrl_1604	1	Control	Leeds
Ctrl_1605	1	Control	Leeds
Ctrl_1606	1	Control	Leeds
Case_845	1	Case	Leeds
PD30595a	1	Case	Leeds
Ctrl_1607	1	Control	Leeds
Ctrl_1608	1	Control	Leeds
Ctrl_1609	1	Control	Leeds
Case_846	1	Case	Leeds
Ctrl_1610	1	Control	Leeds
Case_847	1	Case	Leeds
Ctrl_1611	1	Control	Leeds
Ctrl_1612	1	Control	Leeds
Ctrl_1613	1	Control	Leeds
Case_848	1	Case	Leeds
Case_849	1	Case	Leeds
Ctrl_1614	1	Control	Leeds
Ctrl_1615	1	Control	Leeds
Ctrl_1616	1	Control	Leeds
Ctrl_1617	1	Control	Leeds
Ctrl_1618	1	Control	Leeds
Ctrl_1619	1	Control	Leeds

Case_850	1	Case	Leeds
Case_851	1	Case	Leeds
Case_852	1	Case	Leeds
Case_853	1	Case	Leeds
Case_854	1	Case	Leeds
Case_855	1	Case	Leeds
Case_856	1	Case	Leeds
Case_857	1	Case	Leeds
Case_858	1	Case	Leeds
Case_859	1	Case	Leeds
Ctrl_1620	1	Control	Leeds
Ctrl_1621	1	Control	Leeds
Ctrl_1622	1	Control	Leeds
Ctrl_1623	1	Control	Leeds
Ctrl_1624	1	Control	Leeds
Ctrl_1625	1	Control	Leeds
Ctrl_1626	1	Control	Leeds
Ctrl_1627	1	Control	Leeds
Ctrl_1628	1	Control	Leeds
Ctrl_1629	1	Control	Leeds
Ctrl_1630	1	Control	Leeds
Case_860	0.951706037	Case	Leeds
Case_861	1	Case	Leeds
Case_862	1	Case	Leeds
Case_863	1	Case	Leeds
Case_864	1	Case	Leeds
Ctrl_1631	1	Control	Leeds
Ctrl_1632	1	Control	Leeds
Ctrl_1633	1	Control	Leeds
Ctrl_1634	1	Control	Leeds
Ctrl_1635	1	Control	Leeds
Ctrl_1636	1	Control	Leeds
Ctrl_1637	1	Control	Leeds
Ctrl_1638	1	Control	Leeds
Case_865	1	Case	Leeds
Case_866	1	Case	Leeds
Case_867	1	Case	Leeds
Case_868	1	Case	Leeds
Ctrl_1639	1	Control	Leeds
Ctrl_1640	1	Control	Leeds

Ctrl_1641	1	Control	Leeds
Ctrl_1642	1	Control	Leeds
Ctrl_1643	1	Control	Leeds
Ctrl_1644	1	Control	Leeds
Ctrl_1645	1	Control	Leeds
Ctrl_1646	1	Control	Leeds
Case_869	1	Case	Leeds
Ctrl_1647	1	Control	Leeds
Ctrl_1648	1	Control	Leeds
Case_870	1	Case	Leeds
Case_871	1	Case	Leeds
Ctrl_1649	1	Control	Leeds
Ctrl_1650	1	Control	Leeds
Case_872	1	Case	Leeds
Case_873	1	Case	Leeds
Case_874	1	Case	Leeds
Case_875	1	Case	Leeds
Case_876	1	Case	Leeds
Ctrl_1651	1	Control	Leeds
Ctrl_1652	1	Control	Leeds
Ctrl_1653	1	Control	Leeds
Ctrl_1654	1	Control	Leeds
Ctrl_1655	1	Control	Leeds
Ctrl_1656	1	Control	Leeds
Ctrl_1657	1	Control	Leeds
Case_877	1	Case	Leeds
Ctrl_1658	1	Control	Leeds
Ctrl_1659	1	Control	Leeds
Ctrl_1660	1	Control	Leeds
Ctrl_1661	1	Control	Leeds
Case_878	1	Case	Leeds
Case_879	1	Case	Leeds
Ctrl_1662	1	Control	Leeds
Ctrl_1663	1	Control	Leeds
Ctrl_1664	1	Control	Leeds
Ctrl_1665	1	Control	Leeds
Case_880	1	Case	Leeds
Case_881	1	Case	Leeds
Case_882	1	Case	Leeds
Case_883	1	Case	Leeds

Case_884	1	Case	Leeds
Case_885	1	Case	Leeds
Case_886	1	Case	Leeds
Case_887	1	Case	Leeds
Case_888	1	Case	Leeds
Case_889	1	Case	Leeds
Case_890	1	Case	Leeds
Case_891	1	Case	Leeds
Case_892	1	Case	Leeds
Case_893	1	Case	Leeds
Case_894	1	Case	Leeds
Case_895	1	Case	Leeds
Case_896	1	Case	Leeds
Case_897	1	Case	Leeds
Case_898	1	Case	Leeds
Case_899	1	Case	Leeds
Case_900	1	Case	Leeds
Case_901	1	Case	Leeds
Case_902	1	Case	Leeds
Case_903	1	Case	Leeds
Case_904	1	Case	Leeds
Case_905	1	Case	Leeds
Case_906	1	Case	Leeds
Case_907	1	Case	Leeds
Case_908	1	Case	Leeds
Case_909	1	Case	Leeds
Case_910	1	Case	Leeds
Case_911	1	Case	Leeds
Ctrl_1666	1	Control	Leeds
Case_912	1	Case	Leeds
Case_913	1	Case	Leeds
Case_914	1	Case	Leeds
Ctrl_1667	1	Control	Leeds
PD30596a	1	Case	Leeds
Case_915	1	Case	Leeds
Case_916	1	Case	Leeds
Case_917	1	Case	Leeds
Case_918	1	Case	Leeds
Ctrl_1668	1	Control	Leeds
Ctrl_1669	1	Control	Leeds

Ctrl_1670	1	Control	Leeds
Case_919	1	Case	Leeds
Case_920	0.951706037	Case	Leeds
Case_921	1	Case	Leeds
Ctrl_1671	1	Control	Leeds
Ctrl_1672	1	Control	Leeds
Ctrl_1673	1	Control	Leeds
Ctrl_1674	1	Control	Leeds
Ctrl_1675	1	Control	Leeds
Ctrl_1676	1	Control	Leeds
Ctrl_1677	1	Control	Leeds
PD30611a	1	Control	Leeds
Ctrl_1678	1	Control	Leeds
Ctrl_1679	1	Control	Leeds
Ctrl_1680	1	Control	Leeds
PD30597a	1	Control	Leeds
Ctrl_1681	1	Control	Leeds
Ctrl_1682	1	Control	Leeds
Ctrl_1683	1	Control	Leeds
Ctrl_1684	1	Control	Leeds
Case_922	1	Case	Leeds
PD30598a	1	Case	Leeds
Case_923	1	Case	Leeds
Ctrl_1685	1	Control	Leeds
Ctrl_1686	1	Control	Leeds
Ctrl_1687	1	Control	Leeds
Ctrl_1688	1	Control	Leeds
Case_924	1	Case	Leeds
Case_925	1	Case	Leeds
Case_926	1	Case	Leeds
Case_927	1	Case	Leeds
Case_928	1	Case	Leeds
Case_929	1	Case	Leeds
Ctrl_1689	1	Control	Leeds
Case_930	1	Case	Leeds
Ctrl_1690	1	Control	Leeds
Case_931	1	Case	Leeds
Ctrl_1691	1	Control	Leeds
Ctrl_1692	1	Control	Leeds
Ctrl_1693	1	Control	Leeds

Case_932	1	Case	Leeds
Ctrl_1694	1	Control	Leeds
Ctrl_1695	1	Control	Leeds
Ctrl_1696	1	Control	Leeds
Ctrl_1697	1	Control	Leeds
PD30600a	1	Control	Leeds
Ctrl_1698	1	Control	Leeds
Ctrl_1699	1	Control	Leeds
Ctrl_1700	1	Control	Leeds
Ctrl_1701	1	Control	Leeds
Ctrl_1702	1	Control	Leeds
Ctrl_1703	1	Control	Leeds
Case_933	1	Case	Leeds
Case_934	1	Case	Leeds
PD30599a	1	Control	Leeds
Ctrl_1704	1	Control	Leeds
Case_935	1	Case	Leeds
Case_936	1	Case	Leeds
Case_937	1	Case	Leeds
Ctrl_1705	1	Control	Leeds
Ctrl_1706	1	Control	Leeds
Ctrl_1707	1	Control	Leeds
Ctrl_1708	1	Control	Leeds
Case_938	1	Case	Leeds
Ctrl_1709	1	Control	Leeds
Ctrl_1710	1	Control	Leeds
Ctrl_1711	1	Control	Leeds
Case_939	1	Case	Leeds
Case_940	1	Case	Leeds
Case_941	1	Case	Leeds
Case_942	1	Case	Leeds
Ctrl_1712	1	Control	Leeds
Case_943	1	Case	Leeds
Case_944	1	Case	Leeds
Case_945	1	Case	Leeds
Case_946	1	Case	Leeds
Case_947	1	Case	Leeds
Case_948	1	Case	Leeds
Case_949	1	Case	Leeds
Case_950	1	Case	Leeds

Ctrl_1713	1	Control	Leeds
Ctrl_1714	1	Control	Leeds
Ctrl_1715	1	Control	Leeds
Ctrl_1716	1	Control	Leeds
Ctrl_1717	1	Control	Leeds
Ctrl_1718	1	Control	Leeds
Ctrl_1719	1	Control	Leeds
Ctrl_1720	1	Control	Leeds
Ctrl_1721	1	Control	Leeds
Ctrl_1722	1	Control	Leeds
Ctrl_1723	1	Control	Leeds
Ctrl_1724	1	Control	Leeds
Ctrl_1725	1	Control	Leeds
Ctrl_1726	1	Control	Leeds
Ctrl_1727	1	Control	Leeds
Ctrl_1728	1	Control	Leeds
Ctrl_1729	1	Control	Leeds
Ctrl_1730	1	Control	Leeds
Ctrl_1731	1	Control	Leeds
Ctrl_1732	1	Control	Leeds
Case_951	1	Case	Leeds
Ctrl_1733	1	Control	Leeds
Ctrl_1734	1	Control	Leeds
Case_952	1	Case	Leeds
Case_953	1	Case	Leeds
PD30601a	1	Case	Leeds
Case_954	1	Case	Leeds
Ctrl_1735	1	Control	Leeds
Ctrl_1736	1	Control	Leeds
Ctrl_1737	1	Control	Leeds
Ctrl_1738	1	Control	Leeds
Ctrl_1739	1	Control	Leeds
Ctrl_1740	1	Control	Leeds
Case_955	1	Case	Leeds
Ctrl_1741	1	Control	Leeds
Ctrl_1742	1	Control	Leeds
Ctrl_1743	1	Control	Leeds
Ctrl_1744	1	Control	Leeds
Case_956	1	Case	Leeds
Case_957	1	Case	Leeds

Case_958	1	Case	Leeds
Case_959	1	Case	Leeds
Case_960	1	Case	Leeds
Case_961	1	Case	Leeds
Ctrl_1745	1	Control	Leeds
Ctrl_1746	1	Control	Leeds
Ctrl_1747	1	Control	Leeds
Ctrl_1748	1	Control	Leeds
Case_962	1	Case	Leeds
Case_963	1	Case	Leeds
PD30602a	1	Case	Leeds
Case_964	1	Case	Leeds
Case_965	1	Case	Leeds
Case_966	1	Case	Leeds
Case_967	1	Case	Leeds
Ctrl_1749	1	Control	Leeds
Ctrl_1750	1	Control	Leeds
Ctrl_1751	1	Control	Leeds
Ctrl_1752	1	Control	Leeds
Case_968	1	Case	Leeds
Case_969	1	Case	Leeds
Case_970	1	Case	Leeds
Ctrl_1753	1	Control	Leeds
Case_971	1	Case	Leeds
Case_972	1	Case	Leeds
Case_973	1	Case	Leeds
Case_974	1	Case	Leeds
Case_975	1	Case	Leeds
Case_976	1	Case	Leeds
Case_977	1	Case	Leeds
Case_978	1	Case	Leeds
Case_979	1	Case	Leeds
Case_980	1	Case	Leeds
Case_981	1	Case	Leeds
Ctrl_1754	1	Control	Leeds
Case_982	1	Case	Leeds
Case_983	1	Case	Leeds
Case_984	1	Case	Leeds
Case_985	1	Case	Leeds
Case_986	1	Case	Leeds

Case_987	1	Case	Leeds
Case_988	1	Case	Leeds
Case_989	1	Case	Leeds
Case_990	1	Case	Leeds
Case_991	1	Case	Leeds
Case_992	1	Case	Leeds
Case_993	1	Case	Leeds
Case_994	1	Case	Leeds
Case_995	1	Case	Leeds
Case_996	1	Case	Leeds
Case_997	1	Case	Leeds
Case_998	1	Case	Leeds
Case_999	1	Case	Leeds
Case_1000	1	Case	Leeds
Case_1001	1	Case	Leeds
Case_1002	1	Case	Leeds
Case_1003	1	Case	Leeds
Case_1004	1	Case	Leeds
Case_1005	1	Case	Leeds
Case_1006	1	Case	Leeds
Case_1007	1	Case	Leeds
Case_1008	1	Case	Leeds
Case_1009	1	Case	Leeds
Case_1010	1	Case	Leeds
Case_1011	1	Case	Leeds
Case_1012	1	Case	Leeds
Case_1013	1	Case	Leeds
Case_1014	1	Case	Leeds
Case_1015	1	Case	Leeds
Case_1016	1	Case	Leeds
Case_1017	1	Case	Leeds
Case_1018	1	Case	Leeds
Case_1019	1	Case	Leeds
Case_1020	1	Case	Leeds
Case_1021	1	Case	Leeds
Case_1022	1	Case	Leeds
Case_1023	1	Case	Leeds
Ctrl_1755	1	Control	Leeds
Case_1024	1	Case	Leeds
Ctrl_1756	1	Control	Leeds

Case_1025	1	Case	Leeds
Case_1026	1	Case	Leeds
Case_1027	1	Case	Leeds
Ctrl_1757	1	Control	Leeds
Ctrl_1758	1	Control	Leeds
Ctrl_1759	1	Control	Leeds
Case_1028	1	Case	Leeds
Case_1029	1	Case	Leeds
Case_1030	1	Case	Leeds
Case_1031	1	Case	Leeds
Case_1032	1	Case	Leeds
Ctrl_1760	1	Control	Leeds
Ctrl_1761	1	Control	Leeds
Ctrl_1762	1	Control	Leeds
Ctrl_1763	1	Control	Leeds
Ctrl_1764	1	Control	Leeds
Ctrl_1765	1	Control	Leeds
Ctrl_1766	1	Control	Leeds
Ctrl_1767	1	Control	Leeds
Case_1033	1	Case	Leeds
Case_1034	1	Case	Leeds
Case_1035	1	Case	Leeds
Case_1036	1	Case	Leeds
PD30603a	1	Case	Leeds
Case_1037	1	Case	Leeds
Case_1038	1	Case	Leeds
Case_1039	1	Case	Leeds
Case_1040	1	Case	Leeds
Case_1041	1	Case	Leeds
PD30612a	1	Case	Leeds
Case_1042	1	Case	Leeds
Case_1043	1	Case	Leeds
Case_1044	1	Case	Leeds
Case_1045	1	Case	Leeds
Case_1046	1	Case	Leeds
Case_1047	1	Case	Leeds
Case_1048	1	Case	Leeds
Case_1049	1	Case	Leeds
Case_1050	1	Case	Leeds
Case_1051	1	Case	Leeds

Case_1052	1	Case	Leeds
Case_1053	1	Case	Leeds
Case_1054	1	Case	Leeds
Case_1055	1	Case	Leeds
Case_1056	1	Case	Leeds
Case_1057	1	Case	Leeds
Case_1058	1	Case	Leeds
Case_1059	1	Case	Leeds
Case_1060	1	Case	Leeds
Case_1061	1	Case	Leeds
Case_1062	1	Case	Leeds
Case_1063	1	Case	Leeds
Case_1064	1	Case	Leeds
Case_1065	1	Case	Leeds
Case_1066	1	Case	Leeds
Case_1067	1	Case	Leeds
Case_1068	1	Case	Leeds
Case_1069	1	Case	Leeds
Case_1070	1	Case	Leeds
Ctrl_1768	1	Control	Leeds
Ctrl_1769	1	Control	Leeds
Ctrl_1770	1	Control	Leeds
Ctrl_1771	1	Control	Leeds
Ctrl_1772	1	Control	Leeds
Ctrl_1773	1	Control	Leeds
Case_1071	1	Case	Leeds
Ctrl_1774	1	Control	Leeds
Ctrl_1775	1	Control	Leeds
Ctrl_1776	1	Control	Leeds
Ctrl_1777	1	Control	Leeds
Case_1072	1	Case	Leeds
PD30606a	1	Control	Leeds
Case_1073	1	Case	Leeds
Ctrl_1778	1	Control	Leeds
Case_1074	1	Case	Leeds
Case_1075	1	Case	Leeds
Ctrl_1779	1	Control	Leeds
Ctrl_1780	1	Control	Leeds
Ctrl_1781	1	Control	Leeds
Case_1076	1	Case	Leeds

Ctrl_1782	1	Control	Leeds
Ctrl_1783	1	Control	Leeds
Case_1077	1	Case	Leeds
Ctrl_1784	1	Control	Leeds
Ctrl_1785	1	Control	Leeds
Ctrl_1786	1	Control	Leeds
Ctrl_1787	1	Control	Leeds
Ctrl_1788	1	Control	Leeds
Case_1078	1	Case	Leeds
Case_1079	1	Case	Leeds
Case_1080	1	Case	Leeds
Case_1081	1	Case	Leeds
Case_1082	1	Case	Leeds
Ctrl_1789	1	Control	Leeds
Ctrl_1790	1	Control	Leeds
Case_1083	1	Case	Leeds
Case_1084	1	Case	Leeds
Ctrl_1791	1	Control	Leeds
Case_1085	1	Case	Leeds
Case_1086	1	Case	Leeds
Ctrl_1792	1	Control	Leeds
Case_1087	1	Case	Leeds
Case_1088	1	Case	Leeds
Case_1089	1	Case	Leeds
PD30607a	1	Case	Leeds
Case_1090	1	Case	Leeds
Ctrl_1793	1	Control	Leeds
Case_1091	1	Case	Leeds
Case_1092	1	Case	Leeds
Case_1093	1	Case	Leeds
Case_1094	1	Case	Leeds
Case_1095	1	Case	Leeds
Case_1096	1	Case	Leeds
PD30604a	1	Case	Leeds
Ctrl_1794	1	Control	Leeds
Ctrl_1795	1	Control	Leeds
Ctrl_1796	1	Control	Leeds
Ctrl_1797	1	Control	Leeds
Ctrl_1798	1	Control	Leeds
Case_1097	1	Case	Leeds

Case_1098	1	Case	Leeds
Case_1099	1	Case	Leeds
Case_1100	1	Case	Leeds
Case_1101	1	Case	Leeds
Case_1102	1	Case	Leeds
Case_1103	1	Case	Leeds
Case_1104	1	Case	Leeds
Case_1105	1	Case	Leeds
Ctrl_1799	1	Control	Leeds
Ctrl_1800	1	Control	Leeds
Ctrl_1801	1	Control	Leeds
Ctrl_1802	1	Control	Leeds
Ctrl_1803	1	Control	Leeds
Ctrl_1804	1	Control	Leeds
Case_1106	1	Case	Leeds
Case_1107	1	Case	Leeds
Ctrl_1805	1	Control	Leeds
Ctrl_1806	1	Control	Leeds
Ctrl_1807	1	Control	Leeds
PD30605a	1	Case	Leeds
Ctrl_1808	1	Control	Leeds
Ctrl_1809	1	Control	Leeds
Ctrl_1810	1	Control	Leeds
Ctrl_1811	1	Control	Leeds
Ctrl_1812	1	Control	Leeds
Ctrl_1813	1	Control	Leeds
Ctrl_1814	1	Control	Leeds
Ctrl_1815	1	Control	Leeds
Ctrl_1816	1	Control	Leeds
Ctrl_1817	1	Control	Leeds
Ctrl_1818	1	Control	Leeds
Ctrl_1819	1	Control	Leeds
Ctrl_1820	1	Control	Leeds
Ctrl_1821	1	Control	Leeds
Ctrl_1822	1	Control	Leeds
Ctrl_1823	1	Control	Leeds
Ctrl_1824	1	Control	Leeds
Ctrl_1825	1	Control	Leeds
Ctrl_1826	1	Control	Leeds
Ctrl_1827	1	Control	Leeds

Ctrl_1828	1	Control	Leeds
Ctrl_1829	1	Control	Leeds
Ctrl_1830	1	Control	Leeds
Ctrl_1831	1	Control	Leeds
PD30608a	1	Control	Leeds
Case_1108	1	Case	Leeds
Case_1109	1	Case	Leeds
Case_1110	1	Case	Leeds
Case_1111	1	Case	Leeds
Case_1112	1	Case	Leeds
Case_1113	1	Case	Leeds
Ctrl_1832	1	Control	Leeds
Ctrl_1833	1	Control	Leeds
Ctrl_1834	1	Control	Leeds
Ctrl_1835	1	Control	Leeds
Ctrl_1836	1	Control	Leeds
Case_1114	1	Case	Leeds
Case_1115	1	Case	Leeds
Case_1116	1	Case	Leeds
Case_1117	1	Case	Leeds
Ctrl_1837	1	Control	Leeds
Ctrl_1838	1	Control	Leeds
Case_1118	1	Case	Leeds
Ctrl_1839	1	Control	Leeds
Ctrl_1840	1	Control	Leeds
Ctrl_1841	1	Control	Leeds
Ctrl_1842	1	Control	Leeds
Ctrl_1843	1	Control	Leeds
Ctrl_1844	0.999475066	Control	Leeds
Ctrl_1845	1	Control	Leeds
Case_1119	1	Case	Leeds
Case_1120	1	Case	Leeds
Case_1121	1	Case	Leeds
Case_1122	1	Case	Leeds
Case_1123	1	Case	Leeds
Ctrl_1846	1	Control	Leeds
Ctrl_1847	1	Control	Leeds
Ctrl_1848	1	Control	Leeds
Ctrl_1849	1	Control	Leeds
Ctrl_1850	1	Control	Leeds

Ctrl_1851	1	Control	Leeds
Ctrl_1852	1	Control	Leeds
Ctrl_1853	1	Control	Leeds
Ctrl_1854	1	Control	Leeds
Ctrl_1855	1	Control	Leeds
Ctrl_1856	1	Control	Leeds
Case_1124	1	Case	Leeds
Case_1125	1	Case	Leeds
Case_1126	1	Case	Leeds
Case_1127	1	Case	Leeds
Case_1128	1	Case	Leeds
Case_1129	1	Case	Leeds
Case_1130	1	Case	Leeds
Case_1131	1	Case	Leeds
Case_1132	1	Case	Leeds
Case_1133	1	Case	Leeds
Case_1134	1	Case	Leeds
Case_1135	1	Case	Leeds
Case_1136	1	Case	Leeds
Case_1137	1	Case	Leeds
Case_1138	1	Case	Leeds
Case_1139	1	Case	Leeds
Case_1140	1	Case	Leeds
Case_1141	1	Case	Leeds
Case_1142	1	Case	Leeds
Case_1143	1	Case	Leeds
PD30609a	1	Case	Leeds
Case_1144	1	Case	Leeds
Case_1145	1	Case	Leeds
Case_1146	1	Case	Leeds
Case_1147	1	Case	Leeds
Case_1148	1	Case	Leeds
Case_1149	1	Case	Leeds
Case_1150	1	Case	Leeds
Case_1151	1	Case	Leeds
Case_1152	1	Case	Leeds
Case_1153	1	Case	Leeds
Case_1154	1	Case	Leeds
Case_1155	1	Case	Leeds
Case_1156	1	Case	Leeds

Case_1157	1	Case	Leeds
PD30614a	0.985826772	Case	Leeds
Case_1158	1	Case	Leeds
Case_1159	1	Case	Leeds
PD30615a	1	Case	Leeds
PD30610a	1	Case	Leeds
Case_1160	1	Case	Leeds
Case_1161	1	Case	Leeds
Case_1162	1	Case	Leeds
Case_1163	1	Case	Leeds
Case_1164	1	Case	Leeds
Case_1165	1	Case	Leeds
Case_1166	1	Case	Leeds
Case_1167	1	Case	Leeds
Case_1168	1	Case	Leeds
Case_1169	1	Case	Leeds
Case_1170	1	Case	Leeds
Case_1171	1	Case	Leeds
PD30616a	1	Case	Leeds
Case_1172	1	Case	Leeds
Case_1173	1	Case	Leeds
Case_1174	1	Case	Leeds
Case_1175	1	Case	Leeds
Case_1176	1	Case	Leeds
Case_1177	1	Case	Leeds
Case_1178	0.951706037	Case	Leeds
Case_1179	1	Case	Leeds
Case_1180	1	Case	Leeds
Case_1181	1	Case	Leeds
Case_1182	1	Case	Leeds
Case_1183	1	Case	Leeds
PD30618a	1	Case	Leeds
Case_1184	1	Case	Leeds
PD30619a	1	Case	Leeds
Case_1185	1	Case	Leeds
Case_1186	1	Case	Leeds
Case_1187	1	Case	Leeds
PD30621a	1	Case	Leeds
Case_1188	1	Case	Leeds
Case_1189	1	Case	Leeds

Case_1190	0.998950131	Case	Leeds
Case_1191	1	Case	Leeds
Case_1192	1	Case	Leeds
Case_1193	1	Case	Leeds
Case_1194	1	Case	Leeds
Case_1195	1	Case	Leeds
Case_1196	0.951706037	Case	Leeds
Case_1197	1	Case	Leeds
Case_1198	1	Case	Leeds
PD30622a	1	Case	Leeds
PD30623a	1	Case	Leeds
Case_1199	1	Case	Leeds
PD30624a	1	Case	Leeds
Case_1200	1	Case	Leeds
PD30625a	1	Case	Leeds
Case_1201	1	Case	Leeds
Case_1202	1	Case	Leeds
Case_1203	1	Case	Leeds
Case_1204	1	Case	Leeds
Case_1205	1	Case	Leeds
PD30626a	1	Case	Leeds
Case_1206	1	Case	Leeds
PD30627a	1	Case	Leeds
Case_1207	1	Case	Leeds
Case_1208	1	Case	Leeds
PD30628a	1	Case	Leeds
PD30629a	1	Case	Leeds
PD30630a	1	Case	Leeds
Case_1209	1	Case	Leeds
Case_1210	1	Case	Leeds
PD30631a	1	Case	Leeds
PD30632a	1	Case	Leeds
PD30633a	1	Case	Leeds
PD30634a	1	Case	Leeds
PD30635a	1	Case	Leeds
Case_1211	1	Case	Leeds
PD30636a	1	Case	Leeds
PD30637a	0.985301837	Case	Leeds
Case_1212	1	Case	Leeds
Case_1213	1	Case	Leeds

Case_1214	1	Case	Leeds
PD30642a	1	Case	Leeds
PD30643a	1	Case	Leeds
PD30644a	1	Case	Leeds
Case_1215	1	Case	Leeds
PD30638a	1	Case	Leeds
PD30646a	1	Case	Leeds
Case_1216	1	Case	Leeds
Case_1217	0.995275591	Case	Leeds
Case_1218	0.940682415	Case	Leeds
PD30647a	0.940682415	Case	Leeds
Case_1219	1	Case	Leeds
Case_1220	0.95328084	Case	Leeds
PD30648a	1	Case	Leeds
Case_1221	1	Case	Leeds
PD30650a	1	Case	Leeds
PD30651a	1	Case	Leeds
PD30652a	0.999475066	Case	Leeds
Case_1222	1	Case	Leeds
PD30653a	1	Case	Leeds
Case_1223	1	Case	Leeds
Case_1224	1	Case	Leeds
PD30654a	1	Case	Leeds
PD30655a	1	Case	Leeds
PD30656a	0.949606299	Case	Leeds
Case_1225	1	Case	Leeds
Case_1226	1	Case	Leeds
PD30657a	1	Case	Leeds
PD30658a	1	Case	Leeds
PD30659a	1	Case	Leeds
PD30639a	1	Case	Leeds
Case_1227	1	Case	Leeds
PD30660a	1	Case	Leeds
Case_1228	1	Case	Leeds
Case_1229	1	Case	Leeds
PD30697a	1	Case	Leeds
PD30661a	1	Case	Leeds
Case_1230	1	Case	Leeds
Case_1231	1	Case	Leeds
Case_1232	1	Case	Leeds

Case_1233	1	Case	Leeds
Case_1234	1	Case	Leeds
Case_1235	1	Case	Leeds
Case_1236	1	Case	Leeds
PD30662a	1	Case	Leeds
Case_1237	1	Case	Leeds
Case_1238	1	Case	Leeds
PD30663a	0.995275591	Case	Leeds
PD30664a	1	Case	Leeds
Case_1239	1	Case	Leeds
Case_1240	0.984251969	Case	Leeds
PD30668a	1	Case	Leeds
Case_1241	1	Case	Leeds
Case_1242	1	Case	Leeds
Case_1243	1	Case	Leeds
Case_1244	1	Case	Leeds
Case_1245	1	Case	Leeds
Case_1246	1	Case	Leeds
Case_1247	1	Case	Leeds
Case_1248	1	Case	Leeds
PD30640a	1	Case	Leeds
Case_1249	1	Case	Leeds
Case_1250	1	Case	Leeds
PD30681a	1	Case	Leeds
PD30682a	1	Case	Leeds
PD30683a	1	Case	Leeds
Case_1251	1	Case	Leeds
PD30684a	1	Case	Leeds
Case_1252	1	Case	Leeds
Case_1253	1	Case	Leeds
Case_1254	1	Case	Leeds
Case_1255	1	Case	Leeds
Case_1256	1	Case	Leeds
Case_1257	1	Case	Leeds
Case_1258	1	Case	Leeds
Case_1259	1	Case	Leeds
Case_1260	1	Case	Leeds
PD30665a	1	Case	Leeds
Case_1261	1	Case	Leeds
PD30687a	1	Case	Leeds

Case_1262	1	Case	Leeds
Case_1263	1	Case	Leeds
Case_1264	1	Case	Leeds
Case_1265	1	Case	Leeds
PD30689a	1	Case	Leeds
Case_1266	1	Case	Leeds
Case_1267	1	Case	Leeds
Case_1268	1	Case	Leeds
Case_1269	1	Case	Leeds
Case_1270	1	Case	Leeds
Case_1271	1	Case	Leeds
Case_1272	1	Case	Leeds
Case_1273	1	Case	Leeds
Case_1274	1	Case	Leeds
Case_1275	1	Case	Leeds
Case_1276	1	Case	Leeds
Case_1277	1	Case	Leeds
Case_1278	1	Case	Leeds
Case_1279	1	Case	Leeds
Case_1280	1	Case	Leeds
Case_1281	1	Case	Leeds
Case_1282	1	Case	Leeds
Case_1283	1	Case	Leeds
Case_1284	1	Case	Leeds
Case_1285	1	Case	Leeds
Case_1286	1	Case	Leeds
PD30692a	1	Case	Leeds
Case_1287	1	Case	Leeds
Case_1288	1	Case	Leeds
Case_1289	1	Case	Leeds
Case_1290	1	Case	Leeds
PD30693a	1	Case	Leeds
Case_1291	1	Case	Leeds
Case_1292	1	Case	Leeds
Case_1293	1	Case	Leeds
Case_1294	1	Case	Leeds
Case_1295	1	Case	Leeds
Case_1296	1	Case	Leeds
Case_1297	1	Case	Leeds
Case_1298	1	Case	Leeds

Case_1299	1	Case	Leeds
Case_1300	1	Case	Leeds
Case_1301	1	Case	Leeds
Case_1302	1	Case	Leeds
PD30694a	1	Case	Leeds
Case_1303	1	Case	Leeds
Case_1304	1	Case	Leeds
Case_1305	1	Case	Leeds
Case_1306	1	Case	Leeds
Case_1307	1	Case	Leeds
Case_1308	1	Case	Leeds
Case_1309	1	Case	Leeds
Case_1310	1	Case	Leeds
Case_1311	1	Case	Leeds
Case_1312	1	Case	Leeds
Case_1313	1	Case	Leeds
PD30711a	1	Case	Leeds
Case_1314	1	Case	Leeds
Case_1315	1	Case	Leeds
Case_1316	1	Case	Leeds
Case_1317	1	Case	Leeds
Case_1318	1	Case	Leeds
Case_1319	1	Case	Leeds
Case_1320	1	Case	Leeds
Case_1321	1	Case	Leeds
Case_1322	1	Case	Leeds
Case_1323	1	Case	Leeds
Case_1324	1	Case	Leeds
Case_1325	1	Case	Leeds
Case_1326	1	Case	Leeds
Case_1327	1	Case	Leeds
Case_1328	1	Case	Leeds
Case_1329	1	Case	Leeds
Case_1330	1	Case	Leeds
Case_1331	1	Case	Leeds
PD30696a	1	Case	Leeds
Case_1332	1	Case	Leeds
Case_1333	1	Case	Leeds
Case_1334	1	Case	Leeds
Case_1335	1	Case	Leeds

Case_1336	1	Case	Leeds
Case_1337	1	Case	Leeds
Case_1338	1	Case	Leeds
Case_1339	1	Case	Leeds
Case_1340	1	Case	Leeds
Case_1341	1	Case	Leeds
Case_1342	1	Case	Leeds
Case_1343	1	Case	Leeds
Case_1344	1	Case	Leeds
Case_1345	1	Case	Leeds
Case_1346	1	Case	Leeds
Case_1347	1	Case	Leeds
Case_1348	1	Case	Leeds
Case_1349	1	Case	Leeds
Case_1350	1	Case	Leeds
Case_1351	1	Case	Leeds
Case_1352	1	Case	Leeds
Case_1353	1	Case	Leeds
Case_1354	1	Case	Leeds
Case_1355	1	Case	Leeds
Case_1356	1	Case	Leeds
Case_1357	1	Case	Leeds
Case_1358	1	Case	Leeds
Case_1359	1	Case	Leeds
Case_1360	1	Case	Leeds
Case_1361	1	Case	Leeds
Case_1362	1	Case	Leeds
Case_1363	1	Case	Leeds
Case_1364	1	Case	Leeds
Case_1365	1	Case	Leeds
Case_1366	1	Case	Leeds
Case_1367	1	Case	Leeds
Case_1368	1	Case	Leeds
Case_1369	1	Case	Leeds
Case_1370	1	Case	Leeds
Case_1371	1	Case	Leeds
Case_1372	1	Case	Leeds
Case_1373	1	Case	Leeds
Case_1374	1	Case	Leeds
Case_1375	1	Case	Leeds

Case_1376	1	Case	Leeds
Case_1377	1	Case	Leeds
Case_1378	1	Case	Leeds
Case_1379	1	Case	Leeds
Case_1380	1	Case	Leeds
Case_1381	1	Case	Leeds
Case_1382	1	Case	Leeds
Case_1383	1	Case	Leeds
Case_1384	1	Case	Leeds
Case_1385	1	Case	Leeds
Case_1386	1	Case	Leeds
Case_1387	1	Case	Leeds
Case_1388	1	Case	Leeds
Case_1389	1	Case	Leeds
Case_1390	1	Case	Leeds
Case_1391	1	Case	Leeds
Case_1392	1	Case	Leeds
Case_1393	1	Case	Leeds
Case_1394	1	Case	Leeds
Case_1395	1	Case	Leeds
Case_1396	1	Case	Leeds
Case_1397	1	Case	Leeds
Case_1398	1	Case	Leeds
Case_1399	1	Case	Leeds
Case_1400	1	Case	Leeds
Case_1401	1	Case	Leeds
Case_1402	1	Case	Leeds
Case_1403	1	Case	Leeds
Case_1404	1	Case	Leeds
Case_1405	1	Case	Leeds
Case_1406	1	Case	Leeds
Case_1407	1	Case	Leeds
Case_1408	1	Case	Leeds
Case_1409	1	Case	Leeds
Case_1410	1	Case	Leeds
Case_1411	1	Case	Leeds
Case_1412	1	Case	Leeds
Case_1413	1	Case	Leeds
Case_1414	1	Case	Leeds
Case_1415	1	Case	Leeds

Case_1416	1	Case	Leeds
Case_1417	1	Case	Leeds
Case_1418	1	Case	Leeds
Case_1419	1	Case	Leeds
Case_1420	1	Case	Leeds
Case_1421	1	Case	Leeds
Case_1422	1	Case	Leeds
Case_1423	1	Case	Leeds
Case_1424	1	Case	Leeds
Case_1425	1	Case	Leeds
Case_1426	1	Case	Leeds
Case_1427	1	Case	Leeds
Case_1428	1	Case	Leeds
Case_1429	1	Case	Leeds
Case_1430	1	Case	Leeds
Case_1431	1	Case	Leeds
Case_1432	1	Case	Leeds
Case_1433	1	Case	Leeds
Case_1434	1	Case	Leeds
Case_1435	1	Case	Leeds
Case_1436	1	Case	Leeds
Case_1437	1	Case	Leeds
Case_1438	1	Case	Leeds
Case_1439	1	Case	Leeds
Case_1440	1	Case	Leeds
Case_1441	1	Case	Leeds
Case_1442	1	Case	Leeds
Case_1443	1	Case	Leeds
Case_1444	1	Case	Leeds
Case_1445	1	Case	Leeds
Case_1446	1	Case	Leeds
Case_1447	1	Case	Leeds
Case_1448	1	Case	Leeds
Case_1449	1	Case	Leeds
Case_1450	1	Case	Leeds
Case_1451	1	Case	Leeds
Case_1452	1	Case	Leeds
Case_1453	1	Case	Leeds
Case_1454	1	Case	Leeds
Case_1455	1	Case	Leeds

Case_1456	1	Case	Leeds
Case_1457	1	Case	Leeds
Case_1458	1	Case	Leeds
Case_1459	1	Case	Leeds
Case_1460	1	Case	Leeds
Case_1461	1	Case	Leeds
Case_1462	1	Case	Leeds
Case_1463	1	Case	Leeds
Case_1464	1	Case	Leeds
PD30698a	1	Case	Leeds
Case_1465	1	Case	Leeds
Case_1466	1	Case	Leeds
Case_1467	1	Case	Leeds
Case_1468	1	Case	Leeds
Case_1469	1	Case	Leeds
Case_1470	1	Case	Leeds
Case_1471	1	Case	Leeds
Case_1472	1	Case	Leeds
Case_1473	1	Case	Leeds
Case_1474	1	Case	Leeds
Case_1475	1	Case	Leeds
Case_1476	1	Case	Leeds
Case_1477	1	Case	Leeds
Case_1478	1	Case	Leeds
Case_1479	1	Case	Leeds
Case_1480	1	Case	Leeds
Case_1481	1	Case	Leeds
Case_1482	1	Case	Leeds
Case_1483	1	Case	Leeds
Case_1484	1	Case	Leeds
Case_1485	1	Case	Leeds
Ctrl_1857	1	Control	SEARCH
Ctrl_1858	1	Control	SEARCH
Ctrl_1859	0.996850394	Control	SEARCH
Ctrl_1860	1	Control	SEARCH
Ctrl_1861	0.94855643	Control	SEARCH
Ctrl_1862	1	Control	SEARCH
Ctrl_1863	1	Control	SEARCH
Ctrl_1864	1	Control	SEARCH
Ctrl_1865	0.94855643	Control	SEARCH

Ctrl_1866	1	Control	SEARCH
Ctrl_1867	1	Control	SEARCH
Ctrl_1868	1	Control	SEARCH
Ctrl_1869	0.94855643	Control	SEARCH
Ctrl_1870	0.999475066	Control	SEARCH
Ctrl_1871	0.94855643	Control	SEARCH
Ctrl_1872	1	Control	SEARCH
PD30545a	1	Control	SEARCH
Ctrl_1873	0.94855643	Control	SEARCH
Ctrl_1874	1	Control	SEARCH
Ctrl_1875	0.94855643	Control	SEARCH
Ctrl_1876	1	Control	SEARCH
Ctrl_1877	1	Control	SEARCH
Ctrl_1878	1	Control	SEARCH
Ctrl_1879	1	Control	SEARCH
Ctrl_1880	0.999475066	Control	SEARCH
Ctrl_1881	1	Control	SEARCH
Ctrl_1882	1	Control	SEARCH
Ctrl_1883	1	Control	SEARCH
Ctrl_1884	1	Control	SEARCH
Ctrl_1885	0.94855643	Control	SEARCH
Ctrl_1886	1	Control	SEARCH
Ctrl_1887	0.94855643	Control	SEARCH
Ctrl_1888	1	Control	SEARCH
Ctrl_1889	1	Control	SEARCH
Ctrl_1890	1	Control	SEARCH
Ctrl_1891	1	Control	SEARCH
Ctrl_1892	0.94855643	Control	SEARCH
PD30558a	1	Control	SEARCH
Ctrl_1893	0.94855643	Control	SEARCH
Ctrl_1894	0.94855643	Control	SEARCH
Ctrl_1895	1	Control	SEARCH
Ctrl_1896	0.94855643	Control	SEARCH
Ctrl_1897	1	Control	SEARCH
Ctrl_1898	1	Control	SEARCH
Ctrl_1899	1	Control	SEARCH
Ctrl_1900	1	Control	SEARCH
Ctrl_1901	1	Control	SEARCH
Ctrl_1902	0.94855643	Control	SEARCH
PD30559a	1	Control	SEARCH

Ctrl_1903	0.94855643	Control	SEARCH
Ctrl_1904	0.94855643	Control	SEARCH
Ctrl_1905	1	Control	SEARCH
Ctrl_1906	1	Control	SEARCH
Ctrl_1907	0.94855643	Control	SEARCH
Ctrl_1908	1	Control	SEARCH
Ctrl_1909	1	Control	SEARCH
Ctrl_1910	0.94855643	Control	SEARCH
Ctrl_1911	1	Control	SEARCH
Ctrl_1912	1	Control	SEARCH
Ctrl_1913	0.94855643	Control	SEARCH
Ctrl_1914	0.94855643	Control	SEARCH
Ctrl_1915	1	Control	SEARCH
Ctrl_1916	1	Control	SEARCH
Ctrl_1917	1	Control	SEARCH
Ctrl_1918	1	Control	SEARCH
Ctrl_1919	0.94855643	Control	SEARCH
Ctrl_1920	1	Control	SEARCH
Ctrl_1921	0.94855643	Control	SEARCH
Ctrl_1922	1	Control	SEARCH
Ctrl_1923	1	Control	SEARCH
Ctrl_1924	0.94855643	Control	SEARCH
Ctrl_1925	1	Control	SEARCH
Ctrl_1926	1	Control	SEARCH
Ctrl_1927	1	Control	SEARCH
Ctrl_1928	0.94855643	Control	SEARCH
Ctrl_1929	1	Control	SEARCH
Ctrl_1930	1	Control	SEARCH
Ctrl_1931	1	Control	SEARCH
Ctrl_1932	1	Control	SEARCH
Ctrl_1933	0.94855643	Control	SEARCH
Ctrl_1934	0.94855643	Control	SEARCH
Ctrl_1935	1	Control	SEARCH
Ctrl_1936	1	Control	SEARCH
PD30549a	0.94855643	Control	SEARCH
Ctrl_1937	1	Control	SEARCH
Ctrl_1938	1	Control	SEARCH
Ctrl_1939	1	Control	SEARCH
PD30560a	1	Control	SEARCH
Ctrl_1940	1	Control	SEARCH

Ctrl_1941	1	Control	SEARCH
Ctrl_1942	1	Control	SEARCH
Ctrl_1943	0.94855643	Control	SEARCH
Ctrl_1944	1	Control	SEARCH
Ctrl_1945	0.948031496	Control	SEARCH
Ctrl_1946	0.94855643	Control	SEARCH
Ctrl_1947	1	Control	SEARCH
Ctrl_1948	0.94855643	Control	SEARCH
Ctrl_1949	1	Control	SEARCH
Ctrl_1950	0.94855643	Control	SEARCH
Ctrl_1951	0.94855643	Control	SEARCH
Ctrl_1952	0.94855643	Control	SEARCH
Ctrl_1953	1	Control	SEARCH
Ctrl_1954	0.94855643	Control	SEARCH
Ctrl_1955	1	Control	SEARCH
Ctrl_1956	1	Control	SEARCH
Ctrl_1957	0.94855643	Control	SEARCH
Ctrl_1958	1	Control	SEARCH
Ctrl_1959	0.94855643	Control	SEARCH
Ctrl_1960	0.94855643	Control	SEARCH
PD30555a	0.94855643	Control	SEARCH
PD30561a	1	Control	SEARCH
Ctrl_1961	1	Control	SEARCH
Ctrl_1962	1	Control	SEARCH
Ctrl_1963	1	Control	SEARCH
Ctrl_1964	1	Control	SEARCH
Ctrl_1965	0.94855643	Control	SEARCH
Ctrl_1966	0.94855643	Control	SEARCH
Ctrl_1967	0.94855643	Control	SEARCH
Ctrl_1968	1	Control	SEARCH
Ctrl_1969	0.94855643	Control	SEARCH
Ctrl_1970	0.94855643	Control	SEARCH
Ctrl_1971	0.94855643	Control	SEARCH
Ctrl_1972	1	Control	SEARCH
Ctrl_1973	1	Control	SEARCH
Ctrl_1974	1	Control	SEARCH
Ctrl_1975	1	Control	SEARCH
Ctrl_1976	1	Control	SEARCH
Ctrl_1977	1	Control	SEARCH
Ctrl_1978	1	Control	SEARCH

Ctrl_1979	1	Control	SEARCH
Ctrl_1980	1	Control	SEARCH
Ctrl_1981	1	Control	SEARCH
Ctrl_1982	0.94855643	Control	SEARCH
Ctrl_1983	1	Control	SEARCH
Ctrl_1984	1	Control	SEARCH
Ctrl_1985	1	Control	SEARCH
Ctrl_1986	1	Control	SEARCH
Ctrl_1987	0.948031496	Control	SEARCH
Ctrl_1988	1	Control	SEARCH
Ctrl_1989	1	Control	SEARCH
Ctrl_1990	1	Control	SEARCH
Ctrl_1991	1	Control	SEARCH
Ctrl_1992	1	Control	SEARCH
Ctrl_1993	0.999475066	Control	SEARCH
Ctrl_1994	1	Control	SEARCH
Ctrl_1995	1	Control	SEARCH
Ctrl_1996	1	Control	SEARCH
Ctrl_1997	1	Control	SEARCH
Ctrl_1998	1	Control	SEARCH
Ctrl_1999	0.94855643	Control	SEARCH
Ctrl_2000	1	Control	SEARCH
Ctrl_2001	1	Control	SEARCH
Ctrl_2002	1	Control	SEARCH
Ctrl_2003	0.94855643	Control	SEARCH
Ctrl_2004	0.94855643	Control	SEARCH
Ctrl_2005	0.94855643	Control	SEARCH
Ctrl_2006	0.94855643	Control	SEARCH
Ctrl_2007	0.94855643	Control	SEARCH
Ctrl_2008	1	Control	SEARCH
Ctrl_2009	1	Control	SEARCH
Ctrl_2010	0.94855643	Control	SEARCH
Ctrl_2011	0.94855643	Control	SEARCH
Ctrl_2012	0.94855643	Control	SEARCH
Ctrl_2013	0.94855643	Control	SEARCH
Ctrl_2014	0.94855643	Control	SEARCH
Ctrl_2015	1	Control	SEARCH
Ctrl_2016	0.94855643	Control	SEARCH
Ctrl_2017	0.94855643	Control	SEARCH
Ctrl_2018	0.94855643	Control	SEARCH

Ctrl_2019	1	Control	SEARCH
Ctrl_2020	1	Control	SEARCH
Ctrl_2021	0.94855643	Control	SEARCH
Ctrl_2022	1	Control	SEARCH
Ctrl_2023	0.94855643	Control	SEARCH
PD30556a	0.94855643	Control	SEARCH
Ctrl_2024	1	Control	SEARCH
Ctrl_2025	1	Control	SEARCH
Ctrl_2026	0.94855643	Control	SEARCH
Ctrl_2027	1	Control	SEARCH
Ctrl_2028	0.94855643	Control	SEARCH
PD30550a	0.94855643	Control	SEARCH
PD30551a	0.94855643	Control	SEARCH
Ctrl_2029	1	Control	SEARCH
Ctrl_2030	0.94855643	Control	SEARCH
Ctrl_2031	0.94855643	Control	SEARCH
Ctrl_2032	1	Control	SEARCH
Ctrl_2033	0.94855643	Control	SEARCH
Ctrl_2034	1	Control	SEARCH
Ctrl_2035	1	Control	SEARCH
Ctrl_2036	1	Control	SEARCH
Ctrl_2037	1	Control	SEARCH
Ctrl_2038	0.94855643	Control	SEARCH
Ctrl_2039	0.94855643	Control	SEARCH
Ctrl_2040	1	Control	SEARCH
Ctrl_2041	1	Control	SEARCH
Ctrl_2042	1	Control	SEARCH
Ctrl_2043	0.940682415	Control	SEARCH
Ctrl_2044	1	Control	SEARCH
Ctrl_2045	1	Control	SEARCH
Ctrl_2046	1	Control	SEARCH
Ctrl_2047	0.94855643	Control	SEARCH
Ctrl_2048	1	Control	SEARCH
Ctrl_2049	1	Control	SEARCH
Ctrl_2050	0.94855643	Control	SEARCH
Ctrl_2051	1	Control	SEARCH
Ctrl_2052	0.94855643	Control	SEARCH
Ctrl_2053	1	Control	SEARCH
Ctrl_2054	0.94855643	Control	SEARCH
Ctrl_2055	1	Control	SEARCH

Ctrl_2056	0.94855643	Control	SEARCH
Ctrl_2057	1	Control	SEARCH
Ctrl_2058	1	Control	SEARCH
Ctrl_2059	1	Control	SEARCH
Ctrl_2060	1	Control	SEARCH
Ctrl_2061	0.94855643	Control	SEARCH
Ctrl_2062	1	Control	SEARCH
Ctrl_2063	0.94855643	Control	SEARCH
Ctrl_2064	1	Control	SEARCH
Ctrl_2065	0.94855643	Control	SEARCH
Ctrl_2066	1	Control	SEARCH
Ctrl_2067	1	Control	SEARCH
Ctrl_2068	1	Control	SEARCH
Ctrl_2069	0.94855643	Control	SEARCH
Ctrl_2070	0.94855643	Control	SEARCH
Ctrl_2071	1	Control	SEARCH
Ctrl_2072	1	Control	SEARCH
Ctrl_2073	1	Control	SEARCH
Ctrl_2074	1	Control	SEARCH
Ctrl_2075	1	Control	SEARCH
Ctrl_2076	1	Control	SEARCH
Ctrl_2077	1	Control	SEARCH
Ctrl_2078	1	Control	SEARCH
Ctrl_2079	1	Control	SEARCH
Ctrl_2080	1	Control	SEARCH
Ctrl_2081	1	Control	SEARCH
Ctrl_2082	1	Control	SEARCH
Ctrl_2083	1	Control	SEARCH
Ctrl_2084	1	Control	SEARCH
Ctrl_2085	1	Control	SEARCH
Ctrl_2086	1	Control	SEARCH
Ctrl_2087	1	Control	SEARCH
Ctrl_2088	1	Control	SEARCH
Ctrl_2089	1	Control	SEARCH
Ctrl_2090	1	Control	SEARCH
Ctrl_2091	1	Control	SEARCH
Ctrl_2092	1	Control	SEARCH
Ctrl_2093	1	Control	SEARCH
Ctrl_2094	1	Control	SEARCH
Ctrl_2095	1	Control	SEARCH

Ctrl_2096	1	Control	SEARCH
Ctrl_2097	1	Control	SEARCH
Ctrl_2098	1	Control	SEARCH
Ctrl_2099	1	Control	SEARCH
Ctrl_2100	1	Control	SEARCH
Ctrl_2101	1	Control	SEARCH
Ctrl_2102	1	Control	SEARCH
Ctrl_2103	1	Control	SEARCH
Ctrl_2104	1	Control	SEARCH
Ctrl_2105	1	Control	SEARCH
Ctrl_2106	1	Control	SEARCH
Ctrl_2107	1	Control	SEARCH
Ctrl_2108	1	Control	SEARCH
Ctrl_2109	1	Control	SEARCH
Ctrl_2110	1	Control	SEARCH
Ctrl_2111	1	Control	SEARCH
Ctrl_2112	1	Control	SEARCH
Ctrl_2113	1	Control	SEARCH
Ctrl_2114	1	Control	SEARCH
Ctrl_2115	1	Control	SEARCH
Ctrl_2116	1	Control	SEARCH
Ctrl_2117	1	Control	SEARCH
Ctrl_2118	1	Control	SEARCH
Ctrl_2119	1	Control	SEARCH
Ctrl_2120	1	Control	SEARCH
Ctrl_2121	1	Control	SEARCH
Ctrl_2122	1	Control	SEARCH
Ctrl_2123	1	Control	SEARCH
Ctrl_2124	1	Control	SEARCH
Ctrl_2125	1	Control	SEARCH
Ctrl_2126	1	Control	SEARCH
Ctrl_2127	1	Control	SEARCH
Ctrl_2128	1	Control	SEARCH
Ctrl_2129	1	Control	SEARCH
Ctrl_2130	1	Control	SEARCH
Ctrl_2131	1	Control	SEARCH
Ctrl_2132	1	Control	SEARCH
Ctrl_2133	1	Control	SEARCH
Ctrl_2134	1	Control	SEARCH
Ctrl_2135	1	Control	SEARCH

Ctrl_2136	1	Control	SEARCH
Ctrl_2137	1	Control	SEARCH
Ctrl_2138	1	Control	SEARCH
Ctrl_2139	1	Control	SEARCH
Ctrl_2140	1	Control	SEARCH
Ctrl_2141	1	Control	SEARCH
Ctrl_2142	1	Control	SEARCH
Ctrl_2143	1	Control	SEARCH
Ctrl_2144	1	Control	SEARCH
Ctrl_2145	1	Control	SEARCH
Ctrl_2146	1	Control	SEARCH
Ctrl_2147	1	Control	SEARCH
Ctrl_2148	1	Control	SEARCH
Ctrl_2149	1	Control	SEARCH
Ctrl_2150	1	Control	SEARCH
Ctrl_2151	1	Control	SEARCH
Ctrl_2152	1	Control	SEARCH
Ctrl_2153	1	Control	SEARCH
Ctrl_2154	1	Control	SEARCH
Ctrl_2155	1	Control	SEARCH
Ctrl_2156	1	Control	SEARCH
Ctrl_2157	1	Control	SEARCH
Ctrl_2158	1	Control	SEARCH
Ctrl_2159	1	Control	SEARCH
Ctrl_2160	1	Control	SEARCH
Ctrl_2161	1	Control	SEARCH
Ctrl_2162	1	Control	SEARCH
Ctrl_2163	1	Control	SEARCH
Ctrl_2164	1	Control	SEARCH
Ctrl_2165	1	Control	SEARCH
Ctrl_2166	1	Control	SEARCH
Ctrl_2167	1	Control	SEARCH
Ctrl_2168	1	Control	SEARCH
Ctrl_2169	1	Control	SEARCH
Ctrl_2170	1	Control	SEARCH
Ctrl_2171	1	Control	SEARCH
Ctrl_2172	1	Control	SEARCH
Ctrl_2173	1	Control	SEARCH
PD30567a	1	Control	SEARCH
Ctrl_2174	1	Control	SEARCH

Ctrl_2175	1	Control	SEARCH
Ctrl_2176	1	Control	SEARCH
Ctrl_2177	1	Control	SEARCH
Ctrl_2178	1	Control	SEARCH
Ctrl_2179	1	Control	SEARCH
Ctrl_2180	1	Control	SEARCH
Ctrl_2181	1	Control	SEARCH
Ctrl_2182	1	Control	SEARCH
Ctrl_2183	1	Control	SEARCH
Ctrl_2184	1	Control	SEARCH
Ctrl_2185	1	Control	SEARCH
Ctrl_2186	1	Control	SEARCH
Ctrl_2187	1	Control	SEARCH
Ctrl_2188	1	Control	SEARCH
Ctrl_2189	1	Control	SEARCH
Ctrl_2190	1	Control	SEARCH
Ctrl_2191	1	Control	SEARCH
Ctrl_2192	1	Control	SEARCH
Ctrl_2193	1	Control	SEARCH
Ctrl_2194	1	Control	SEARCH
Ctrl_2195	1	Control	SEARCH
Ctrl_2196	1	Control	SEARCH
Ctrl_2197	1	Control	SEARCH
Ctrl_2198	1	Control	SEARCH
Ctrl_2199	1	Control	SEARCH
Ctrl_2200	1	Control	SEARCH
Ctrl_2201	1	Control	SEARCH
Ctrl_2202	1	Control	SEARCH
Ctrl_2203	1	Control	SEARCH
Ctrl_2204	0.993700787	Control	SEARCH
Ctrl_2205	1	Control	SEARCH
Ctrl_2206	1	Control	SEARCH
Ctrl_2207	1	Control	SEARCH
Ctrl_2208	1	Control	SEARCH
Ctrl_2209	1	Control	SEARCH
Ctrl_2210	1	Control	SEARCH
Ctrl_2211	1	Control	SEARCH
Ctrl_2212	1	Control	SEARCH
Ctrl_2213	1	Control	SEARCH
Ctrl_2214	1	Control	SEARCH

Ctrl_2215	1	Control	SEARCH
Ctrl_2216	1	Control	SEARCH
Ctrl_2217	1	Control	SEARCH
Ctrl_2218	1	Control	SEARCH
Ctrl_2219	1	Control	SEARCH
Ctrl_2220	1	Control	SEARCH
Ctrl_2221	1	Control	SEARCH
Ctrl_2222	1	Control	SEARCH
Ctrl_2223	1	Control	SEARCH
Ctrl_2224	1	Control	SEARCH
Ctrl_2225	1	Control	SEARCH
Ctrl_2226	1	Control	SEARCH
Ctrl_2227	1	Control	SEARCH
Ctrl_2228	1	Control	SEARCH
Ctrl_2229	1	Control	SEARCH
Ctrl_2230	1	Control	SEARCH
Ctrl_2231	1	Control	SEARCH
Ctrl_2232	1	Control	SEARCH
Ctrl_2233	1	Control	SEARCH
Ctrl_2234	1	Control	SEARCH
Ctrl_2235	1	Control	SEARCH
Ctrl_2236	1	Control	SEARCH
Ctrl_2237	1	Control	SEARCH
Ctrl_2238	1	Control	SEARCH
Ctrl_2239	1	Control	SEARCH
Ctrl_2240	1	Control	SEARCH
Ctrl_2241	1	Control	SEARCH
Ctrl_2242	1	Control	SEARCH
Ctrl_2243	1	Control	SEARCH
Ctrl_2244	1	Control	SEARCH
Ctrl_2245	1	Control	SEARCH
Ctrl_2246	1	Control	SEARCH
Ctrl_2247	1	Control	SEARCH
Ctrl_2248	1	Control	SEARCH
Ctrl_2249	1	Control	SEARCH
Ctrl_2250	1	Control	SEARCH
Ctrl_2251	0.995275591	Control	SEARCH
Ctrl_2252	1	Control	SEARCH
Ctrl_2253	1	Control	SEARCH
Ctrl_2254	1	Control	SEARCH

Ctrl_2255	1	Control	SEARCH
Ctrl_2256	1	Control	SEARCH
Ctrl_2257	1	Control	SEARCH
Ctrl_2258	1	Control	SEARCH
Ctrl_2259	1	Control	SEARCH
Ctrl_2260	1	Control	SEARCH
Ctrl_2261	1	Control	SEARCH
Ctrl_2262	1	Control	SEARCH
Ctrl_2263	1	Control	SEARCH
Ctrl_2264	1	Control	SEARCH
Ctrl_2265	1	Control	SEARCH
Ctrl_2266	1	Control	SEARCH
Ctrl_2267	1	Control	SEARCH
Ctrl_2268	1	Control	SEARCH
Ctrl_2269	1	Control	SEARCH
Ctrl_2270	1	Control	SEARCH
Ctrl_2271	1	Control	SEARCH
Ctrl_2272	1	Control	SEARCH
Ctrl_2273	1	Control	SEARCH
Ctrl_2274	1	Control	SEARCH
Ctrl_2275	1	Control	SEARCH
Ctrl_2276	1	Control	SEARCH
Ctrl_2277	1	Control	SEARCH
Ctrl_2278	1	Control	SEARCH
Ctrl_2279	0.997900262	Control	SEARCH
Ctrl_2280	1	Control	SEARCH
Ctrl_2281	1	Control	SEARCH
Ctrl_2282	1	Control	SEARCH
Ctrl_2283	1	Control	SEARCH
Ctrl_2284	1	Control	SEARCH
Ctrl_2285	1	Control	SEARCH
Ctrl_2286	1	Control	SEARCH
Ctrl_2287	1	Control	SEARCH
Ctrl_2288	1	Control	SEARCH
Ctrl_2289	1	Control	SEARCH
Ctrl_2290	1	Control	SEARCH
Ctrl_2291	1	Control	SEARCH
Ctrl_2292	1	Control	SEARCH
Ctrl_2293	1	Control	SEARCH
Ctrl_2294	1	Control	SEARCH

Ctrl_2295	1	Control	SEARCH
Ctrl_2296	1	Control	SEARCH
Ctrl_2297	1	Control	SEARCH
Ctrl_2298	1	Control	SEARCH
Ctrl_2299	1	Control	SEARCH
Ctrl_2300	1	Control	SEARCH
Ctrl_2301	1	Control	SEARCH
Ctrl_2302	1	Control	SEARCH
Ctrl_2303	1	Control	SEARCH
PD30568a	1	Control	SEARCH
PD30569a	0.999475066	Control	SEARCH
Ctrl_2304	1	Control	SEARCH
Ctrl_2305	1	Control	SEARCH
Ctrl_2306	1	Control	SEARCH
Ctrl_2307	1	Control	SEARCH
Ctrl_2308	1	Control	SEARCH
Ctrl_2309	1	Control	SEARCH
Ctrl_2310	1	Control	SEARCH
Ctrl_2311	1	Control	SEARCH
Ctrl_2312	1	Control	SEARCH
Ctrl_2313	1	Control	SEARCH
Ctrl_2314	1	Control	SEARCH
Ctrl_2315	1	Control	SEARCH
Ctrl_2316	1	Control	SEARCH
Ctrl_2317	1	Control	SEARCH
Ctrl_2318	1	Control	SEARCH
Ctrl_2319	1	Control	SEARCH
Ctrl_2320	1	Control	SEARCH
Ctrl_2321	1	Control	SEARCH
Ctrl_2322	1	Control	SEARCH
Ctrl_2323	1	Control	SEARCH
Ctrl_2324	1	Control	SEARCH
Ctrl_2325	1	Control	SEARCH
Ctrl_2326	1	Control	SEARCH
Ctrl_2327	1	Control	SEARCH
Ctrl_2328	1	Control	SEARCH
Ctrl_2329	1	Control	SEARCH
Ctrl_2330	1	Control	SEARCH
Ctrl_2331	1	Control	SEARCH
Ctrl_2332	1	Control	SEARCH

Ctrl_2333	1	Control	SEARCH
Ctrl_2334	1	Control	SEARCH
Ctrl_2335	1	Control	SEARCH
Ctrl_2336	1	Control	SEARCH
Ctrl_2337	1	Control	SEARCH
Ctrl_2338	1	Control	SEARCH
Ctrl_2339	1	Control	SEARCH
Ctrl_2340	1	Control	SEARCH
Ctrl_2341	1	Control	SEARCH
Ctrl_2342	1	Control	SEARCH
Ctrl_2343	1	Control	SEARCH
Ctrl_2344	1	Control	SEARCH
Ctrl_2345	1	Control	SEARCH
PD30570a	1	Control	SEARCH
Ctrl_2346	1	Control	SEARCH
Ctrl_2347	1	Control	SEARCH
Ctrl_2348	1	Control	SEARCH
Ctrl_2349	1	Control	SEARCH
Ctrl_2350	1	Control	SEARCH
Ctrl_2351	1	Control	SEARCH
Ctrl_2352	1	Control	SEARCH
Ctrl_2353	1	Control	SEARCH
Ctrl_2354	1	Control	SEARCH
Ctrl_2355	1	Control	SEARCH
Ctrl_2356	1	Control	SEARCH
Ctrl_2357	1	Control	SEARCH
Ctrl_2358	1	Control	SEARCH
Ctrl_2359	1	Control	SEARCH
Ctrl_2360	1	Control	SEARCH
Ctrl_2361	1	Control	SEARCH
Ctrl_2362	1	Control	SEARCH
PD30571a	1	Control	SEARCH
Ctrl_2363	1	Control	SEARCH
Ctrl_2364	1	Control	SEARCH
Ctrl_2365	1	Control	SEARCH
Ctrl_2366	1	Control	SEARCH
Ctrl_2367	1	Control	SEARCH
Ctrl_2368	1	Control	SEARCH
Ctrl_2369	1	Control	SEARCH
Ctrl_2370	1	Control	SEARCH

Ctrl_2371	1	Control	SEARCH
Ctrl_2372	1	Control	SEARCH
Ctrl_2373	1	Control	SEARCH
Ctrl_2374	1	Control	SEARCH
Ctrl_2375	1	Control	SEARCH
Ctrl_2376	1	Control	SEARCH
Ctrl_2377	0.998950131	Control	SEARCH
Ctrl_2378	1	Control	SEARCH
Ctrl_2379	1	Control	SEARCH
Ctrl_2380	1	Control	SEARCH
Ctrl_2381	1	Control	SEARCH
Ctrl_2382	1	Control	SEARCH
Ctrl_2383	1	Control	SEARCH
Ctrl_2384	1	Control	SEARCH
Ctrl_2385	1	Control	SEARCH
Ctrl_2386	1	Control	SEARCH
Ctrl_2387	1	Control	SEARCH
Ctrl_2388	1	Control	SEARCH
Ctrl_2389	1	Control	SEARCH
Ctrl_2390	1	Control	SEARCH
Ctrl_2391	1	Control	SEARCH
Ctrl_2392	1	Control	SEARCH
Ctrl_2393	1	Control	SEARCH
Ctrl_2394	1	Control	SEARCH
Ctrl_2395	1	Control	SEARCH
Ctrl_2396	1	Control	SEARCH
Ctrl_2397	1	Control	SEARCH
Ctrl_2398	1	Control	SEARCH
Ctrl_2399	1	Control	SEARCH
Ctrl_2400	1	Control	SEARCH
Ctrl_2401	1	Control	SEARCH
Ctrl_2402	1	Control	SEARCH
Ctrl_2403	1	Control	SEARCH
Ctrl_2404	1	Control	SEARCH
Ctrl_2405	1	Control	SEARCH
Ctrl_2406	1	Control	SEARCH
Ctrl_2407	1	Control	SEARCH
Ctrl_2408	1	Control	SEARCH
Ctrl_2409	1	Control	SEARCH
Ctrl_2410	1	Control	SEARCH

Ctrl_2411	1	Control	SEARCH
Ctrl_2412	1	Control	SEARCH
Ctrl_2413	1	Control	SEARCH
Ctrl_2414	1	Control	SEARCH
Ctrl_2415	1	Control	SEARCH
Ctrl_2416	1	Control	SEARCH
Ctrl_2417	1	Control	SEARCH
Ctrl_2418	1	Control	SEARCH
Ctrl_2419	1	Control	SEARCH
Ctrl_2420	1	Control	SEARCH
Ctrl_2421	1	Control	SEARCH
Ctrl_2422	1	Control	SEARCH
Ctrl_2423	1	Control	SEARCH
Ctrl_2424	1	Control	SEARCH
Ctrl_2425	1	Control	SEARCH
Ctrl_2426	1	Control	SEARCH
Ctrl_2427	1	Control	SEARCH
Ctrl_2428	1	Control	SEARCH
Ctrl_2429	1	Control	SEARCH
Ctrl_2430	1	Control	SEARCH
Ctrl_2431	1	Control	SEARCH
Ctrl_2432	1	Control	SEARCH
Ctrl_2433	1	Control	SEARCH
PD30546a	1	Control	SEARCH
Ctrl_2434	0.94855643	Control	SEARCH
Ctrl_2435	0.94855643	Control	SEARCH
Ctrl_2436	1	Control	SEARCH
Ctrl_2437	0.94855643	Control	SEARCH
Ctrl_2438	0.94855643	Control	SEARCH
PD30574a	1	Control	SEARCH
Ctrl_2439	1	Control	SEARCH
Ctrl_2440	1	Control	SEARCH
Ctrl_2441	1	Control	SEARCH
Ctrl_2442	1	Control	SEARCH
Ctrl_2443	1	Control	SEARCH
Ctrl_2444	1	Control	SEARCH
Ctrl_2445	1	Control	SEARCH
Ctrl_2446	1	Control	SEARCH
Ctrl_2447	1	Control	SEARCH
Ctrl_2448	1	Control	SEARCH

Ctrl_2449	1	Control	SEARCH
Ctrl_2450	1	Control	SEARCH
Ctrl_2451	1	Control	SEARCH
Ctrl_2452	1	Control	SEARCH
Ctrl_2453	1	Control	SEARCH
Ctrl_2454	1	Control	SEARCH
Ctrl_2455	1	Control	SEARCH
Ctrl_2456	1	Control	SEARCH
Ctrl_2457	1	Control	SEARCH
Ctrl_2458	1	Control	SEARCH
Ctrl_2459	1	Control	SEARCH
Ctrl_2460	1	Control	SEARCH
Ctrl_2461	1	Control	SEARCH
Ctrl_2462	1	Control	SEARCH
Ctrl_2463	1	Control	SEARCH
PD30562a	1	Control	SEARCH
Ctrl_2464	1	Control	SEARCH
Ctrl_2465	1	Control	SEARCH
Ctrl_2466	1	Control	SEARCH
Ctrl_2467	1	Control	SEARCH
Ctrl_2468	1	Control	SEARCH
Ctrl_2469	1	Control	SEARCH
Ctrl_2470	1	Control	SEARCH
Ctrl_2471	1	Control	SEARCH
Ctrl_2472	1	Control	SEARCH
Ctrl_2473	1	Control	SEARCH
Ctrl_2474	1	Control	SEARCH
Ctrl_2475	1	Control	SEARCH
Ctrl_2476	1	Control	SEARCH
Ctrl_2477	1	Control	SEARCH
PD30563a	1	Control	SEARCH
Ctrl_2478	1	Control	SEARCH
Ctrl_2479	1	Control	SEARCH
Ctrl_2480	1	Control	SEARCH
Ctrl_2481	1	Control	SEARCH
Ctrl_2482	1	Control	SEARCH
Ctrl_2483	1	Control	SEARCH
Ctrl_2484	1	Control	SEARCH
Ctrl_2485	1	Control	SEARCH
Ctrl_2486	1	Control	SEARCH

Ctrl_2487	1	Control	SEARCH
Ctrl_2488	1	Control	SEARCH
Ctrl_2489	1	Control	SEARCH
Ctrl_2490	1	Control	SEARCH
Ctrl_2491	1	Control	SEARCH
Ctrl_2492	1	Control	SEARCH
PD30564a	1	Control	SEARCH
Ctrl_2493	1	Control	SEARCH
Ctrl_2494	1	Control	SEARCH
Ctrl_2495	1	Control	SEARCH
Ctrl_2496	1	Control	SEARCH
Ctrl_2497	1	Control	SEARCH
Ctrl_2498	1	Control	SEARCH
Ctrl_2499	1	Control	SEARCH
PD30565a	1	Control	SEARCH
Ctrl_2500	1	Control	SEARCH
Ctrl_2501	1	Control	SEARCH
Ctrl_2502	1	Control	SEARCH
Ctrl_2503	1	Control	SEARCH
Ctrl_2504	1	Control	SEARCH
Ctrl_2505	1	Control	SEARCH
Ctrl_2506	1	Control	SEARCH
Ctrl_2507	1	Control	SEARCH
Ctrl_2508	1	Control	SEARCH
Ctrl_2509	1	Control	SEARCH
Ctrl_2510	1	Control	SEARCH
Ctrl_2511	1	Control	SEARCH
Ctrl_2512	1	Control	SEARCH
Ctrl_2513	1	Control	SEARCH
Ctrl_2514	1	Control	SEARCH
Ctrl_2515	1	Control	SEARCH
Ctrl_2516	1	Control	SEARCH
Ctrl_2517	1	Control	SEARCH
Ctrl_2518	1	Control	SEARCH
Ctrl_2519	1	Control	SEARCH
Ctrl_2520	1	Control	SEARCH
Ctrl_2521	1	Control	SEARCH
Ctrl_2522	1	Control	SEARCH
Ctrl_2523	1	Control	SEARCH
Ctrl_2524	1	Control	SEARCH

Ctrl_2525	1	Control	SEARCH
Ctrl_2526	1	Control	SEARCH
Ctrl_2527	1	Control	SEARCH
Ctrl_2528	1	Control	SEARCH
Ctrl_2529	1	Control	SEARCH
Ctrl_2530	1	Control	SEARCH
Ctrl_2531	1	Control	SEARCH
Ctrl_2532	1	Control	SEARCH
Ctrl_2533	1	Control	SEARCH
Ctrl_2534	1	Control	SEARCH
Ctrl_2535	1	Control	SEARCH
Ctrl_2536	1	Control	SEARCH
Ctrl_2537	1	Control	SEARCH
Ctrl_2538	1	Control	SEARCH
Ctrl_2539	1	Control	SEARCH
Ctrl_2540	0.999475066	Control	SEARCH
Ctrl_2541	1	Control	SEARCH
Ctrl_2542	1	Control	SEARCH
Ctrl_2543	1	Control	SEARCH
Ctrl_2544	1	Control	SEARCH
Ctrl_2545	1	Control	SEARCH
Ctrl_2546	1	Control	SEARCH
Ctrl_2547	1	Control	SEARCH
Ctrl_2548	1	Control	SEARCH
Ctrl_2549	1	Control	SEARCH
Ctrl_2550	1	Control	SEARCH
Ctrl_2551	1	Control	SEARCH
Ctrl_2552	1	Control	SEARCH
Ctrl_2553	1	Control	SEARCH
Ctrl_2554	1	Control	SEARCH
Ctrl_2555	1	Control	SEARCH
Ctrl_2556	1	Control	SEARCH
Ctrl_2557	1	Control	SEARCH
Ctrl_2558	1	Control	SEARCH
Ctrl_2559	1	Control	SEARCH
Ctrl_2560	1	Control	SEARCH
Ctrl_2561	1	Control	SEARCH
Ctrl_2562	1	Control	SEARCH
Ctrl_2563	1	Control	SEARCH
Ctrl_2564	1	Control	SEARCH

Ctrl_2565	1	Control	SEARCH
Ctrl_2566	1	Control	SEARCH
Ctrl_2567	1	Control	SEARCH
Ctrl_2568	1	Control	SEARCH
Ctrl_2569	1	Control	SEARCH
Ctrl_2570	1	Control	SEARCH
Ctrl_2571	1	Control	SEARCH
Ctrl_2572	1	Control	SEARCH
Ctrl_2573	1	Control	SEARCH
Ctrl_2574	1	Control	SEARCH
Ctrl_2575	1	Control	SEARCH
Ctrl_2576	1	Control	SEARCH
PD30572a	1	Control	SEARCH
Ctrl_2577	1	Control	SEARCH
Ctrl_2578	1	Control	SEARCH
Ctrl_2579	1	Control	SEARCH
Ctrl_2580	1	Control	SEARCH
Ctrl_2581	1	Control	SEARCH
Ctrl_2582	1	Control	SEARCH
Ctrl_2583	1	Control	SEARCH
Ctrl_2584	1	Control	SEARCH
Ctrl_2585	1	Control	SEARCH
Ctrl_2586	1	Control	SEARCH
Ctrl_2587	1	Control	SEARCH
Ctrl_2588	1	Control	SEARCH
Ctrl_2589	1	Control	SEARCH
Ctrl_2590	1	Control	SEARCH
Ctrl_2591	1	Control	SEARCH
Ctrl_2592	1	Control	SEARCH
Ctrl_2593	1	Control	SEARCH
Ctrl_2594	1	Control	SEARCH
Ctrl_2595	1	Control	SEARCH
Ctrl_2596	1	Control	SEARCH
Ctrl_2597	1	Control	SEARCH
Ctrl_2598	1	Control	SEARCH
Ctrl_2599	1	Control	SEARCH
Ctrl_2600	1	Control	SEARCH
Ctrl_2601	1	Control	SEARCH
Ctrl_2602	1	Control	SEARCH
Ctrl_2603	1	Control	SEARCH

Ctrl_2604	1	Control	SEARCH
Ctrl_2605	1	Control	SEARCH
Ctrl_2606	1	Control	SEARCH
Ctrl_2607	1	Control	SEARCH
Ctrl_2608	1	Control	SEARCH
Ctrl_2609	1	Control	SEARCH
Ctrl_2610	1	Control	SEARCH
Ctrl_2611	1	Control	SEARCH
Ctrl_2612	1	Control	SEARCH
Ctrl_2613	1	Control	SEARCH
Ctrl_2614	1	Control	SEARCH
Ctrl_2615	1	Control	SEARCH
Ctrl_2616	1	Control	SEARCH
Ctrl_2617	1	Control	SEARCH
Ctrl_2618	1	Control	SEARCH
Ctrl_2619	1	Control	SEARCH
Ctrl_2620	1	Control	SEARCH
Ctrl_2621	1	Control	SEARCH
Ctrl_2622	1	Control	SEARCH
PD30573a	1	Control	SEARCH
Ctrl_2623	1	Control	SEARCH
Ctrl_2624	1	Control	SEARCH
Ctrl_2625	1	Control	SEARCH
Ctrl_2626	1	Control	SEARCH
Ctrl_2627	1	Control	SEARCH
Ctrl_2628	1	Control	SEARCH
Ctrl_2629	1	Control	SEARCH
Ctrl_2630	0.998950131	Control	SEARCH
Ctrl_2631	1	Control	SEARCH
Ctrl_2632	1	Control	SEARCH
Ctrl_2633	1	Control	SEARCH
Ctrl_2634	1	Control	SEARCH
Ctrl_2635	1	Control	SEARCH
Ctrl_2636	0.999475066	Control	SEARCH
Ctrl_2637	1	Control	SEARCH
Ctrl_2638	1	Control	SEARCH
Ctrl_2639	1	Control	SEARCH
Ctrl_2640	1	Control	SEARCH
Ctrl_2641	1	Control	SEARCH
Ctrl_2642	1	Control	SEARCH

Ctrl_2643	1	Control	SEARCH
Ctrl_2644	1	Control	SEARCH
Ctrl_2645	1	Control	SEARCH
Ctrl_2646	1	Control	SEARCH
Ctrl_2647	1	Control	SEARCH
Ctrl_2648	1	Control	SEARCH
Ctrl_2649	1	Control	SEARCH
Ctrl_2650	1	Control	SEARCH
Ctrl_2651	1	Control	SEARCH
Ctrl_2652	1	Control	SEARCH
Ctrl_2653	1	Control	SEARCH
Ctrl_2654	1	Control	SEARCH
Ctrl_2655	1	Control	SEARCH
Ctrl_2656	1	Control	SEARCH
Ctrl_2657	1	Control	SEARCH
Ctrl_2658	1	Control	SEARCH
Ctrl_2659	1	Control	SEARCH
Ctrl_2660	1	Control	SEARCH
Ctrl_2661	1	Control	SEARCH
Ctrl_2662	1	Control	SEARCH
Ctrl_2663	1	Control	SEARCH
Ctrl_2664	1	Control	SEARCH
Ctrl_2665	1	Control	SEARCH
Ctrl_2666	1	Control	SEARCH
Ctrl_2667	1	Control	SEARCH
Ctrl_2668	1	Control	SEARCH
Ctrl_2669	1	Control	SEARCH
Ctrl_2670	1	Control	SEARCH
Ctrl_2671	1	Control	SEARCH
Ctrl_2672	1	Control	SEARCH
Ctrl_2673	1	Control	SEARCH
Ctrl_2674	1	Control	SEARCH
Ctrl_2675	1	Control	SEARCH
Ctrl_2676	1	Control	SEARCH
Ctrl_2677	1	Control	SEARCH
Ctrl_2678	1	Control	SEARCH
Ctrl_2679	1	Control	SEARCH
Ctrl_2680	1	Control	SEARCH
Ctrl_2681	1	Control	SEARCH
Ctrl_2682	1	Control	SEARCH

Ctrl_2683	1	Control	SEARCH
Ctrl_2684	1	Control	SEARCH
Ctrl_2685	1	Control	SEARCH
Ctrl_2686	1	Control	SEARCH
Ctrl_2687	1	Control	SEARCH
Ctrl_2688	1	Control	SEARCH
Ctrl_2689	1	Control	SEARCH
Ctrl_2690	1	Control	SEARCH
Ctrl_2691	1	Control	SEARCH
Ctrl_2692	1	Control	SEARCH
Ctrl_2693	1	Control	SEARCH
Ctrl_2694	1	Control	SEARCH
Ctrl_2695	1	Control	SEARCH
Ctrl_2696	1	Control	SEARCH
Ctrl_2697	1	Control	SEARCH
Ctrl_2698	1	Control	SEARCH
Ctrl_2699	1	Control	SEARCH
Ctrl_2700	1	Control	SEARCH
Ctrl_2701	1	Control	SEARCH
Ctrl_2702	1	Control	SEARCH
Ctrl_2703	1	Control	SEARCH
Ctrl_2704	1	Control	SEARCH
Ctrl_2705	1	Control	SEARCH
Ctrl_2706	1	Control	SEARCH
Ctrl_2707	1	Control	SEARCH
Ctrl_2708	1	Control	SEARCH
Ctrl_2709	1	Control	SEARCH
Ctrl_2710	1	Control	SEARCH
Ctrl_2711	1	Control	SEARCH
Ctrl_2712	1	Control	SEARCH
Ctrl_2713	1	Control	SEARCH
Ctrl_2714	1	Control	SEARCH
Ctrl_2715	1	Control	SEARCH
Ctrl_2716	1	Control	SEARCH
Ctrl_2717	1	Control	SEARCH
Ctrl_2718	1	Control	SEARCH
Ctrl_2719	1	Control	SEARCH
Ctrl_2720	1	Control	SEARCH
Ctrl_2721	1	Control	SEARCH
Ctrl_2722	1	Control	SEARCH

Ctrl_2723	1	Control	SEARCH
Ctrl_2724	1	Control	SEARCH
Ctrl_2725	1	Control	SEARCH
Ctrl_2726	1	Control	SEARCH
Ctrl_2727	1	Control	SEARCH
Ctrl_2728	1	Control	SEARCH
Ctrl_2729	1	Control	SEARCH
Ctrl_2730	1	Control	SEARCH
Ctrl_2731	1	Control	SEARCH
Ctrl_2732	1	Control	SEARCH
Ctrl_2733	1	Control	SEARCH
Ctrl_2734	1	Control	SEARCH
Ctrl_2735	1	Control	SEARCH
Ctrl_2736	1	Control	SEARCH
Ctrl_2737	1	Control	SEARCH
Ctrl_2738	1	Control	SEARCH
Ctrl_2739	1	Control	SEARCH
Ctrl_2740	1	Control	SEARCH
Ctrl_2741	1	Control	SEARCH
Ctrl_2742	1	Control	SEARCH
Ctrl_2743	1	Control	SEARCH
Ctrl_2744	1	Control	SEARCH
Ctrl_2745	1	Control	SEARCH
Ctrl_2746	1	Control	SEARCH
Ctrl_2747	1	Control	SEARCH
Ctrl_2748	1	Control	SEARCH
Ctrl_2749	1	Control	SEARCH
Ctrl_2750	1	Control	SEARCH
Ctrl_2751	1	Control	SEARCH
Ctrl_2752	1	Control	SEARCH
Ctrl_2753	1	Control	SEARCH
Ctrl_2754	1	Control	SEARCH
Ctrl_2755	1	Control	SEARCH
Ctrl_2756	1	Control	SEARCH
PD30575a	1	Control	SEARCH
Ctrl_2757	1	Control	SEARCH
Ctrl_2758	1	Control	SEARCH
Ctrl_2759	1	Control	SEARCH
Ctrl_2760	1	Control	SEARCH
Ctrl_2761	1	Control	SEARCH

Ctrl_2762	1	Control	SEARCH
Ctrl_2763	1	Control	SEARCH
Ctrl_2764	1	Control	SEARCH
Ctrl_2765	1	Control	SEARCH
Ctrl_2766	1	Control	SEARCH
Ctrl_2767	1	Control	SEARCH
Ctrl_2768	1	Control	SEARCH
Ctrl_2769	1	Control	SEARCH
Ctrl_2770	1	Control	SEARCH
Ctrl_2771	1	Control	SEARCH
Ctrl_2772	1	Control	SEARCH
Ctrl_2773	1	Control	SEARCH
Ctrl_2774	1	Control	SEARCH
Ctrl_2775	1	Control	SEARCH
Ctrl_2776	1	Control	SEARCH
Ctrl_2777	1	Control	SEARCH
Ctrl_2778	1	Control	SEARCH
Ctrl_2779	1	Control	SEARCH
Ctrl_2780	1	Control	SEARCH
Ctrl_2781	1	Control	SEARCH
Ctrl_2782	1	Control	SEARCH
Ctrl_2783	1	Control	SEARCH
Ctrl_2784	1	Control	SEARCH
Ctrl_2785	1	Control	SEARCH
Ctrl_2786	1	Control	SEARCH
Ctrl_2787	1	Control	SEARCH
Ctrl_2788	1	Control	SEARCH
Ctrl_2789	1	Control	SEARCH
Ctrl_2790	1	Control	SEARCH
Ctrl_2791	1	Control	SEARCH
Ctrl_2792	1	Control	SEARCH
Ctrl_2793	1	Control	SEARCH
Ctrl_2794	1	Control	SEARCH
Ctrl_2795	1	Control	SEARCH
Ctrl_2796	1	Control	SEARCH
Ctrl_2797	1	Control	SEARCH
Ctrl_2798	1	Control	SEARCH
Ctrl_2799	1	Control	SEARCH
Ctrl_2800	1	Control	SEARCH
Ctrl_2801	1	Control	SEARCH

Ctrl_2802	1	Control	SEARCH
Ctrl_2803	1	Control	SEARCH
Ctrl_2804	1	Control	SEARCH
Ctrl_2805	1	Control	SEARCH
Ctrl_2806	1	Control	SEARCH
Ctrl_2807	1	Control	SEARCH
Ctrl_2808	1	Control	SEARCH
Ctrl_2809	1	Control	SEARCH
Ctrl_2810	1	Control	SEARCH
Ctrl_2811	0.999475066	Control	SEARCH
Ctrl_2812	1	Control	SEARCH
Ctrl_2813	1	Control	SEARCH
Ctrl_2814	1	Control	SEARCH
Ctrl_2815	1	Control	SEARCH
Ctrl_2816	0.95328084	Control	SEARCH
Ctrl_2817	1	Control	SEARCH
Ctrl_2818	1	Control	SEARCH
Ctrl_2819	1	Control	SEARCH
Ctrl_2820	1	Control	SEARCH
Ctrl_2821	1	Control	SEARCH
Ctrl_2822	1	Control	SEARCH
Ctrl_2823	1	Control	SEARCH
Ctrl_2824	1	Control	SEARCH
Ctrl_2825	1	Control	SEARCH
Ctrl_2826	1	Control	SEARCH
Ctrl_2827	1	Control	SEARCH
Ctrl_2828	1	Control	SEARCH
Ctrl_2829	1	Control	SEARCH
Ctrl_2830	1	Control	SEARCH
Ctrl_2831	1	Control	SEARCH
Ctrl_2832	1	Control	SEARCH
Ctrl_2833	1	Control	SEARCH
Ctrl_2834	1	Control	SEARCH
Ctrl_2835	1	Control	SEARCH
Ctrl_2836	1	Control	SEARCH
Ctrl_2837	1	Control	SEARCH
Ctrl_2838	1	Control	SEARCH
Ctrl_2839	1	Control	SEARCH
Ctrl_2840	1	Control	SEARCH
Ctrl_2841	1	Control	SEARCH

Ctrl_2842	1	Control	SEARCH
Ctrl_2843	1	Control	SEARCH
Ctrl_2844	1	Control	SEARCH
Ctrl_2845	1	Control	SEARCH
Ctrl_2846	1	Control	SEARCH
Ctrl_2847	1	Control	SEARCH
Ctrl_2848	1	Control	SEARCH
Ctrl_2849	1	Control	SEARCH
Ctrl_2850	1	Control	SEARCH
Ctrl_2851	1	Control	SEARCH
Ctrl_2852	1	Control	SEARCH
Ctrl_2853	1	Control	SEARCH
Ctrl_2854	1	Control	SEARCH
Ctrl_2855	1	Control	SEARCH
Ctrl_2856	1	Control	SEARCH
Ctrl_2857	1	Control	SEARCH
Ctrl_2858	1	Control	SEARCH
Ctrl_2859	1	Control	SEARCH
Ctrl_2860	1	Control	SEARCH
Ctrl_2861	1	Control	SEARCH
Ctrl_2862	1	Control	SEARCH
Ctrl_2863	1	Control	SEARCH
Ctrl_2864	1	Control	SEARCH
Ctrl_2865	1	Control	SEARCH
Ctrl_2866	1	Control	SEARCH
Ctrl_2867	1	Control	SEARCH
Ctrl_2868	1	Control	SEARCH
Ctrl_2869	1	Control	SEARCH
Ctrl_2870	1	Control	SEARCH
Ctrl_2871	1	Control	SEARCH
Ctrl_2872	1	Control	SEARCH
Ctrl_2873	1	Control	SEARCH
Ctrl_2874	1	Control	SEARCH
Ctrl_2875	1	Control	SEARCH
Ctrl_2876	1	Control	SEARCH
Ctrl_2877	1	Control	SEARCH
Ctrl_2878	1	Control	SEARCH
Ctrl_2879	1	Control	SEARCH
Ctrl_2880	1	Control	SEARCH
Ctrl_2881	1	Control	SEARCH

Ctrl_2882	1	Control	SEARCH
Ctrl_2883	1	Control	SEARCH
Ctrl_2884	1	Control	SEARCH
Ctrl_2885	1	Control	SEARCH
Ctrl_2886	1	Control	SEARCH
Ctrl_2887	1	Control	SEARCH
Ctrl_2888	1	Control	SEARCH
Ctrl_2889	1	Control	SEARCH
Ctrl_2890	1	Control	SEARCH
Ctrl_2891	1	Control	SEARCH
Ctrl_2892	1	Control	SEARCH
Ctrl_2893	1	Control	SEARCH
Ctrl_2894	1	Control	SEARCH
Ctrl_2895	1	Control	SEARCH
Ctrl_2896	1	Control	SEARCH
Ctrl_2897	1	Control	SEARCH
Ctrl_2898	1	Control	SEARCH
Ctrl_2899	1	Control	SEARCH
Ctrl_2900	1	Control	SEARCH
Ctrl_2901	1	Control	SEARCH
Ctrl_2902	1	Control	SEARCH
Ctrl_2903	1	Control	SEARCH
Ctrl_2904	1	Control	SEARCH
Ctrl_2905	1	Control	SEARCH
Ctrl_2906	1	Control	SEARCH
Ctrl_2907	1	Control	SEARCH
Ctrl_2908	1	Control	SEARCH
Ctrl_2909	1	Control	SEARCH
Ctrl_2910	1	Control	SEARCH
Ctrl_2911	0.940682415	Control	SEARCH
Ctrl_2912	1	Control	SEARCH
Ctrl_2913	1	Control	SEARCH
Ctrl_2914	1	Control	SEARCH
Ctrl_2915	1	Control	SEARCH
Ctrl_2916	1	Control	SEARCH
Ctrl_2917	1	Control	SEARCH
Ctrl_2918	1	Control	SEARCH
Ctrl_2919	1	Control	SEARCH
Ctrl_2920	1	Control	SEARCH
Ctrl_2921	1	Control	SEARCH

Ctrl_2922	1	Control	SEARCH
Ctrl_2923	1	Control	SEARCH
Ctrl_2924	1	Control	SEARCH
Ctrl_2925	1	Control	SEARCH
Ctrl_2926	0.998950131	Control	SEARCH
Ctrl_2927	1	Control	SEARCH
Ctrl_2928	1	Control	SEARCH
Ctrl_2929	1	Control	SEARCH
Ctrl_2930	1	Control	SEARCH
Ctrl_2931	1	Control	SEARCH
Ctrl_2932	1	Control	SEARCH
Ctrl_2933	1	Control	SEARCH
Ctrl_2934	1	Control	SEARCH
Ctrl_2935	1	Control	SEARCH
Ctrl_2936	1	Control	SEARCH
Ctrl_2937	1	Control	SEARCH
Ctrl_2938	1	Control	SEARCH
Ctrl_2939	1	Control	SEARCH
Ctrl_2940	1	Control	SEARCH
Ctrl_2941	1	Control	SEARCH
Ctrl_2942	1	Control	SEARCH
Ctrl_2943	1	Control	SEARCH
Ctrl_2944	1	Control	SEARCH
Ctrl_2945	1	Control	SEARCH
Ctrl_2946	1	Control	SEARCH
Ctrl_2947	1	Control	SEARCH
Ctrl_2948	1	Control	SEARCH
Ctrl_2949	1	Control	SEARCH
Ctrl_2950	1	Control	SEARCH
Ctrl_2951	1	Control	SEARCH
Ctrl_2952	1	Control	SEARCH
Ctrl_2953	1	Control	SEARCH
Ctrl_2954	0.94855643	Control	SEARCH
Ctrl_2955	1	Control	SEARCH
Ctrl_2956	0.94855643	Control	SEARCH
Ctrl_2957	0.94855643	Control	SEARCH
Ctrl_2958	0.94855643	Control	SEARCH
Ctrl_2959	1	Control	SEARCH
Ctrl_2960	1	Control	SEARCH
Ctrl_2961	1	Control	SEARCH

Ctrl_2962	1	Control	SEARCH
Ctrl_2963	1	Control	SEARCH
Ctrl_2964	1	Control	SEARCH
Ctrl_2965	0.999475066	Control	SEARCH
Ctrl_2966	1	Control	SEARCH
Ctrl_2967	1	Control	SEARCH
Ctrl_2968	1	Control	SEARCH
Ctrl_2969	1	Control	SEARCH
Ctrl_2970	1	Control	SEARCH
Ctrl_2971	1	Control	SEARCH
Ctrl_2972	1	Control	SEARCH
Ctrl_2973	1	Control	SEARCH
Ctrl_2974	1	Control	SEARCH
Ctrl_2975	1	Control	SEARCH
Ctrl_2976	1	Control	SEARCH
Ctrl_2977	1	Control	SEARCH
Ctrl_2978	1	Control	SEARCH
Ctrl_2979	1	Control	SEARCH
Ctrl_2980	1	Control	SEARCH
Ctrl_2981	1	Control	SEARCH
Ctrl_2982	1	Control	SEARCH
Ctrl_2983	1	Control	SEARCH
PD30547a	1	Control	SEARCH
Ctrl_2984	1	Control	SEARCH
Ctrl_2985	1	Control	SEARCH
Ctrl_2986	1	Control	SEARCH
Ctrl_2987	1	Control	SEARCH
Ctrl_2988	0.999475066	Control	SEARCH
Ctrl_2989	1	Control	SEARCH
Ctrl_2990	1	Control	SEARCH
Ctrl_2991	1	Control	SEARCH
Ctrl_2992	1	Control	SEARCH
Ctrl_2993	1	Control	SEARCH
Ctrl_2994	1	Control	SEARCH
Ctrl_2995	1	Control	SEARCH
Ctrl_2996	1	Control	SEARCH
Ctrl_2997	1	Control	SEARCH
Ctrl_2998	0.999475066	Control	SEARCH
Ctrl_2999	1	Control	SEARCH
Ctrl_3000	1	Control	SEARCH

Ctrl_3001	1	Control	SEARCH
Ctrl_3002	0.999475066	Control	SEARCH
Ctrl_3003	1	Control	SEARCH
Ctrl_3004	1	Control	SEARCH
Ctrl_3005	1	Control	SEARCH
Ctrl_3006	0.999475066	Control	SEARCH
Ctrl_3007	1	Control	SEARCH
Ctrl_3008	1	Control	SEARCH
Ctrl_3009	1	Control	SEARCH
Ctrl_3010	1	Control	SEARCH
Ctrl_3011	1	Control	SEARCH
Ctrl_3012	1	Control	SEARCH
Ctrl_3013	1	Control	SEARCH
Ctrl_3014	1	Control	SEARCH
Ctrl_3015	1	Control	SEARCH
Ctrl_3016	1	Control	SEARCH
Ctrl_3017	1	Control	SEARCH
Ctrl_3018	1	Control	SEARCH
Ctrl_3019	1	Control	SEARCH
Ctrl_3020	1	Control	SEARCH
Ctrl_3021	1	Control	SEARCH
Ctrl_3022	1	Control	SEARCH
Ctrl_3023	1	Control	SEARCH
Ctrl_3024	1	Control	SEARCH
Ctrl_3025	1	Control	SEARCH
Ctrl_3026	1	Control	SEARCH
Ctrl_3027	1	Control	SEARCH
Ctrl_3028	1	Control	SEARCH
Ctrl_3029	1	Control	SEARCH
Ctrl_3030	1	Control	SEARCH
Ctrl_3031	1	Control	SEARCH
Ctrl_3032	1	Control	SEARCH
Ctrl_3033	1	Control	SEARCH
Ctrl_3034	1	Control	SEARCH
Ctrl_3035	1	Control	SEARCH
Ctrl_3036	1	Control	SEARCH
Ctrl_3037	1	Control	SEARCH
Ctrl_3038	1	Control	SEARCH
Ctrl_3039	1	Control	SEARCH
Ctrl_3040	1	Control	SEARCH

Ctrl_3041	1	Control	SEARCH
Ctrl_3042	1	Control	SEARCH
Ctrl_3043	1	Control	SEARCH
Ctrl_3044	0.940682415	Control	SEARCH
Ctrl_3045	1	Control	SEARCH
Ctrl_3046	1	Control	SEARCH
Ctrl_3047	1	Control	SEARCH
Ctrl_3048	1	Control	SEARCH
Ctrl_3049	1	Control	SEARCH
Ctrl_3050	1	Control	SEARCH
Ctrl_3051	1	Control	SEARCH
Ctrl_3052	1	Control	SEARCH
Ctrl_3053	1	Control	SEARCH
Ctrl_3054	1	Control	SEARCH
Ctrl_3055	1	Control	SEARCH
Ctrl_3056	1	Control	SEARCH
Ctrl_3057	1	Control	SEARCH
Ctrl_3058	1	Control	SEARCH
Ctrl_3059	1	Control	SEARCH
Ctrl_3060	1	Control	SEARCH
Ctrl_3061	1	Control	SEARCH
Ctrl_3062	1	Control	SEARCH
Ctrl_3063	1	Control	SEARCH
Ctrl_3064	1	Control	SEARCH
Ctrl_3065	1	Control	SEARCH
Ctrl_3066	1	Control	SEARCH
Ctrl_3067	1	Control	SEARCH
Ctrl_3068	1	Control	SEARCH
PD30548a	1	Control	SEARCH
Ctrl_3069	1	Control	SEARCH
Ctrl_3070	1	Control	SEARCH
Ctrl_3071	1	Control	SEARCH
Ctrl_3072	1	Control	SEARCH
Ctrl_3073	1	Control	SEARCH
Ctrl_3074	1	Control	SEARCH
Ctrl_3075	1	Control	SEARCH
Ctrl_3076	1	Control	SEARCH
Ctrl_3077	0.94855643	Control	SEARCH
Ctrl_3078	0.94855643	Control	SEARCH
Ctrl_3079	1	Control	SEARCH

PD30557a	0.94855643	Control	SEARCH
Ctrl_3080	0.94855643	Control	SEARCH
Ctrl_3081	1	Control	SEARCH
Ctrl_3082	1	Control	SEARCH
Ctrl_3083	1	Control	SEARCH
Ctrl_3084	0.94855643	Control	SEARCH
Ctrl_3085	0.94855643	Control	SEARCH
Ctrl_3086	0.94855643	Control	SEARCH
Ctrl_3087	0.94855643	Control	SEARCH
Ctrl_3088	1	Control	SEARCH
Ctrl_3089	0.94855643	Control	SEARCH
Ctrl_3090	0.94855643	Control	SEARCH
Ctrl_3091	1	Control	SEARCH
Ctrl_3092	0.94855643	Control	SEARCH
Ctrl_3093	0.94855643	Control	SEARCH
Ctrl_3094	0.94855643	Control	SEARCH
Ctrl_3095	0.94855643	Control	SEARCH
Ctrl_3096	0.94855643	Control	SEARCH
Ctrl_3097	0.94855643	Control	SEARCH
Ctrl_3098	0.94855643	Control	SEARCH
Ctrl_3099	0.94855643	Control	SEARCH
Ctrl_3100	0.94855643	Control	SEARCH
Ctrl_3101	0.94855643	Control	SEARCH
Ctrl_3102	0.94855643	Control	SEARCH
Ctrl_3103	0.94855643	Control	SEARCH
Ctrl_3104	0.94855643	Control	SEARCH
Ctrl_3105	0.94855643	Control	SEARCH
Ctrl_3106	0.94855643	Control	SEARCH
Ctrl_3107	1	Control	SEARCH
Ctrl_3108	1	Control	SEARCH
Ctrl_3109	1	Control	SEARCH
Ctrl_3110	1	Control	SEARCH
Ctrl_3111	1	Control	SEARCH
Ctrl_3112	1	Control	SEARCH
Ctrl_3113	1	Control	SEARCH
Ctrl_3114	1	Control	SEARCH
Ctrl_3115	1	Control	SEARCH
Ctrl_3116	1	Control	SEARCH
Ctrl_3117	1	Control	SEARCH
Ctrl_3118	1	Control	SEARCH

Ctrl_3119	1	Control	SEARCH
Ctrl_3120	1	Control	SEARCH
Ctrl_3121	0.940682415	Control	SEARCH
Ctrl_3122	1	Control	SEARCH
Ctrl_3123	0.94855643	Control	SEARCH
Ctrl_3124	1	Control	SEARCH
Ctrl_3125	0.94855643	Control	SEARCH
Ctrl_3126	0.94855643	Control	SEARCH
Ctrl_3127	1	Control	SEARCH
Ctrl_3128	1	Control	SEARCH
Ctrl_3129	1	Control	SEARCH
Ctrl_3130	1	Control	SEARCH
Ctrl_3131	1	Control	SEARCH
Ctrl_3132	0.948031496	Control	SEARCH
Ctrl_3133	1	Control	SEARCH
PD30552a	0.94855643	Control	SEARCH
Ctrl_3134	1	Control	SEARCH
Ctrl_3135	1	Control	SEARCH
Ctrl_3136	1	Control	SEARCH
Ctrl_3137	0.94855643	Control	SEARCH
Ctrl_3138	0.94855643	Control	SEARCH
Ctrl_3139	0.94855643	Control	SEARCH
Ctrl_3140	0.94855643	Control	SEARCH
Ctrl_3141	0.94855643	Control	SEARCH
Ctrl_3142	0.94855643	Control	SEARCH
Ctrl_3143	0.94855643	Control	SEARCH
Ctrl_3144	0.94855643	Control	SEARCH
Ctrl_3145	0.94855643	Control	SEARCH
Ctrl_3146	0.94855643	Control	SEARCH
Ctrl_3147	0.94855643	Control	SEARCH
Ctrl_3148	0.94855643	Control	SEARCH
Ctrl_3149	0.94855643	Control	SEARCH
Ctrl_3150	0.94855643	Control	SEARCH
Ctrl_3151	0.94855643	Control	SEARCH
Ctrl_3152	0.94855643	Control	SEARCH
Ctrl_3153	0.94855643	Control	SEARCH
Ctrl_3154	0.94855643	Control	SEARCH
Ctrl_3155	0.94855643	Control	SEARCH
Ctrl_3156	0.948031496	Control	SEARCH
Ctrl_3157	0.948031496	Control	SEARCH

PD30553a	0.94855643	Control	SEARCH
Ctrl_3158	0.94855643	Control	SEARCH
Ctrl_3159	0.94855643	Control	SEARCH
Ctrl_3160	0.94855643	Control	SEARCH
Ctrl_3161	0.94855643	Control	SEARCH
Ctrl_3162	0.94855643	Control	SEARCH
Ctrl_3163	0.94855643	Control	SEARCH
Ctrl_3164	0.94855643	Control	SEARCH
Ctrl_3165	0.94855643	Control	SEARCH
Ctrl_3166	0.94855643	Control	SEARCH
Ctrl_3167	0.94855643	Control	SEARCH
Ctrl_3168	0.94855643	Control	SEARCH
Ctrl_3169	0.94855643	Control	SEARCH
Ctrl_3170	0.94855643	Control	SEARCH
Ctrl_3171	0.94855643	Control	SEARCH
Ctrl_3172	0.94855643	Control	SEARCH
Ctrl_3173	0.94855643	Control	SEARCH
Ctrl_3174	0.94855643	Control	SEARCH
Ctrl_3175	0.94855643	Control	SEARCH
Ctrl_3176	0.94855643	Control	SEARCH
Ctrl_3177	0.94855643	Control	SEARCH
Ctrl_3178	0.94855643	Control	SEARCH
Ctrl_3179	0.94855643	Control	SEARCH
Ctrl_3180	0.94855643	Control	SEARCH
Ctrl_3181	1	Control	SEARCH
Ctrl_3182	1	Control	SEARCH
Ctrl_3183	1	Control	SEARCH
Ctrl_3184	1	Control	SEARCH
Ctrl_3185	1	Control	SEARCH
PD30554a	1	Control	SEARCH
Ctrl_3186	1	Control	SEARCH
Ctrl_3187	1	Control	SEARCH
Ctrl_3188	1	Control	SEARCH
Ctrl_3189	1	Control	SEARCH
Ctrl_3190	1	Control	SEARCH
Ctrl_3191	1	Control	SEARCH
Ctrl_3192	1	Control	SEARCH
Ctrl_3193	1	Control	SEARCH
Ctrl_3194	1	Control	SEARCH
Ctrl_3195	1	Control	SEARCH

Ctrl_3196	1	Control	SEARCH
Ctrl_3197	1	Control	SEARCH
Ctrl_3198	1	Control	SEARCH
Ctrl_3199	1	Control	SEARCH
Ctrl_3200	1	Control	SEARCH
Ctrl_3201	1	Control	SEARCH
Ctrl_3202	1	Control	SEARCH
Ctrl_3203	0.94855643	Control	SEARCH
Ctrl_3204	0.94855643	Control	SEARCH
Ctrl_3205	0.94855643	Control	SEARCH
Ctrl_3206	0.94855643	Control	SEARCH
Ctrl_3207	0.94855643	Control	SEARCH
Ctrl_3208	0.94855643	Control	SEARCH
Ctrl_3209	0.94855643	Control	SEARCH
Ctrl_3210	0.94855643	Control	SEARCH
Ctrl_3211	0.94855643	Control	SEARCH
Ctrl_3212	0.94855643	Control	SEARCH
Ctrl_3213	0.94855643	Control	SEARCH
Ctrl_3214	0.94855643	Control	SEARCH
Ctrl_3215	0.94855643	Control	SEARCH
Ctrl_3216	0.94855643	Control	SEARCH
Ctrl_3217	0.94855643	Control	SEARCH
Ctrl_3218	0.94855643	Control	SEARCH
Ctrl_3219	0.94855643	Control	SEARCH
Ctrl_3220	0.94855643	Control	SEARCH
Ctrl_3221	0.94855643	Control	SEARCH
Ctrl_3222	0.94855643	Control	SEARCH
Ctrl_3223	1	Control	SEARCH
Ctrl_3224	0.94855643	Control	SEARCH
Ctrl_3225	1	Control	SEARCH
Ctrl_3226	1	Control	SEARCH
Ctrl_3227	0.94855643	Control	SEARCH
Ctrl_3228	0.94855643	Control	SEARCH
Ctrl_3229	1	Control	SEARCH
PD30566a	1	Control	SEARCH
Ctrl_3230	1	Control	SEARCH
Ctrl_3231	1	Control	SEARCH
Ctrl_3232	1	Control	SEARCH
Ctrl_3233	1	Control	SEARCH
Case_1486	1	Case	SEARCH

Case_1487	1	Case	SEARCH
Case_1488	1	Case	SEARCH
Case_1489	1	Case	SEARCH
PD30727a	1	Case	SEARCH
Case_1490	1	Case	SEARCH
Case_1491	1	Case	SEARCH
Case_1492	1	Case	SEARCH
Case_1493	1	Case	SEARCH
Case_1494	1	Case	SEARCH
Case_1495	1	Case	SEARCH
Case_1496	1	Case	SEARCH
Case_1497	1	Case	SEARCH
Case_1498	1	Case	SEARCH
Case_1499	1	Case	SEARCH
Case_1500	1	Case	SEARCH
Case_1501	1	Case	SEARCH
Case_1502	1	Case	SEARCH
Case_1503	1	Case	SEARCH
Case_1504	1	Case	SEARCH
Case_1505	1	Case	SEARCH
Case_1506	1	Case	SEARCH
Case_1507	1	Case	SEARCH
Case_1508	1	Case	SEARCH
Case_1509	1	Case	SEARCH
Case_1510	1	Case	SEARCH
Case_1511	1	Case	SEARCH
Case_1512	1	Case	SEARCH
Case_1513	1	Case	SEARCH
Case_1514	1	Case	SEARCH
Case_1515	1	Case	SEARCH
PD30728a	1	Case	SEARCH
Case_1516	1	Case	SEARCH
Case_1517	1	Case	SEARCH
Case_1518	1	Case	SEARCH
Case_1519	1	Case	SEARCH
Case_1520	0.999475066	Case	SEARCH
Case_1521	1	Case	SEARCH
Case_1522	1	Case	SEARCH
Case_1523	1	Case	SEARCH
Case_1524	1	Case	SEARCH

Case_1525	1	Case	SEARCH
Case_1526	0.951181102	Case	SEARCH
Case_1527	1	Case	SEARCH
Case_1528	1	Case	SEARCH
Case_1529	1	Case	SEARCH
Case_1530	1	Case	SEARCH
Case_1531	1	Case	SEARCH
Case_1532	1	Case	SEARCH
Case_1533	1	Case	SEARCH
Case_1534	1	Case	SEARCH
Case_1535	1	Case	SEARCH
Case_1536	1	Case	SEARCH
Case_1537	1	Case	SEARCH
Case_1538	1	Case	SEARCH
Case_1539	1	Case	SEARCH
Case_1540	1	Case	SEARCH
Case_1541	1	Case	SEARCH
Case_1542	1	Case	SEARCH
Case_1543	1	Case	SEARCH
Case_1544	1	Case	SEARCH
Case_1545	1	Case	SEARCH
Case_1546	1	Case	SEARCH
Case_1547	1	Case	SEARCH
Case_1548	1	Case	SEARCH
Case_1549	1	Case	SEARCH
Case_1550	1	Case	SEARCH
Case_1551	1	Case	SEARCH
Case_1552	1	Case	SEARCH
Case_1553	1	Case	SEARCH
Case_1554	1	Case	SEARCH
Case_1555	1	Case	SEARCH
Case_1556	1	Case	SEARCH
Case_1557	1	Case	SEARCH
Case_1558	1	Case	SEARCH
Case_1559	1	Case	SEARCH
Case_1560	1	Case	SEARCH
Case_1561	1	Case	SEARCH
Case_1562	1	Case	SEARCH
Case_1563	1	Case	SEARCH
Case_1564	1	Case	SEARCH

Case_1565	1	Case	SEARCH
Case_1566	1	Case	SEARCH
Case_1567	1	Case	SEARCH
Case_1568	1	Case	SEARCH
Case_1569	1	Case	SEARCH
Case_1570	1	Case	SEARCH
Case_1571	1	Case	SEARCH
Case_1572	1	Case	SEARCH
Case_1573	1	Case	SEARCH
Case_1574	1	Case	SEARCH
Case_1575	1	Case	SEARCH
Case_1576	1	Case	SEARCH
Case_1577	1	Case	SEARCH
Case_1578	1	Case	SEARCH
Case_1579	1	Case	SEARCH
Case_1580	1	Case	SEARCH
Case_1581	0.999475066	Case	SEARCH
Case_1582	1	Case	SEARCH
Case_1583	1	Case	SEARCH
Case_1584	1	Case	SEARCH
Case_1585	1	Case	SEARCH
Case_1586	1	Case	SEARCH
Case_1587	1	Case	SEARCH
Case_1588	0.999475066	Case	SEARCH
Case_1589	1	Case	SEARCH
Case_1590	1	Case	SEARCH
Case_1591	1	Case	SEARCH
Case_1592	1	Case	SEARCH
Case_1593	1	Case	SEARCH
Case_1594	1	Case	SEARCH
Case_1595	1	Case	SEARCH
Case_1596	1	Case	SEARCH
Case_1597	1	Case	SEARCH
Case_1598	1	Case	SEARCH
Case_1599	1	Case	SEARCH
Case_1600	0.999475066	Case	SEARCH
Case_1601	1	Case	SEARCH
Case_1602	1	Case	SEARCH
Case_1603	0.999475066	Case	SEARCH
Case_1604	1	Case	SEARCH

Case_1605	1	Case	SEARCH
Case_1606	1	Case	SEARCH
Case_1607	1	Case	SEARCH
Case_1608	1	Case	SEARCH
Case_1609	1	Case	SEARCH
Case_1610	1	Case	SEARCH
Case_1611	1	Case	SEARCH
Case_1612	1	Case	SEARCH
Case_1613	1	Case	SEARCH
Case_1614	1	Case	SEARCH
Case_1615	1	Case	SEARCH
Case_1616	1	Case	SEARCH
Case_1617	1	Case	SEARCH
Case_1618	1	Case	SEARCH
Case_1619	1	Case	SEARCH
PD30716a	1	Case	SEARCH
Case_1620	0.980577428	Case	SEARCH
Case_1621	1	Case	SEARCH
Case_1622	1	Case	SEARCH
Case_1623	1	Case	SEARCH
Case_1624	0.970603675	Case	SEARCH
Case_1625	1	Case	SEARCH
Case_1626	1	Case	SEARCH
Case_1627	1	Case	SEARCH
Case_1628	1	Case	SEARCH
Case_1629	1	Case	SEARCH
Case_1630	1	Case	SEARCH
Case_1631	1	Case	SEARCH
Case_1632	1	Case	SEARCH
Case_1633	1	Case	SEARCH
Case_1634	1	Case	SEARCH
Case_1635	1	Case	SEARCH
Case_1636	1	Case	SEARCH
Case_1637	0.999475066	Case	SEARCH
Case_1638	1	Case	SEARCH
Case_1639	0.982152231	Case	SEARCH
PD30717a	1	Case	SEARCH
Case_1640	0.975328084	Case	SEARCH
PD30729a	1	Case	SEARCH
Case_1641	1	Case	SEARCH

Case_1642	1	Case	SEARCH
Case_1643	1	Case	SEARCH
Case_1644	1	Case	SEARCH
Case_1645	1	Case	SEARCH
Case_1646	1	Case	SEARCH
Case_1647	1	Case	SEARCH
Case_1648	1	Case	SEARCH
Case_1649	0.940682415	Case	SEARCH
Case_1650	0.999475066	Case	SEARCH
Case_1651	1	Case	SEARCH
Case_1652	0.998950131	Case	SEARCH
Case_1653	1	Case	SEARCH
Case_1654	1	Case	SEARCH
Case_1655	1	Case	SEARCH
Case_1656	0.999475066	Case	SEARCH
PD30702a	0.999475066	Case	SEARCH
Case_1657	1	Case	SEARCH
Case_1658	1	Case	SEARCH
Case_1659	0.940682415	Case	SEARCH
Case_1660	1	Case	SEARCH
Case_1661	0.998950131	Case	SEARCH
Case_1662	1	Case	SEARCH
Case_1663	1	Case	SEARCH
Case_1664	0.940682415	Case	SEARCH
Case_1665	1	Case	SEARCH
Case_1666	1	Case	SEARCH
Case_1667	1	Case	SEARCH
Case_1668	0.997375328	Case	SEARCH
Case_1669	1	Case	SEARCH
Case_1670	0.998425197	Case	SEARCH
Case_1671	0.999475066	Case	SEARCH
Case_1672	0.940682415	Case	SEARCH
Case_1673	1	Case	SEARCH
Case_1674	1	Case	SEARCH
Case_1675	1	Case	SEARCH
Case_1676	0.999475066	Case	SEARCH
Case_1677	1	Case	SEARCH
Case_1678	1	Case	SEARCH
PD30703a	0.996850394	Case	SEARCH
Case_1679	0.996325459	Case	SEARCH

Case_1680	1	Case	SEARCH
Case_1681	1	Case	SEARCH
Case_1682	1	Case	SEARCH
Case_1683	1	Case	SEARCH
Case_1684	0.999475066	Case	SEARCH
Case_1685	0.998425197	Case	SEARCH
PD30699a	0.940682415	Case	SEARCH
Case_1686	1	Case	SEARCH
Case_1687	1	Case	SEARCH
Case_1688	1	Case	SEARCH
Case_1689	0.999475066	Case	SEARCH
Case_1690	1	Case	SEARCH
Case_1691	0.940682415	Case	SEARCH
Case_1692	0.997375328	Case	SEARCH
Case_1693	0.940682415	Case	SEARCH
Case_1694	1	Case	SEARCH
Case_1695	0.940682415	Case	SEARCH
Case_1696	1	Case	SEARCH
Case_1697	1	Case	SEARCH
Case_1698	0.998950131	Case	SEARCH
Case_1699	1	Case	SEARCH
Case_1700	0.999475066	Case	SEARCH
Case_1701	0.940682415	Case	SEARCH
Case_1702	0.998425197	Case	SEARCH
Case_1703	1	Case	SEARCH
Case_1704	1	Case	SEARCH
Case_1705	1	Case	SEARCH
PD30735a	1	Case	SEARCH
Case_1706	0.998950131	Case	SEARCH
Case_1707	1	Case	SEARCH
Case_1708	1	Case	SEARCH
Case_1709	0.999475066	Case	SEARCH
Case_1710	1	Case	SEARCH
Case_1711	0.998950131	Case	SEARCH
Case_1712	0.945406824	Case	SEARCH
Case_1713	1	Case	SEARCH
PD30704a	0.999475066	Case	SEARCH
Case_1714	1	Case	SEARCH
Case_1715	1	Case	SEARCH
Case_1716	0.999475066	Case	SEARCH

Case_1717	0.999475066	Case	SEARCH
Case_1718	1	Case	SEARCH
PD30706a	0.998950131	Case	SEARCH
Case_1719	1	Case	SEARCH
Case_1720	1	Case	SEARCH
Case_1721	1	Case	SEARCH
PD30707a	1	Case	SEARCH
Case_1722	1	Case	SEARCH
Case_1723	1	Case	SEARCH
Case_1724	1	Case	SEARCH
Case_1725	1	Case	SEARCH
Case_1726	0.997900262	Case	SEARCH
Case_1727	1	Case	SEARCH
Case_1728	1	Case	SEARCH
Case_1729	0.940682415	Case	SEARCH
Case_1730	1	Case	SEARCH
Case_1731	0.999475066	Case	SEARCH
Case_1732	1	Case	SEARCH
Case_1733	0.940682415	Case	SEARCH
Case_1734	1	Case	SEARCH
Case_1735	1	Case	SEARCH
Case_1736	1	Case	SEARCH
Case_1737	0.997375328	Case	SEARCH
Case_1738	0.998950131	Case	SEARCH
Case_1739	1	Case	SEARCH
Case_1740	1	Case	SEARCH
Case_1741	1	Case	SEARCH
Case_1742	1	Case	SEARCH
Case_1743	1	Case	SEARCH
Case_1744	1	Case	SEARCH
Case_1745	1	Case	SEARCH
Case_1746	1	Case	SEARCH
Case_1747	1	Case	SEARCH
Case_1748	0.998950131	Case	SEARCH
Case_1749	1	Case	SEARCH
Case_1750	1	Case	SEARCH
Case_1751	1	Case	SEARCH
Case_1752	1	Case	SEARCH
Case_1753	0.998950131	Case	SEARCH
Case_1754	1	Case	SEARCH

Case_1755	1	Case	SEARCH
Case_1756	1	Case	SEARCH
Case_1757	1	Case	SEARCH
Case_1758	1	Case	SEARCH
Case_1759	0.963779528	Case	SEARCH
Case_1760	1	Case	SEARCH
Case_1761	1	Case	SEARCH
Case_1762	1	Case	SEARCH
Case_1763	1	Case	SEARCH
Case_1764	1	Case	SEARCH
Case_1765	1	Case	SEARCH
Case_1766	1	Case	SEARCH
Case_1767	0.997900262	Case	SEARCH
Case_1768	0.940682415	Case	SEARCH
Case_1769	1	Case	SEARCH
Case_1770	1	Case	SEARCH
Case_1771	1	Case	SEARCH
Case_1772	1	Case	SEARCH
Case_1773	1	Case	SEARCH
Case_1774	0.998950131	Case	SEARCH
Case_1775	1	Case	SEARCH
Case_1776	1	Case	SEARCH
Case_1777	1	Case	SEARCH
Case_1778	1	Case	SEARCH
Case_1779	1	Case	SEARCH
Case_1780	1	Case	SEARCH
Case_1781	1	Case	SEARCH
Case_1782	1	Case	SEARCH
Case_1783	1	Case	SEARCH
Case_1784	0.998950131	Case	SEARCH
Case_1785	1	Case	SEARCH
Case_1786	1	Case	SEARCH
Case_1787	1	Case	SEARCH
Case_1788	0.963254593	Case	SEARCH
Case_1789	0.940682415	Case	SEARCH
Case_1790	0.998950131	Case	SEARCH
Case_1791	1	Case	SEARCH
Case_1792	1	Case	SEARCH
Case_1793	1	Case	SEARCH
Case_1794	1	Case	SEARCH

Case_1795	1	Case	SEARCH
Case_1796	1	Case	SEARCH
Case_1797	1	Case	SEARCH
Case_1798	0.997375328	Case	SEARCH
Case_1799	1	Case	SEARCH
Case_1800	0.999475066	Case	SEARCH
Case_1801	1	Case	SEARCH
Case_1802	1	Case	SEARCH
Case_1803	0.977952756	Case	SEARCH
Case_1804	1	Case	SEARCH
Case_1805	0.998950131	Case	SEARCH
Case_1806	1	Case	SEARCH
Case_1807	0.995800525	Case	SEARCH
Case_1808	0.998950131	Case	SEARCH
Case_1809	0.998425197	Case	SEARCH
Case_1810	1	Case	SEARCH
Case_1811	1	Case	SEARCH
Case_1812	1	Case	SEARCH
Case_1813	1	Case	SEARCH
Case_1814	0.999475066	Case	SEARCH
Case_1815	1	Case	SEARCH
PD30718a	1	Case	SEARCH
Case_1816	1	Case	SEARCH
Case_1817	1	Case	SEARCH
Case_1818	1	Case	SEARCH
Case_1819	1	Case	SEARCH
Case_1820	1	Case	SEARCH
Case_1821	1	Case	SEARCH
Case_1822	1	Case	SEARCH
Case_1823	1	Case	SEARCH
Case_1824	1	Case	SEARCH
Case_1825	1	Case	SEARCH
Case_1826	1	Case	SEARCH
Case_1827	1	Case	SEARCH
Case_1828	1	Case	SEARCH
Case_1829	1	Case	SEARCH
Case_1830	1	Case	SEARCH
Case_1831	1	Case	SEARCH
Case_1832	1	Case	SEARCH
Case_1833	1	Case	SEARCH

Case_1834	1	Case	SEARCH
Case_1835	1	Case	SEARCH
Case_1836	1	Case	SEARCH
Case_1837	1	Case	SEARCH
Case_1838	1	Case	SEARCH
Case_1839	0.998950131	Case	SEARCH
Case_1840	1	Case	SEARCH
Case_1841	1	Case	SEARCH
Case_1842	1	Case	SEARCH
Case_1843	1	Case	SEARCH
Case_1844	1	Case	SEARCH
Case_1845	1	Case	SEARCH
Case_1846	1	Case	SEARCH
Case_1847	1	Case	SEARCH
Case_1848	1	Case	SEARCH
Case_1849	1	Case	SEARCH
Case_1850	1	Case	SEARCH
Case_1851	1	Case	SEARCH
Case_1852	1	Case	SEARCH
Case_1853	1	Case	SEARCH
Case_1854	1	Case	SEARCH
Case_1855	1	Case	SEARCH
Case_1856	1	Case	SEARCH
Case_1857	0.998425197	Case	SEARCH
Case_1858	1	Case	SEARCH
PD30709a	1	Case	SEARCH
Case_1859	1	Case	SEARCH
Case_1860	1	Case	SEARCH
Case_1861	1	Case	SEARCH
Case_1862	1	Case	SEARCH
Case_1863	1	Case	SEARCH
Case_1864	1	Case	SEARCH
Case_1865	1	Case	SEARCH
Case_1866	1	Case	SEARCH
Case_1867	0.974278215	Case	SEARCH
Case_1868	1	Case	SEARCH
Case_1869	0.940682415	Case	SEARCH
Case_1870	1	Case	SEARCH
Case_1871	1	Case	SEARCH
Case_1872	0.999475066	Case	SEARCH

Case_1873	1	Case	SEARCH
Case_1874	1	Case	SEARCH
Case_1875	1	Case	SEARCH
Case_1876	1	Case	SEARCH
Case_1877	1	Case	SEARCH
Case_1878	1	Case	SEARCH
Case_1879	1	Case	SEARCH
Case_1880	1	Case	SEARCH
Case_1881	1	Case	SEARCH
Case_1882	1	Case	SEARCH
Case_1883	1	Case	SEARCH
Case_1884	0.940682415	Case	SEARCH
Case_1885	0.980577428	Case	SEARCH
Case_1886	1	Case	SEARCH
Case_1887	1	Case	SEARCH
Case_1888	1	Case	SEARCH
Case_1889	1	Case	SEARCH
Case_1890	1	Case	SEARCH
Case_1891	1	Case	SEARCH
Case_1892	1	Case	SEARCH
Case_1893	1	Case	SEARCH
Case_1894	1	Case	SEARCH
Case_1895	1	Case	SEARCH
Case_1896	0.999475066	Case	SEARCH
Case_1897	1	Case	SEARCH
Case_1898	1	Case	SEARCH
Case_1899	1	Case	SEARCH
Case_1900	1	Case	SEARCH
Case_1901	1	Case	SEARCH
Case_1902	0.999475066	Case	SEARCH
Case_1903	1	Case	SEARCH
Case_1904	1	Case	SEARCH
Case_1905	1	Case	SEARCH
Case_1906	1	Case	SEARCH
Case_1907	1	Case	SEARCH
Case_1908	1	Case	SEARCH
Case_1909	1	Case	SEARCH
Case_1910	1	Case	SEARCH
Case_1911	0.997375328	Case	SEARCH
Case_1912	1	Case	SEARCH

Case_1913	1	Case	SEARCH
Case_1914	1	Case	SEARCH
Case_1915	1	Case	SEARCH
Case_1916	1	Case	SEARCH
Case_1917	0.976377953	Case	SEARCH
PD30731a	1	Case	SEARCH
Case_1918	1	Case	SEARCH
Case_1919	1	Case	SEARCH
Case_1920	0.998950131	Case	SEARCH
Case_1921	1	Case	SEARCH
Case_1922	1	Case	SEARCH
Case_1923	0.947506562	Case	SEARCH
Case_1924	1	Case	SEARCH
Case_1925	1	Case	SEARCH
Case_1926	1	Case	SEARCH
Case_1927	1	Case	SEARCH
Case_1928	0.998425197	Case	SEARCH
Case_1929	0.998950131	Case	SEARCH
Case_1930	1	Case	SEARCH
Case_1931	0.999475066	Case	SEARCH
Case_1932	1	Case	SEARCH
Case_1933	1	Case	SEARCH
Case_1934	1	Case	SEARCH
Case_1935	1	Case	SEARCH
Case_1936	1	Case	SEARCH
Case_1937	0.999475066	Case	SEARCH
Case_1938	1	Case	SEARCH
Case_1939	1	Case	SEARCH
Case_1940	0.998425197	Case	SEARCH
Case_1941	1	Case	SEARCH
Case_1942	1	Case	SEARCH
Case_1943	1	Case	SEARCH
Case_1944	1	Case	SEARCH
Case_1945	1	Case	SEARCH
Case_1946	0.999475066	Case	SEARCH
Case_1947	0.990026247	Case	SEARCH
Case_1948	1	Case	SEARCH
Case_1949	1	Case	SEARCH
Case_1950	0.99160105	Case	SEARCH
Case_1951	1	Case	SEARCH

Case_1952	1	Case	SEARCH
Case_1953	1	Case	SEARCH
Case_1954	1	Case	SEARCH
Case_1955	1	Case	SEARCH
Case_1956	1	Case	SEARCH
Case_1957	1	Case	SEARCH
Case_1958	1	Case	SEARCH
Case_1959	0.97480315	Case	SEARCH
Case_1960	1	Case	SEARCH
Case_1961	1	Case	SEARCH
Case_1962	1	Case	SEARCH
Case_1963	1	Case	SEARCH
Case_1964	1	Case	SEARCH
Case_1965	1	Case	SEARCH
Case_1966	1	Case	SEARCH
Case_1967	0.999475066	Case	SEARCH
Case_1968	1	Case	SEARCH
Case_1969	1	Case	SEARCH
Case_1970	1	Case	SEARCH
Case_1971	1	Case	SEARCH
Case_1972	1	Case	SEARCH
Case_1973	1	Case	SEARCH
Case_1974	1	Case	SEARCH
Case_1975	1	Case	SEARCH
Case_1976	1	Case	SEARCH
Case_1977	1	Case	SEARCH
Case_1978	1	Case	SEARCH
Case_1979	1	Case	SEARCH
Case_1980	1	Case	SEARCH
Case_1981	1	Case	SEARCH
Case_1982	1	Case	SEARCH
Case_1983	1	Case	SEARCH
Case_1984	1	Case	SEARCH
Case_1985	1	Case	SEARCH
Case_1986	1	Case	SEARCH
Case_1987	1	Case	SEARCH
Case_1988	0.994750656	Case	SEARCH
Case_1989	1	Case	SEARCH
Case_1990	1	Case	SEARCH
Case_1991	1	Case	SEARCH

Case_1992	1	Case	SEARCH
Case_1993	1	Case	SEARCH
Case_1994	1	Case	SEARCH
Case_1995	1	Case	SEARCH
Case_1997	1	Case	SEARCH
Case_1998	1	Case	SEARCH
Case_1999	1	Case	SEARCH
Case_2000	1	Case	SEARCH
Case_2001	1	Case	SEARCH
Case_2002	1	Case	SEARCH
Case_2003	0.998950131	Case	SEARCH
Case_2004	0.968503937	Case	SEARCH
Case_2005	0.999475066	Case	SEARCH
Case_2006	1	Case	SEARCH
Case_2007	1	Case	SEARCH
Case_2008	1	Case	SEARCH
Case_2009	1	Case	SEARCH
Case_2010	1	Case	SEARCH
Case_2011	1	Case	SEARCH
Case_2012	0.951706037	Case	SEARCH
Case_2013	1	Case	SEARCH
Case_2014	1	Case	SEARCH
Case_2015	1	Case	SEARCH
Case_2016	1	Case	SEARCH
Case_2017	0.9832021	Case	SEARCH
Case_2018	1	Case	SEARCH
Case_2019	1	Case	SEARCH
Case_2020	0.999475066	Case	SEARCH
Case_2021	1	Case	SEARCH
Case_2022	1	Case	SEARCH
Case_2023	0.997375328	Case	SEARCH
Case_2024	1	Case	SEARCH
Case_2025	0.940682415	Case	SEARCH
Case_2026	0.993175853	Case	SEARCH
Case_2027	1	Case	SEARCH
Case_2028	1	Case	SEARCH
Case_2029	1	Case	SEARCH
Case_2030	1	Case	SEARCH
Case_2031	1	Case	SEARCH
Case_2032	1	Case	SEARCH

Case_2033	1	Case	SEARCH
Case_2034	1	Case	SEARCH
Case_2035	1	Case	SEARCH
Case_2036	1	Case	SEARCH
Case_2037	1	Case	SEARCH
Case_2038	0.999475066	Case	SEARCH
Case_2039	1	Case	SEARCH
Case_2040	1	Case	SEARCH
Case_2041	1	Case	SEARCH
Case_2042	1	Case	SEARCH
Case_2043	1	Case	SEARCH
Case_2044	1	Case	SEARCH
Case_2045	0.998950131	Case	SEARCH
Case_2046	1	Case	SEARCH
Case_2047	1	Case	SEARCH
Case_2048	1	Case	SEARCH
Case_2049	1	Case	SEARCH
Case_2050	1	Case	SEARCH
Case_2051	1	Case	SEARCH
Case_2052	1	Case	SEARCH
Case_2053	0.997375328	Case	SEARCH
Case_2054	1	Case	SEARCH
Case_2055	1	Case	SEARCH
Case_2056	1	Case	SEARCH
Case_2057	1	Case	SEARCH
Case_2058	1	Case	SEARCH
Case_2059	1	Case	SEARCH
Case_2060	1	Case	SEARCH
Case_2061	1	Case	SEARCH
Case_2062	1	Case	SEARCH
Case_2063	0.940682415	Case	SEARCH
Case_2064	1	Case	SEARCH
Case_2065	1	Case	SEARCH
PD30719a	1	Case	SEARCH
Case_2066	1	Case	SEARCH
Case_2067	1	Case	SEARCH
Case_2068	1	Case	SEARCH
Case_2069	1	Case	SEARCH
Case_2070	0.998425197	Case	SEARCH
Case_2071	1	Case	SEARCH

Case_2072	1	Case	SEARCH
Case_2073	1	Case	SEARCH
Case_2074	1	Case	SEARCH
Case_2075	1	Case	SEARCH
Case_2076	1	Case	SEARCH
Case_2077	1	Case	SEARCH
Case_2078	1	Case	SEARCH
Case_2079	1	Case	SEARCH
Case_2080	1	Case	SEARCH
Case_2081	1	Case	SEARCH
Case_2082	1	Case	SEARCH
Case_2083	1	Case	SEARCH
Case_2084	1	Case	SEARCH
Case_2085	1	Case	SEARCH
Case_2086	1	Case	SEARCH
Case_2087	1	Case	SEARCH
Case_2088	1	Case	SEARCH
Case_2089	1	Case	SEARCH
Case_2090	1	Case	SEARCH
Case_2091	1	Case	SEARCH
Case_2092	1	Case	SEARCH
Case_2093	1	Case	SEARCH
Case_2094	0.997900262	Case	SEARCH
Case_2095	0.991076115	Case	SEARCH
Case_2096	1	Case	SEARCH
Case_2097	0.999475066	Case	SEARCH
Case_2098	1	Case	SEARCH
Case_2099	1	Case	SEARCH
Case_2100	1	Case	SEARCH
Case_2101	1	Case	SEARCH
Case_2102	1	Case	SEARCH
Case_2103	0.998950131	Case	SEARCH
Case_2104	1	Case	SEARCH
Case_2105	1	Case	SEARCH
Case_2106	1	Case	SEARCH
Case_2107	1	Case	SEARCH
Case_2108	1	Case	SEARCH
Case_2109	1	Case	SEARCH
Case_2110	1	Case	SEARCH
Case_2111	1	Case	SEARCH

Case_2112	1	Case	SEARCH
Case_2113	0.999475066	Case	SEARCH
Case_2114	1	Case	SEARCH
PD30720a	1	Case	SEARCH
Case_2115	1	Case	SEARCH
Case_2116	1	Case	SEARCH
Case_2117	1	Case	SEARCH
Case_2118	1	Case	SEARCH
Case_2119	0.997375328	Case	SEARCH
Case_2120	0.999475066	Case	SEARCH
Case_2121	1	Case	SEARCH
Case_2122	1	Case	SEARCH
Case_2123	1	Case	SEARCH
Case_2124	0.996325459	Case	SEARCH
Case_2125	1	Case	SEARCH
Case_2126	1	Case	SEARCH
Case_2127	1	Case	SEARCH
Case_2128	1	Case	SEARCH
Case_2129	0.997900262	Case	SEARCH
Case_2130	0.940682415	Case	SEARCH
Case_2131	1	Case	SEARCH
Case_2132	1	Case	SEARCH
Case_2133	0.999475066	Case	SEARCH
Case_2134	1	Case	SEARCH
Case_2135	1	Case	SEARCH
Case_2136	1	Case	SEARCH
Case_2137	1	Case	SEARCH
Case_2138	1	Case	SEARCH
Case_2139	0.986351706	Case	SEARCH
Case_2140	0.993700787	Case	SEARCH
Case_2141	1	Case	SEARCH
PD30708a	1	Case	SEARCH
Case_2142	0.999475066	Case	SEARCH
Case_2143	1	Case	SEARCH
Case_2144	1	Case	SEARCH
PD30732a	1	Case	SEARCH
Case_2145	0.999475066	Case	SEARCH
Case_2146	1	Case	SEARCH
Case_2147	1	Case	SEARCH
Case_2148	1	Case	SEARCH

Case_2149	1	Case	SEARCH
Case_2150	0.999475066	Case	SEARCH
Case_2151	1	Case	SEARCH
Case_2152	1	Case	SEARCH
Case_2153	1	Case	SEARCH
Case_2154	1	Case	SEARCH
Case_2155	1	Case	SEARCH
Case_2156	1	Case	SEARCH
Case_2157	0.999475066	Case	SEARCH
Case_2158	1	Case	SEARCH
Case_2159	1	Case	SEARCH
PD30705a	0.997900262	Case	SEARCH
Case_2160	1	Case	SEARCH
Case_2161	1	Case	SEARCH
Case_2162	1	Case	SEARCH
Case_2163	1	Case	SEARCH
Case_2164	1	Case	SEARCH
Case_2165	1	Case	SEARCH
Case_2166	1	Case	SEARCH
Case_2167	1	Case	SEARCH
Case_2168	1	Case	SEARCH
Case_2169	1	Case	SEARCH
Case_2170	0.999475066	Case	SEARCH
Case_2171	1	Case	SEARCH
Case_2172	1	Case	SEARCH
Case_2173	1	Case	SEARCH
Case_2174	1	Case	SEARCH
Case_2175	1	Case	SEARCH
Case_2176	1	Case	SEARCH
Case_2177	1	Case	SEARCH
Case_2178	1	Case	SEARCH
Case_2179	0.998950131	Case	SEARCH
Case_2180	1	Case	SEARCH
Case_2181	1	Case	SEARCH
Case_2182	1	Case	SEARCH
Case_2183	0.94015748	Case	SEARCH
Case_2184	1	Case	SEARCH
Case_2185	1	Case	SEARCH
Case_2186	1	Case	SEARCH
Case_2187	1	Case	SEARCH

Case_2188	0.940682415	Case	SEARCH
Case_2189	1	Case	SEARCH
Case_2190	1	Case	SEARCH
Case_2191	0.940682415	Case	SEARCH
Case_2192	1	Case	SEARCH
Case_2193	1	Case	SEARCH
Case_2194	0.940682415	Case	SEARCH
Case_2195	1	Case	SEARCH
Case_2196	1	Case	SEARCH
Case_2197	1	Case	SEARCH
Case_2198	1	Case	SEARCH
Case_2199	1	Case	SEARCH
PD30733a	1	Case	SEARCH
Case_2200	0.940682415	Case	SEARCH
Case_2201	1	Case	SEARCH
Case_2202	1	Case	SEARCH
Case_2203	1	Case	SEARCH
Case_2204	1	Case	SEARCH
Case_2205	1	Case	SEARCH
Case_2206	1	Case	SEARCH
Case_2207	1	Case	SEARCH
Case_2208	0.997900262	Case	SEARCH
Case_2209	1	Case	SEARCH
Case_2210	1	Case	SEARCH
Case_2211	0.999475066	Case	SEARCH
Case_2212	1	Case	SEARCH
Case_2213	1	Case	SEARCH
Case_2214	1	Case	SEARCH
Case_2215	1	Case	SEARCH
Case_2216	1	Case	SEARCH
Case_2217	1	Case	SEARCH
Case_2218	1	Case	SEARCH
Case_2219	1	Case	SEARCH
Case_2220	1	Case	SEARCH
Case_2221	1	Case	SEARCH
Case_2222	1	Case	SEARCH
Case_2223	1	Case	SEARCH
Case_2224	1	Case	SEARCH
Case_2225	1	Case	SEARCH
Case_2226	1	Case	SEARCH

Case_2227	1	Case	SEARCH
Case_2228	1	Case	SEARCH
Case_2229	1	Case	SEARCH
Case_2230	0.999475066	Case	SEARCH
Case_2231	0.984251969	Case	SEARCH
Case_2232	1	Case	SEARCH
Case_2233	1	Case	SEARCH
Case_2234	1	Case	SEARCH
Case_2235	1	Case	SEARCH
Case_2236	0.998425197	Case	SEARCH
Case_2237	1	Case	SEARCH
Case_2238	1	Case	SEARCH
Case_2239	1	Case	SEARCH
Case_2240	1	Case	SEARCH
Case_2241	1	Case	SEARCH
Case_2242	1	Case	SEARCH
Case_2243	1	Case	SEARCH
Case_2244	1	Case	SEARCH
Case_2245	1	Case	SEARCH
Case_2246	1	Case	SEARCH
Case_2247	1	Case	SEARCH
Case_2248	1	Case	SEARCH
PD30712a	1	Case	SEARCH
Case_2249	0.974278215	Case	SEARCH
Case_2250	1	Case	SEARCH
Case_2251	0.940682415	Case	SEARCH
Case_2252	1	Case	SEARCH
Case_2253	1	Case	SEARCH
Case_2254	1	Case	SEARCH
Case_2255	1	Case	SEARCH
Case_2256	1	Case	SEARCH
PD30713a	1	Case	SEARCH
Case_2257	0.998950131	Case	SEARCH
Case_2258	1	Case	SEARCH
Case_2259	0.997900262	Case	SEARCH
Case_2260	0.940682415	Case	SEARCH
Case_2261	1	Case	SEARCH
Case_2262	1	Case	SEARCH
Case_2263	1	Case	SEARCH
Case_2264	1	Case	SEARCH

Case_2265	1	Case	SEARCH
Case_2266	1	Case	SEARCH
Case_2267	1	Case	SEARCH
Case_2268	1	Case	SEARCH
Case_2269	1	Case	SEARCH
Case_2270	1	Case	SEARCH
Case_2271	1	Case	SEARCH
Case_2272	0.998425197	Case	SEARCH
Case_2273	1	Case	SEARCH
Case_2274	1	Case	SEARCH
Case_2275	1	Case	SEARCH
Case_2276	1	Case	SEARCH
Case_2277	1	Case	SEARCH
Case_2278	1	Case	SEARCH
Case_2279	1	Case	SEARCH
Case_2280	0.954330709	Case	SEARCH
PD30714a	1	Case	SEARCH
Case_2281	1	Case	SEARCH
Case_2282	1	Case	SEARCH
Case_2283	1	Case	SEARCH
Case_2284	1	Case	SEARCH
Case_2285	1	Case	SEARCH
Case_2286	1	Case	SEARCH
Case_2287	0.999475066	Case	SEARCH
Case_2288	1	Case	SEARCH
Case_2289	1	Case	SEARCH
Case_2290	1	Case	SEARCH
Case_2291	0.997375328	Case	SEARCH
Case_2292	1	Case	SEARCH
Case_2293	0.999475066	Case	SEARCH
Case_2294	1	Case	SEARCH
Case_2295	1	Case	SEARCH
Case_2296	1	Case	SEARCH
Case_2297	1	Case	SEARCH
Case_2298	1	Case	SEARCH
Case_2299	1	Case	SEARCH
Case_2300	1	Case	SEARCH
Case_2301	1	Case	SEARCH
Case_2302	1	Case	SEARCH
Case_2303	1	Case	SEARCH

Case_2304	1	Case	SEARCH
Case_2305	1	Case	SEARCH
Case_2306	1	Case	SEARCH
Case_2307	1	Case	SEARCH
Case_2308	1	Case	SEARCH
Case_2309	1	Case	SEARCH
Case_2310	1	Case	SEARCH
Case_2311	1	Case	SEARCH
Case_2312	1	Case	SEARCH
Case_2313	1	Case	SEARCH
Case_2314	1	Case	SEARCH
Case_2315	1	Case	SEARCH
Case_2316	1	Case	SEARCH
Case_2317	0.999475066	Case	SEARCH
Case_2318	1	Case	SEARCH
PD30724a	0.954855643	Case	SEARCH
Case_2319	1	Case	SEARCH
Case_2320	1	Case	SEARCH
Case_2321	1	Case	SEARCH
Case_2322	0.998950131	Case	SEARCH
Case_2323	1	Case	SEARCH
Case_2324	1	Case	SEARCH
Case_2325	1	Case	SEARCH
Case_2326	1	Case	SEARCH
Case_2327	1	Case	SEARCH
Case_2328	1	Case	SEARCH
Case_2329	1	Case	SEARCH
Case_2330	1	Case	SEARCH
Case_2331	1	Case	SEARCH
Case_2332	1	Case	SEARCH
Case_2333	1	Case	SEARCH
Case_2334	1	Case	SEARCH
Case_2335	1	Case	SEARCH
Case_2336	1	Case	SEARCH
Case_2337	1	Case	SEARCH
Case_2338	1	Case	SEARCH
Case_2339	1	Case	SEARCH
Case_2340	1	Case	SEARCH
Case_2341	1	Case	SEARCH
Case_2342	1	Case	SEARCH

Case_2343	1	Case	SEARCH
Case_2344	1	Case	SEARCH
Case_2345	1	Case	SEARCH
Case_2346	1	Case	SEARCH
Case_2347	1	Case	SEARCH
Case_2348	1	Case	SEARCH
Case_2349	1	Case	SEARCH
Case_2350	1	Case	SEARCH
Case_2351	1	Case	SEARCH
Case_2352	1	Case	SEARCH
Case_2353	0.940682415	Case	SEARCH
Case_2354	1	Case	SEARCH
Case_2355	1	Case	SEARCH
Case_2356	1	Case	SEARCH
Case_2357	1	Case	SEARCH
Case_2358	0.985826772	Case	SEARCH
Case_2359	1	Case	SEARCH
Case_2360	1	Case	SEARCH
Case_2361	1	Case	SEARCH
Case_2362	1	Case	SEARCH
Case_2363	1	Case	SEARCH
Case_2364	1	Case	SEARCH
Case_2365	1	Case	SEARCH
Case_2366	1	Case	SEARCH
Case_2367	0.999475066	Case	SEARCH
Case_2368	1	Case	SEARCH
PD30721a	1	Case	SEARCH
Case_2369	1	Case	SEARCH
Case_2370	1	Case	SEARCH
Case_2371	1	Case	SEARCH
Case_2372	1	Case	SEARCH
Case_2373	1	Case	SEARCH
Case_2374	0.999475066	Case	SEARCH
Case_2375	0.985301837	Case	SEARCH
Case_2376	1	Case	SEARCH
Case_2377	1	Case	SEARCH
Case_2378	1	Case	SEARCH
Case_2379	1	Case	SEARCH
Case_2380	0.998950131	Case	SEARCH
Case_2381	1	Case	SEARCH

Case_2382	1	Case	SEARCH
Case_2383	0.940682415	Case	SEARCH
Case_2384	0.999475066	Case	SEARCH
Case_2385	1	Case	SEARCH
Case_2386	1	Case	SEARCH
Case_2387	0.999475066	Case	SEARCH
Case_2388	0.940682415	Case	SEARCH
Case_2389	1	Case	SEARCH
Case_2390	1	Case	SEARCH
Case_2391	1	Case	SEARCH
Case_2392	1	Case	SEARCH
Case_2393	1	Case	SEARCH
Case_2394	1	Case	SEARCH
Case_2395	1	Case	SEARCH
Case_2396	1	Case	SEARCH
Case_2397	1	Case	SEARCH
Case_2398	1	Case	SEARCH
Case_2399	1	Case	SEARCH
Case_2400	0.998950131	Case	SEARCH
Case_2401	1	Case	SEARCH
Case_2402	1	Case	SEARCH
Case_2403	0.993175853	Case	SEARCH
Case_2404	0.999475066	Case	SEARCH
Case_2405	1	Case	SEARCH
Case_2406	0.999475066	Case	SEARCH
Case_2407	1	Case	SEARCH
Case_2408	0.940682415	Case	SEARCH
Case_2409	1	Case	SEARCH
Case_2410	1	Case	SEARCH
Case_2411	1	Case	SEARCH
Case_2412	1	Case	SEARCH
Case_2413	1	Case	SEARCH
Case_2414	1	Case	SEARCH
Case_2415	1	Case	SEARCH
Case_2416	1	Case	SEARCH
Case_2417	1	Case	SEARCH
Case_2418	1	Case	SEARCH
Case_2419	1	Case	SEARCH
Case_2420	1	Case	SEARCH
Case_2421	0.998950131	Case	SEARCH

Case_2422	1	Case	SEARCH
Case_2423	1	Case	SEARCH
Case_2424	0.998950131	Case	SEARCH
Case_2425	1	Case	SEARCH
Case_2426	0.997375328	Case	SEARCH
Case_2427	1	Case	SEARCH
PD30700a	0.940682415	Case	SEARCH
Case_2428	1	Case	SEARCH
Case_2429	0.999475066	Case	SEARCH
Case_2430	1	Case	SEARCH
PD30722a	1	Case	SEARCH
Case_2431	1	Case	SEARCH
Case_2432	1	Case	SEARCH
Case_2433	1	Case	SEARCH
Case_2434	1	Case	SEARCH
Case_2435	1	Case	SEARCH
Case_2436	1	Case	SEARCH
Case_2437	1	Case	SEARCH
Case_2438	1	Case	SEARCH
Case_2439	1	Case	SEARCH
Case_2440	1	Case	SEARCH
Case_2441	1	Case	SEARCH
Case_2442	0.94488189	Case	SEARCH
Case_2443	1	Case	SEARCH
Case_2444	1	Case	SEARCH
Case_2445	1	Case	SEARCH
Case_2446	0.999475066	Case	SEARCH
Case_2447	0.999475066	Case	SEARCH
Case_2448	1	Case	SEARCH
Case_2449	1	Case	SEARCH
Case_2450	1	Case	SEARCH
Case_2451	1	Case	SEARCH
Case_2452	1	Case	SEARCH
Case_2453	1	Case	SEARCH
Case_2454	1	Case	SEARCH
Case_2455	1	Case	SEARCH
Case_2456	1	Case	SEARCH
Case_2457	1	Case	SEARCH
Case_2458	0.998425197	Case	SEARCH
Case_2459	1	Case	SEARCH

Case_2460	1	Case	SEARCH
Case_2461	0.996850394	Case	SEARCH
Case_2462	1	Case	SEARCH
Case_2463	1	Case	SEARCH
Case_2464	1	Case	SEARCH
Case_2465	1	Case	SEARCH
Case_2466	0.999475066	Case	SEARCH
Case_2467	1	Case	SEARCH
Case_2468	1	Case	SEARCH
Case_2469	1	Case	SEARCH
Case_2470	1	Case	SEARCH
PD30701a	0.999475066	Case	SEARCH
Case_2471	1	Case	SEARCH
Case_2472	1	Case	SEARCH
Case_2473	1	Case	SEARCH
Case_2474	1	Case	SEARCH
Case_2475	1	Case	SEARCH
Case_2476	1	Case	SEARCH
Case_2477	1	Case	SEARCH
Case_2478	1	Case	SEARCH
Case_2479	1	Case	SEARCH
Case_2480	0.999475066	Case	SEARCH
Case_2481	1	Case	SEARCH
Case_2482	1	Case	SEARCH
Case_2483	1	Case	SEARCH
Case_2484	1	Case	SEARCH
Case_2485	0.998425197	Case	SEARCH
Case_2486	1	Case	SEARCH
Case_2487	1	Case	SEARCH
Case_2488	1	Case	SEARCH
Case_2489	1	Case	SEARCH
Case_2490	0.996325459	Case	SEARCH
Case_2491	1	Case	SEARCH
Case_2492	1	Case	SEARCH
Case_2493	1	Case	SEARCH
Case_2494	1	Case	SEARCH
Case_2495	1	Case	SEARCH
Case_2496	1	Case	SEARCH
Case_2497	0.999475066	Case	SEARCH
Case_2498	1	Case	SEARCH

Case_2499	1	Case	SEARCH
Case_2500	1	Case	SEARCH
Case_2501	1	Case	SEARCH
Case_2502	1	Case	SEARCH
Case_2503	1	Case	SEARCH
Case_2504	1	Case	SEARCH
Case_2505	1	Case	SEARCH
Case_2506	1	Case	SEARCH
Case_2507	0.998950131	Case	SEARCH
Case_2508	1	Case	SEARCH
Case_2509	1	Case	SEARCH
Case_2510	1	Case	SEARCH
Case_2511	0.999475066	Case	SEARCH
Case_2512	1	Case	SEARCH
Case_2513	0.945931759	Case	SEARCH
Case_2514	1	Case	SEARCH
Case_2515	1	Case	SEARCH
Case_2516	1	Case	SEARCH
Case_2517	1	Case	SEARCH
Case_2518	1	Case	SEARCH
Case_2519	1	Case	SEARCH
Case_2520	1	Case	SEARCH
Case_2521	1	Case	SEARCH
Case_2522	1	Case	SEARCH
Case_2523	0.998950131	Case	SEARCH
Case_2524	1	Case	SEARCH
Case_2525	1	Case	SEARCH
Case_2526	1	Case	SEARCH
Case_2527	0.994225722	Case	SEARCH
Case_2528	1	Case	SEARCH
Case_2529	1	Case	SEARCH
Case_2530	1	Case	SEARCH
PD30725a	0.984776903	Case	SEARCH
Case_2531	1	Case	SEARCH
Case_2532	1	Case	SEARCH
Case_2533	1	Case	SEARCH
Case_2534	1	Case	SEARCH
Case_2535	1	Case	SEARCH
Case_2536	0.96167979	Case	SEARCH
Case_2537	1	Case	SEARCH

Case_2538	1	Case	SEARCH
Case_2539	0.998425197	Case	SEARCH
Case_2540	1	Case	SEARCH
Case_2541	1	Case	SEARCH
Case_2542	0.972703412	Case	SEARCH
Case_2543	1	Case	SEARCH
Case_2544	1	Case	SEARCH
Case_2545	1	Case	SEARCH
Case_2546	1	Case	SEARCH
Case_2547	1	Case	SEARCH
Case_2548	1	Case	SEARCH
PD30723a	1	Case	SEARCH
Case_2549	1	Case	SEARCH
Case_2550	1	Case	SEARCH
Case_2551	1	Case	SEARCH
Case_2552	1	Case	SEARCH
Case_2553	1	Case	SEARCH
Case_2554	1	Case	SEARCH
Case_2555	1	Case	SEARCH
Case_2556	1	Case	SEARCH
Case_2557	1	Case	SEARCH
Case_2558	1	Case	SEARCH
Case_2559	0.940682415	Case	SEARCH
Case_2560	0.999475066	Case	SEARCH
Case_2561	1	Case	SEARCH
Case_2562	1	Case	SEARCH
Case_2563	1	Case	SEARCH
Case_2564	1	Case	SEARCH
Case_2565	0.998425197	Case	SEARCH
Case_2566	1	Case	SEARCH
Case_2567	1	Case	SEARCH
Case_2568	1	Case	SEARCH
Case_2569	1	Case	SEARCH
Case_2570	0.996325459	Case	SEARCH
Case_2571	1	Case	SEARCH
Case_2572	1	Case	SEARCH
Case_2573	1	Case	SEARCH
Case_2574	1	Case	SEARCH
Case_2575	1	Case	SEARCH
Case_2576	1	Case	SEARCH

Case_2577	1	Case	SEARCH
Case_2578	1	Case	SEARCH
Case_2579	1	Case	SEARCH
Case_2580	1	Case	SEARCH
Case_2581	1	Case	SEARCH
Case_2582	1	Case	SEARCH
Case_2583	1	Case	SEARCH
Case_2584	0.999475066	Case	SEARCH
Case_2585	1	Case	SEARCH
Case_2586	1	Case	SEARCH
Case_2587	0.998950131	Case	SEARCH
Case_2588	1	Case	SEARCH
Case_2589	1	Case	SEARCH
Case_2590	1	Case	SEARCH
Case_2591	1	Case	SEARCH
Case_2592	1	Case	SEARCH
Case_2593	1	Case	SEARCH
Case_2594	1	Case	SEARCH
Case_2595	1	Case	SEARCH
Case_2596	1	Case	SEARCH
Case_2597	1	Case	SEARCH
Case_2598	1	Case	SEARCH
Case_2599	1	Case	SEARCH
Case_2600	1	Case	SEARCH
Case_2601	1	Case	SEARCH
Case_2602	1	Case	SEARCH
Case_2603	1	Case	SEARCH
Case_2604	1	Case	SEARCH
Case_2605	1	Case	SEARCH
Case_2606	1	Case	SEARCH
Case_2607	1	Case	SEARCH
PD30736a	1	Case	SEARCH
Case_2608	1	Case	SEARCH
Case_2609	1	Case	SEARCH
Case_2610	1	Case	SEARCH
Case_2611	1	Case	SEARCH
Case_2612	1	Case	SEARCH
Case_2613	1	Case	SEARCH
Case_2614	1	Case	SEARCH
Case_2615	1	Case	SEARCH

Case_2616	1	Case	SEARCH
Case_2617	1	Case	SEARCH
Case_2618	0.998950131	Case	SEARCH
Case_2619	1	Case	SEARCH
Case_2620	0.998950131	Case	SEARCH
Case_2621	1	Case	SEARCH
Case_2622	0.998950131	Case	SEARCH
Case_2623	0.986351706	Case	SEARCH
Case_2624	0.999475066	Case	SEARCH
Case_2625	1	Case	SEARCH
Case_2626	1	Case	SEARCH
Case_2627	1	Case	SEARCH
Case_2628	1	Case	SEARCH
Case_2629	1	Case	SEARCH
Case_2630	1	Case	SEARCH
Case_2631	1	Case	SEARCH
Case_2632	1	Case	SEARCH
Case_2633	0.999475066	Case	SEARCH
Case_2634	1	Case	SEARCH
Case_2635	1	Case	SEARCH
Case_2636	1	Case	SEARCH
Case_2637	1	Case	SEARCH
Case_2638	1	Case	SEARCH
Case_2639	1	Case	SEARCH
Case_2640	1	Case	SEARCH
Case_2641	1	Case	SEARCH
Case_2642	1	Case	SEARCH
PD30726a	0.998425197	Case	SEARCH
Case_2643	1	Case	SEARCH
Case_2644	1	Case	SEARCH
Case_2645	1	Case	SEARCH
Case_2646	1	Case	SEARCH
Case_2647	1	Case	SEARCH
Case_2648	1	Case	SEARCH
Case_2649	1	Case	SEARCH
Case_2650	0.940682415	Case	SEARCH
Case_2651	1	Case	SEARCH
Case_2652	1	Case	SEARCH
Case_2653	0.999475066	Case	SEARCH
Case_2654	1	Case	SEARCH

Case_2655	1	Case	SEARCH
Case_2656	1	Case	SEARCH
Case_2657	1	Case	SEARCH
Case_2658	0.998950131	Case	SEARCH
Case_2659	0.998950131	Case	SEARCH
Case_2660	1	Case	SEARCH
Case_2661	0.984251969	Case	SEARCH
Case_2662	1	Case	SEARCH
Case_2663	0.999475066	Case	SEARCH
Case_2664	1	Case	SEARCH
Case_2665	1	Case	SEARCH
Case_2666	1	Case	SEARCH
Case_2667	1	Case	SEARCH
Case_2668	1	Case	SEARCH
Case_2669	1	Case	SEARCH
Case_2670	1	Case	SEARCH
Case_2671	1	Case	SEARCH
Case_2672	1	Case	SEARCH
Case_2673	0.999475066	Case	SEARCH
Case_2674	1	Case	SEARCH
Case_2675	1	Case	SEARCH
Case_2676	1	Case	SEARCH
Case_2677	1	Case	SEARCH
Case_2678	1	Case	SEARCH
Case_2679	1	Case	SEARCH
PD30710a	1	Case	SEARCH
Case_2680	1	Case	SEARCH
Case_2681	1	Case	SEARCH
Case_2682	1	Case	SEARCH
Case_2683	1	Case	SEARCH
Case_2684	1	Case	SEARCH
Case_2685	1	Case	SEARCH
Case_2686	1	Case	SEARCH
Case_2687	1	Case	SEARCH
Case_2688	1	Case	SEARCH
Case_2689	1	Case	SEARCH
Case_2690	1	Case	SEARCH
Case_2691	0.998425197	Case	SEARCH
Case_2692	1	Case	SEARCH
PD30695a	1	Case	SEARCH

Case_2693	1	Case	SEARCH
Case_2694	1	Case	SEARCH
Case_2695	0.999475066	Case	SEARCH
Case_2696	1	Case	SEARCH
Case_2697	1	Case	SEARCH
Case_2698	1	Case	SEARCH
Case_2699	1	Case	SEARCH
Case_2700	0.998950131	Case	SEARCH
Case_2701	1	Case	SEARCH
Case_2702	0.996325459	Case	SEARCH
Case_2703	1	Case	SEARCH
Case_2704	1	Case	SEARCH
Case_2705	1	Case	SEARCH
Case_2706	1	Case	SEARCH
Case_2707	1	Case	SEARCH
Case_2708	1	Case	SEARCH
Case_2709	1	Case	SEARCH
Case_2710	1	Case	SEARCH
Case_2711	1	Case	SEARCH
Case_2712	1	Case	SEARCH
Case_2713	1	Case	SEARCH
Case_2714	1	Case	SEARCH
Case_2715	0.999475066	Case	SEARCH
Case_2716	0.998950131	Case	SEARCH
Case_2717	0.981627297	Case	SEARCH
Case_2718	1	Case	SEARCH
Case_2719	1	Case	SEARCH
Case_2720	1	Case	SEARCH
Case_2721	1	Case	SEARCH
Case_2722	1	Case	SEARCH
Case_2723	1	Case	SEARCH
Case_2724	1	Case	SEARCH
Case_2725	1	Case	SEARCH
Case_2726	1	Case	SEARCH
Case_2727	1	Case	SEARCH
Case_2728	1	Case	SEARCH
Case_2729	1	Case	SEARCH
Case_2730	1	Case	SEARCH
Case_2731	1	Case	SEARCH
Case_2732	1	Case	SEARCH

Case_2733	1	Case	SEARCH
Case_2734	1	Case	SEARCH
Case_2735	0.993700787	Case	SEARCH
Case_2736	1	Case	SEARCH
Case_2737	1	Case	SEARCH
Case_2738	1	Case	SEARCH
Case_2739	1	Case	SEARCH
Case_2740	1	Case	SEARCH
Case_2741	0.997900262	Case	SEARCH
Case_2742	1	Case	SEARCH
Case_2743	1	Case	SEARCH
Case_2744	1	Case	SEARCH
Case_2745	1	Case	SEARCH
Case_2746	1	Case	SEARCH
Case_2747	1	Case	SEARCH
Case_2748	0.998950131	Case	SEARCH
Case_2749	1	Case	SEARCH
PD30734a	1	Case	SEARCH
Case_2750	1	Case	SEARCH
Case_2751	1	Case	SEARCH
Case_2752	1	Case	SEARCH
Case_2753	1	Case	SEARCH
Case_2754	1	Case	SEARCH
Case_2755	1	Case	SEARCH
Case_2756	1	Case	SEARCH
Case_2757	1	Case	SEARCH
Case_2758	1	Case	SEARCH
Case_2759	1	Case	SEARCH
Case_2760	1	Case	SEARCH
Case_2761	1	Case	SEARCH
Case_2762	1	Case	SEARCH
Case_2763	1	Case	SEARCH
Case_2764	1	Case	SEARCH
Case_2765	1	Case	SEARCH
Case_2766	1	Case	SEARCH
Case_2767	0.962729659	Case	SEARCH
Case_2768	0.999475066	Case	SEARCH
Case_2769	0.999475066	Case	SEARCH
Case_2770	1	Case	SEARCH
Case_2771	1	Case	SEARCH

Case_2772	1	Case	SEARCH
Case_2773	1	Case	SEARCH
Case_2774	1	Case	SEARCH
Case_2775	1	Case	SEARCH
Case_2776	1	Case	SEARCH
Case_2777	1	Case	SEARCH
Case_2778	1	Case	SEARCH
Case_2779	0.98687664	Case	SEARCH
Case_2780	1	Case	SEARCH
Case_2781	1	Case	SEARCH
PD30715a	1	Case	SEARCH
Case_2782	1	Case	SEARCH
Case_2783	1	Case	SEARCH
Case_2784	1	Case	SEARCH
Case_2785	1	Case	SEARCH
Case_2786	1	Case	SEARCH
Case_2787	1	Case	SEARCH
Case_2788	0.994225722	Case	SEARCH
Case_2789	1	Case	SEARCH
PD30730a	1	Case	SEARCH
Case_2790	0.981102362	Case	SEARCH
Case_2791	1	Case	SEARCH
Case_2792	1	Case	SEARCH
Case_2793	1	Case	SEARCH
Case_2794	0.999475066	Case	SEARCH
Case_2795	1	Case	SEARCH
Case_2796	0.999475066	Case	SEARCH
Case_2797	1	Case	SEARCH
Case_2798	1	Case	SEARCH
Case_2799	1	Case	SEARCH
Case_2800	1	Case	SEARCH
Case_2801	1	Case	SEARCH
Case_2802	1	Case	SEARCH

Supplementary Table 15. List of samples that were re-sequenced by Illumina for confirmation. The average coverage of coding *POT1* exons is included. In red, samples with a coverage lower than 10.

Sample	Average coverage
PD30545a	287.8183206
PD30546a	288.3867684
PD30547a	316.0020356
PD30548a	261.8880407
PD30549a	270.092112
PD30550a	276.5094148
PD30551a	233.7791349
PD30552a	280.6137405
PD30553a	213.3725191
PD30554a	222.3903308
PD30555a	251.7562341
PD30556a	274.5073791
PD30557a	232.5338422
PD30558a	258.5124682
PD30559a	238.7760814
PD30560a	272.6096692
PD30561a	222.6605598
PD30562a	219.8117048
PD30563a	199.8819338
PD30564a	205.2101781
PD30565a	207.2946565
PD30566a	162.3760814
PD30567a	179.4575064
PD30568a	242.4076336
PD30569a	221.9236641
PD30570a	192.2992366
PD30571a	203.7043257
PD30572a	200.3638677
PD30573a	240.3857506
PD30574a	176.0834606
PD30575a	171.8956743
PD30576a	283.5114504

PD30577a	39.16132316
PD30578a	197.389313
PD30580a	210.7857506
PD30581a	198.3964377
PD30583a	46.1821883
PD30584a	297.8717557
PD30585a	249.1292621
PD30586a	226.3501272
PD30587a	193.0188295
PD30588a	202.0259542
PD30589a	172.4081425
PD30590a	209.4025445
PD30591a	217.8610687
PD30592a	151.3547074
PD30593a	258.092112
PD30594a	254.4625954
PD30595a	277.8605598
PD30596a	370.070229
PD30597a	255.5842239
PD30598a	282.1699746
PD30599a	621.8208651
PD30600a	327.9231552
PD30602a	388.0508906
PD30603a	353.3725191
PD30605a	138.205598
PD30606a	254.4748092
PD30607a	95.09363868
PD30609a	194.2732824
PD30611a	15.45343511
PD30612a	35.04529262
PD30616a	21.76743003
PD30619a	17.80050891
PD30621a	9.415776081
PD30625a	50.44732824
PD30627a	113.9089059
PD30628a	21.96234097
PD30629a	3.165903308
PD30630a	45.34249364
PD30632a	36.01475827

PD30633a	15.52875318
PD30634a	9.458015267
PD30635a	32.99592875
PD30636a	26.81017812
PD30637a	15.63206107
PD30638a	15.51094148
PD30639a	9.541984733
PD30642a	59.01577608
PD30643a	60.43613232
PD30644a	44.78015267
PD30646a	74.86310433
PD30647a	16.94045802
PD30648a	38.43358779
PD30650a	33.04478372
PD30651a	26.5129771
PD30652a	36.93180662
PD30654a	79.25292621
PD30655a	11.03918575
PD30656a	4.024936387
PD30657a	17.70585242
PD30659a	14.7216285
PD30660a	12.85496183
PD30661a	20.13231552
PD30662a	194.1725191
PD30663a	27.2783715
PD30664a	220.9577608
PD30665a	197.2371501
PD30668a	16.32315522
PD30682a	15.10025445
PD30683a	72.46412214
PD30684a	7.121119593
PD30689a	10.51501272
PD30693a	42.9740458
PD30695a	194.9338422
PD30697a	83.20101781
PD30698a	73.72315522
PD30699a	179.6483461
PD30700a	206.3348601
PD30701a	139.0452926

PD30702a	170.1180662
PD30705a	167.4447837
PD30707a	167.2417303
PD30708a	124.3145038
PD30710a	156.3195929
PD30711a	18.57201018
PD30712a	180.1597964
PD30713a	206.6977099
PD30714a	200.1389313
PD30715a	250.7633588
PD30716a	184.5175573
PD30717a	198.6508906
PD30718a	217.8335878
PD30719a	262.378117
PD30720a	291.1007634
PD30721a	198.0447837
PD30722a	235.4697201
PD30723a	266.5867684
PD30725a	172.2631043
PD30727a	287.464631
PD30728a	237.1211196
PD30729a	188.5796438
PD30730a	260.6300254
PD30731a	247.113486
PD30732a	199.1058524
PD30733a	261.3852417
PD30735a	362.6697201
PD30736a	349.4178117
PD30737a	269.4223919
PD30738a	332.5206107
PD30739a	393.026972
PD30740a	343.0900763
PD30741a	419.5821883
PD30742a	367.9903308
PD30743a	314.8748092
PD30744a	377.329771
PD30745a	358.3201018
PD30746a	382.1913486
PD30747a	357.1867684

PD30748a	313.3450382
PD30749a	368.9536896
PD30750a	420.6681934
PD30751a	320.8229008
PD30752a	444.4320611
PD30753a	380.2483461
PD30754a	331.4610687
PD30755a	480.23257
PD30756a	442.5760814
PD30757a	376.9058524
PD30758a	444.9033079
PD30759a	429.3709924
PD30760a	359.3750636
PD30761a	419.8956743
PD30762a	417.4147583

Supplementary Table 16. List of samples that were re-sequenced by capillary. Information about the variants re-sequenced and the result of the experiment is included. In green, samples and variants that were confirmed.

Sample	Variant tested	Result
PD30582a	p.Cys307Arg	Not present
PD30601a	p.Arg117His	Present
PD30604a	p.His393Arg	Present
PD30608a	p.Ile22Val	Present
PD30610a	p.Pro601Leu	Not present
PD30624a	p.Glu92Lys, p.Arg83Gly	Not present
PD30681a	p.Val391Ile	Not present
PD30687a	p.Gln376Ter	Not present
PD30692a	p.Trp194Ter	Not present
PD30696a	p.Pro34Ser	Not present
Case_2636	p.Asp617GlufsTer9	Present
Ctrl_1401	p.Asp617GlufsTer9	Present
Ctrl_3151	p.Asp617GlufsTer9	Present
Case_1755	p.Gln358SerfsTer13	Present
Ctrl_2641	p.Glu204Ter	Not present
Ctrl_2500	p.Asp200Asn	Not present
Case_2329	p.Asp185Glu	Not present
Ctrl_2165	p.Asp185Glu	Not present
Case_1223	p.Asp175Ter	Not present

Supplementary Table 17. List of samples with variants in known melanoma predisposition genes. Genes *CDK4*, *CDKN2A* and *BAP1* were checked for variants. Only samples that were resequenced by Illumina (Supplementary Table 15) were assessed.

Sample	*POT1* variant	Variant in pathogenic gene
PD30744a	p.Gln376Arg	*CDKN2A* Ala148Thr
PD30549a	p.Gly404Val	*CDKN2A* Ala148Thr
PD30730a	p.Gly404Val & p.Tyr419Cys	*CDKN2A* Ala60Thr
